# Multi-model collaborative early warning for battery systems with integrated fiber optic sensors

**DOI:** 10.1038/s41467-026-78461-5

**Published:** 2026-10-09

**Authors:** Jiangong Zhu, Chao Yu, Yi Jiang, Wolfgang Schade, Martin Angelmahr, Xuezhe Wei, Haifeng Dai

**Affiliations:** 1 College of Automotive and Energy Engineering, Tongji University, Shanghai, China; 2 Clean Energy Automotive Engineering Center, Tongji University, Shanghai, China; 3 Optical Sensor Systems Department, Fraunhofer Heinrich Hertz Institute, Goslar, Germany

**Keywords:** Electrical and electronic engineering, Batteries

## Abstract

The safety assessment of battery systems is constrained by sparse temperature measurements and monolithic diagnostic frameworks in conventional battery management systems (BMS). To address this, we propose a multi-model collaborative thermal-runaway early-warning framework using distributed temperature sensing. An integrated sensing scheme increases temperature sensing points within the battery pack from 32 to 540 with minimal wiring complexity and is validated in real-world vehicle operation. We establish a functional relationship between temperature Shannon entropy (SE) and average temperature variation to derive a dynamic warning boundary incorporating error tolerance. Integrating this boundary with thresholds for temperature, temperature change rate, and SE change rate enables collaborative early warning. Compared with conventional BMS warning benchmarks, the framework provides maximum warning lead times of 706 s in laboratory modules and 554 s in real-world vehicle operation. These findings demonstrate the potential of distributed sensing and collaborative diagnostics for proactive thermal-runaway risk identification.

## Abstract (web summary)

To tackle the challenge of early detection of thermal runaway in EV batteries, a distributed temperature sensing system using fiber optic sensors integrated with a multi‑model warning framework is developed and validated under real‑world vehicle operations.

## Introduction

Lithium-ion batteries (LIBs) are currently among the most widely used clean energy storage devices owing to their high specific energy and long service life, and they play a significant role in the rapidly expanding electric vehicle (EV) market^1–3^. However, safety incidents arising from battery failures are occurring with the increasing EV market, leading to substantial economic losses and exacerbating public concerns regarding the safety and reliability of EVs^4–6^. Consequently, achieving early warning of battery thermal runaway has become a critical challenge for both the research community and industry.

Currently, onboard battery management systems (BMS) and most existing battery safety assessment methods primarily rely on parallel-cell voltage measurements and sparse temperature data to evaluate battery safety^7–10^. However, as the size and capacity of LIBs, inherent heterogeneities within and between cells during operation render low-density voltage and temperature information insufficient to capture battery failure risk in a timely manner^11^. Herein, considerable research efforts have focused on increasing the density and dimensionality of battery state sensing by integrating advanced sensors either inside or outside the cell^12,13^. For example, Mei et al.^14^ integrated fiber optic sensors and Fan et al.^15^ embedded thin-film sensors within the cell, enabling real-time monitoring of internal thermal and mechanical signals. Although such approaches provide valuable data for battery safety assessment and early thermal warning, embedding foreign materials within the cell simultaneously introduces uncertainties and potentially detrimental risks to its safety and lifespan^16^. Therefore, increasing the density of external signal measurements is essential to ensure the safe and reliable operation of LIBs. In reported battery warning strategies, abnormal evolution of external thermal and mechanical signals is commonly considered an early indicator of an impending runaway^17–19^. However, vibrations and mechanical shocks during EV operation introduce substantial uncertainty in surface mechanical signal measurements, compromising their reliability as early-warning indicators.

As the physical parameter that directly reflects exothermic chain reactions of battery thermal runaway, temperature exhibits low sensitivity to external perturbations and thus offers superior robustness and practical value for onboard applications^20^. Among the reported temperature sensing techniques, non-contact methods enable spatially resolved temperature measurement by interrogating radiative energy or electromagnetic emissions from the target object^21,22^. From a measurement performance perspective, non-contact temperature sensing unquestionably represents the most effective means of acquiring high-density temperature data from LIBs. However, its integration into EVs is largely precluded by the tight packaging of the battery pack and the high cost of the required sensors^23^. In contrast, thermo-resistive devices^24,25^ and thermo-junctive devices^26,27^ are widely applied in EVs and energy storage systems due to their low cost and compact size. However, their inability to provide high-density temperature data fundamentally limits their suitability for meeting the growing temperature-monitoring demands of modern battery systems. In this context, fiber optic sensors represent an ideal solution for obtaining the spatially resolved temperature field of battery packs owing to their flexibility, multi-point sensing and immunity to electromagnetic interference^28–30^. In the existing literature, Pinto’s team was the first to integrate fiber optic sensors with LIBs, enabling measurement of battery temperature responses under both normal operation and abuse conditions^31^. Subsequent studies extended this approach to large-format LIBs, where multi-point fiber optic sensors enabled quasi-distributed mapping of surface temperature^32–34^. Building on this progress, Yu et al.^35^ employed distributed fiber optic sensors (DFOS) to obtain distributed surface temperature fields and analyze the evolution of hotspot regions during battery operation. These studies demonstrate the feasibility of integrating fiber optic sensors with LIBs to capture high-density temperature data. Nevertheless, these studies remain confined to laboratory settings, and investigations into integration design and validation under complex real-world vehicle operating conditions have rarely been reported.

In recent years, researchers have proposed a variety of approaches to address the challenges of thermal fault detection based on battery surface temperature measurements, which can be broadly categorized into three classes: threshold-based, model-based, and data-driven methods^36^. Threshold-based methods rely on predefined fixed thresholds as the decision criterion and have been widely deployed in the onboard BMS of commercial EVs owing to their low computational overhead. However, these methods can only respond to overt faults that have already exceeded the prescribed thresholds, and lack the sensitivity to detect incipient anomalous behavior when temperatures remain within acceptable bounds, resulting in a substantially elevated risk of missed detections^37^. Given the inability of threshold-based methods to capture the intrinsic physical mechanisms governing battery behavior, several studies have turned to model-based approaches that diagnose thermal faults by constructing electrochemical or thermal models and exploiting the residual deviation between model predictions and actual measurements. While such methods offer strong physical interpretability, their diagnostic performance is critically dependent on the high-fidelity physical model. More importantly, the relatively low data sampling rates of mainstream onboard BMS fundamentally constrain the achievable model accuracy, severely limiting the practical engineering applicability of these methods under real-world driving conditions^38,39^. To overcome the dual dependency of the two methods on prior knowledge and high-fidelity physical models, data-driven methods have garnered increasing research attention. This class of approaches encompasses fault feature extraction based on signal processing techniques^40^ as well as the development of anomaly recognition models based on machine learning algorithms^41^. However, signal-processing-based feature extraction methods still predominantly rely on fixed thresholds for anomaly discrimination and therefore have not fundamentally escaped the inherent limitations of threshold-based approaches. Meanwhile, despite machine learning algorithms having superior nonlinear modeling capacity, they face substantial challenges for practical deployment due to their high computational cost and training complexity.

**Fig. 1:**
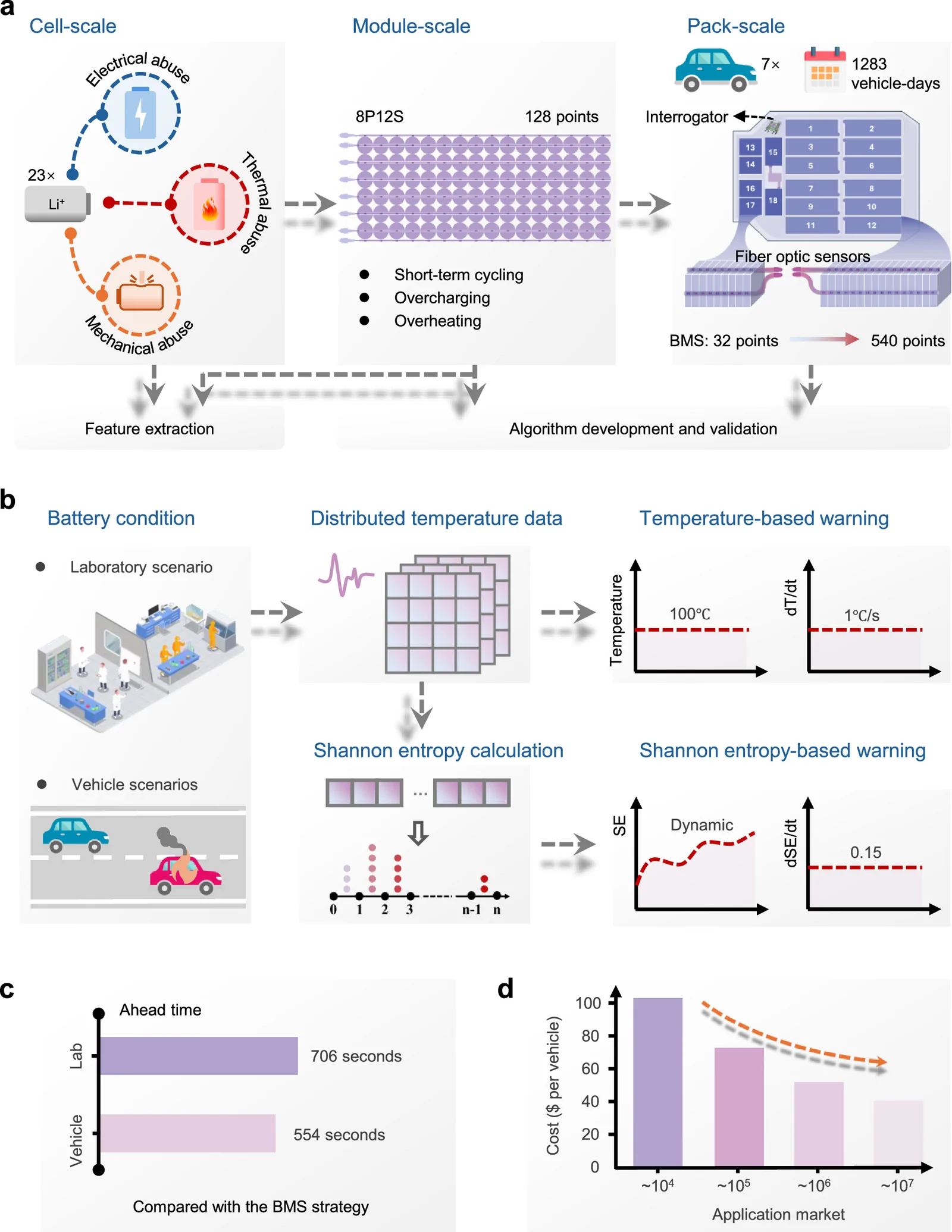
Early warning of battery thermal runaway based on distributed temperature data measured by fiber optic sensors. **a** Temperature datasets at the cell-level, module-level and pack-level. **b** Multi-model battery thermal-runaway warning framework, including temperature-based and SE-based strategies. **c** The lead time of the proposed early-warning framework in issuing warning signals, benchmarked against the warning methods integrated in conventional BMS. **d** The cost of the proposed warning scheme versus the EV application market, and the detailed cost analysis is provided in Supplementary Note 1. (Calendar icon in Fig. 1a and car icon in Fig. 1b: designed by magnific from flaticon; laboratory illustration in Fig. 1b: designed by macrovector–Magnific.com.).

As illustrated in Fig. 1, this study employs fiber optic sensors assembled from femtosecond-laser-inscribed Fiber Bragg Gratings (FBGs) to achieve distributed temperature sensing within the battery system with minimal wiring complexity, substantially enhancing the observability of the internal thermal field (the number of temperature-sensing points increased from 32 to 540). More critically, the fiber optic sensors maintained stable operation across seven EVs over individual monitoring periods ranging from 25 to 410 days, totaling 1284 vehicle-days, thereby confirming the engineering feasibility of the integration scheme developed in this study. Furthermore, through systematic analysis of temperature evolution in both battery cells and battery modules under normal and abusive conditions, this study establishes a functional relationship between temperature shannon entropy (SE) and average temperature, on which a dynamic SE warning boundary is constructed. In conjunction with the designed temperature threshold, temperature change rate threshold, and SE change rate threshold, a multi-model collaborative thermal-runaway early-warning framework based on distributed temperature sensing is formulated. The core advantages of this framework are manifested in two aspects. First, the constructed dynamic SE warning boundary inherits the intrinsic merits of signal-processing-based feature extraction methods, namely, independence from prior knowledge and high-fidelity physical models, while transcending the inherent limitation of conventional signal-processing approaches that rely on fixed thresholds for anomaly discrimination, thereby enabling adaptive adjustment of the warning boundary in accordance with the real-time operational state of the battery. Second, the multi-model collaborative mechanism systematically reduces the risks of false alarms and missed detections associated with single-model decision-making by fully leveraging their complementary characteristics, effectively enhancing the overall reliability of the warning system. Using the warning results of conventional BMS as a baseline, the proposed strategy achieves a maximum early-warning lead time of 706 s at the lab-scale module level and up to 554 s on real-world operational data. In addition, according to statistics from the International Energy Agency, global EV sales exceeded 20 million units in 2025^42^. Benchmarked against this market scale, the per-vehicle application cost of the proposed warning scheme is only ~36.82 USD, demonstrating both strong cost-effectiveness and substantial prospects for large-scale commercialization (see Supplementary Note 1). In summary, this work mitigates the limitations imposed by the low density of temperature data and the reliance on a single simple diagnosis model in the current BMS, providing a practical pathway toward enhanced operational safety and reliability.

## Results

### Experiments and data analysis

**Fig. 2:**
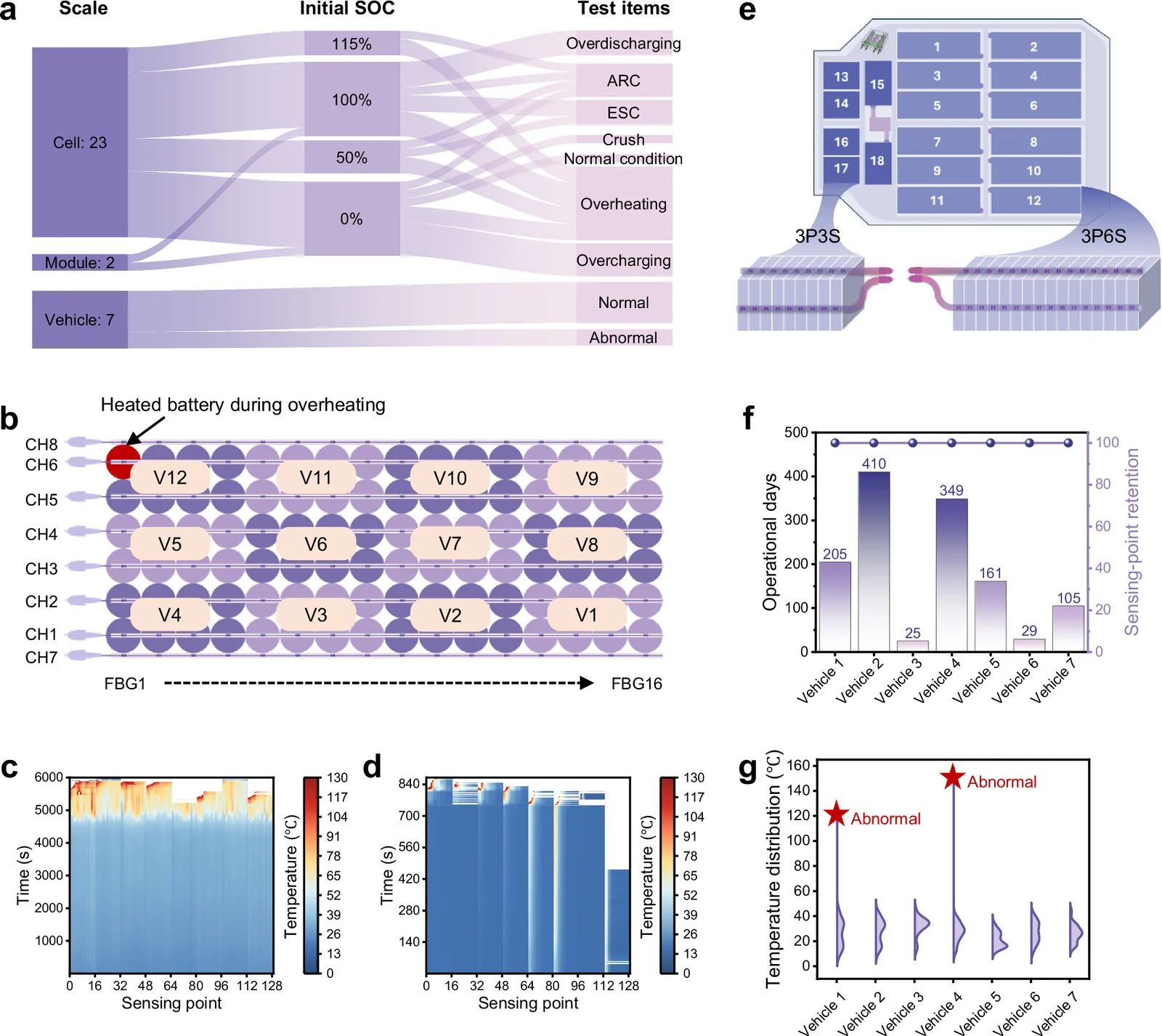
Overview of multiscale LIB dataset acquisition and analysis. **a** Sankey plot showing battery testing tasks in the laboratory and the operating conditions of EVs integrated with fiber optic sensors under real-world vehicle scenarios. **b** Illustration of the integration scheme between fiber optic sensors and the 18,650 cylindrical battery modules in the laboratory, with the red-marked cell denoting the one heated by a heating wire during the overheating test. **c** Temperature evolution at each sensing point of the battery module during the overcharging test. **d** Temperature evolution at each sensing point of the battery module during the overheating test. **e** Battery pack configuration and integration scheme of fiber optic sensors. **f** Effective operating days of EVs equipped with the battery pack integrated with fiber optic sensors, and retention of effective temperature sensing points before and after operation. **g** Temperature distribution within the battery pack during operation across seven vehicles, where “Vehicle 1” and “Vehicle 4” ultimately undergo thermal runaway.

To support the development and performance validation of the proposed early-warning strategy, a comprehensive set of experimental tests encompassing both normal cycling and abuse conditions are conducted at the lab-scale on 23 battery cells and two battery modules integrated with fiber optic sensors, detailed specifications of LIBs employed are given in Supplementary Table 1. Building upon this foundation, the integration scheme for fiber optic sensors with battery modules is further extended and deployed in the battery packs of seven EVs, two of which ultimately experienced thermal runaway. All experimental configurations and vehicle operational status are summarized in Fig. 2a. Specifically, the two battery modules investigated in this study consist of 96 cylindrical LIBs configured in an 8-parallel, 12-series (8P12S) arrangement, with a nominal capacity of ~27 Ah, and the inconsistency analysis results for the two modules are provided in Supplementary Note 2. Following the integration scheme illustrated in Fig. 2b, eight fiber optic sensors are integrated into each module, enabling real-time acquisition of temperature data at 128 spatial locations. As shown in Fig. 2c, the temperature evolution recorded across all sensing points during the overcharging test of the battery module with an initial state of charge (SOC) of 0% reveals a pronounced increase in the spatial dispersion of module temperature during the period surrounding the thermal-runaway onset. Complementarily, the spatial temperature distribution captured during the localized overheating-induced thermal-runaway test demonstrates that sensing points on the faulted cell and its immediately adjacent cells exhibit substantially accelerated thermal responses relative to those at distal positions, as illustrated in Fig. 2d. Taken together, these experimental observations provide compelling evidence that high spatial density in battery temperature measurements is indispensable for achieving reliable fault localization and timely thermal-runaway warning.

To further demonstrate the temperature-sensing capability and thermal-runaway early-warning performance of the proposed strategy under real-world vehicle operation, seven EVs are selected in this study and labeled as “Vehicle 1” through “Vehicle 7”. The battery pack deployed in each vehicle consists of six 3P3S modules and twelve 3P6S modules, comprising a total of 270 cells, as shown in Fig. 2e. According to the module architecture of the battery pack and the signal transmission characteristics of the fiber optic sensors, two fiber optic sensors are installed on the module sidewall and tab regions, respectively. Figure 2f summarizes the effective operational days of each vehicle and retention of effective temperature sensing points within the battery pack before and after operation, which remained unchanged for all vehicles during operation, indicating that the current integration scheme of the fiber optic sensors with the battery pack exhibits good stability under real-world vehicle operation. Figure 2g summarizes the overall temperature distribution of battery packs derived from the complete temperature dataset collected during real-world vehicle operation. The results show that the temperature distribution of the battery pack is predominantly within the range of 0-50 °C under normal operating conditions. Nevertheless, markedly elevated abnormal temperature signals are observed in “Vehicle 1” and “Vehicle 4”, corresponding to thermal runaway during charging and during post-full-charge operation, respectively. Additional details regarding the temperature data of the vehicle battery packs are provided in Supplementary Note 3.

### Temperature-dependent characteristics of the battery

**Fig. 3:**
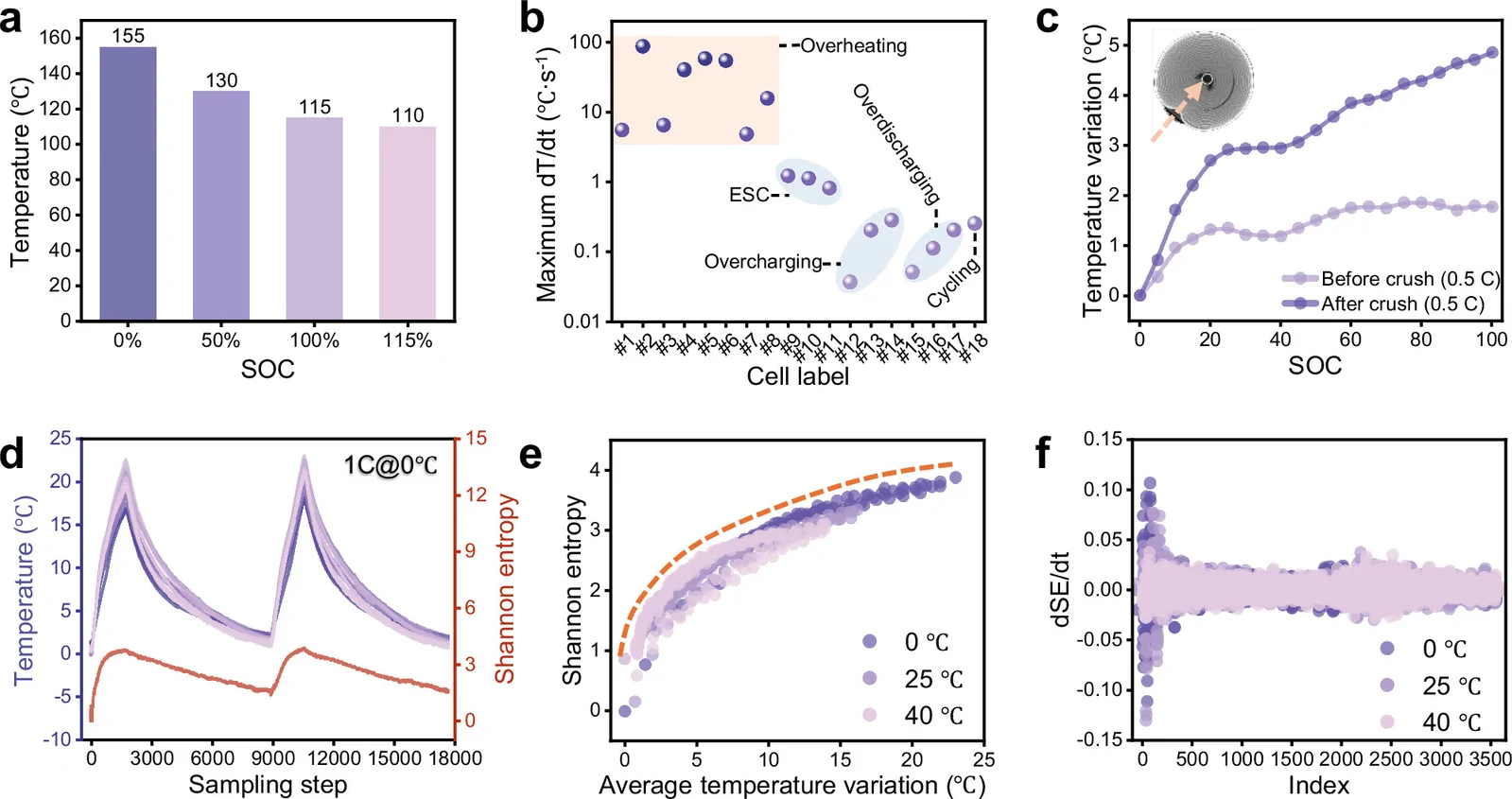
Temperature features extracted from cell-level and module-level experimental datasets. **a** The onset temperature of battery thermal runaway versus battery SOC. **b** Maximum surface temperature change rates of battery cells during normal cycling and abuse conditions, with the detailed testing protocols for individual cells provided in Supplementary Table 2. **c** Surface temperature evolution during 0.5C constant-current charging before and after mechanical crush. **d** Evolution of module temperature distribution and the corresponding temperature SE during 1C constant-current charging at 0 °C. **e** Temperature SE of battery module versus average temperature variation. **f** Temporal rate of change in temperature SE under different operating conditions.

To extract temperature features that capture early thermal anomalies preceding thermal runaway, the surface temperature evolution of battery cells and modules under a comprehensive set of abuse conditions is systematically analyzed. Figure 3a presents the onset temperature of battery thermal runaway across various SOCs, exhibiting a trend consistent with that widely reported in the literature: LIBs at higher SOCs generally display reduced thermal stability^43^. Figure 3b summarizes the maximum surface temperature change rate recorded during normal cycling and under different abuse conditions. It is evident that lower short-circuit resistance and higher current rates both lead to substantially elevated rates of surface temperature change. Notably, the rate of surface temperature change induced by normal cycling at high current rates can even exceed that observed under several overcharging and overdischarging conditions. This finding indicates that relying solely on surface temperature and its rate of change as inputs is inadequate for accurate early warning of battery thermal runaway, thereby further motivating the development of a multi-model warning framework with broad failure-mode coverage. Regarding the cell with a micro internal short circuit induced by mechanical crushing, its surface temperature evolution during subsequent 0.5C (1 C is equal to the nominal capacity of the used batteries) constant-current charging is distinctly different from that observed prior to crushing, as shown in Fig. 3c. Moreover, the corresponding surface temperature evolution under other current rates and current directions is provided in Supplementary Fig. 1. Building on this observation, SE is introduced in this work to quantitatively characterize the spatial dispersion of temperature data within the battery system, thereby enabling the identification of abnormal thermal evolution. Nevertheless, the inherent temperature inconsistency in battery systems renders fixed SE thresholds inadequate for adapting to the dynamic variability of operating conditions. Furthermore, the analysis of both the overall temperature SE and the temperature reveals that the entropy evolution closely tracks the evolution of the module temperature, as illustrated in Fig. 3d. Guided by this correlation, a mapping relationship between the temperature SE of battery modules and its average temperature variation is further established, which exhibits an overall parabolic trend, as shown in Fig. 3e. Finally, Fig. 3f presents statistical distributions of the SE change rate under various operating conditions, all of which are found to fall within well-defined bounded intervals.

### Warning framework overview

**Fig. 4:**
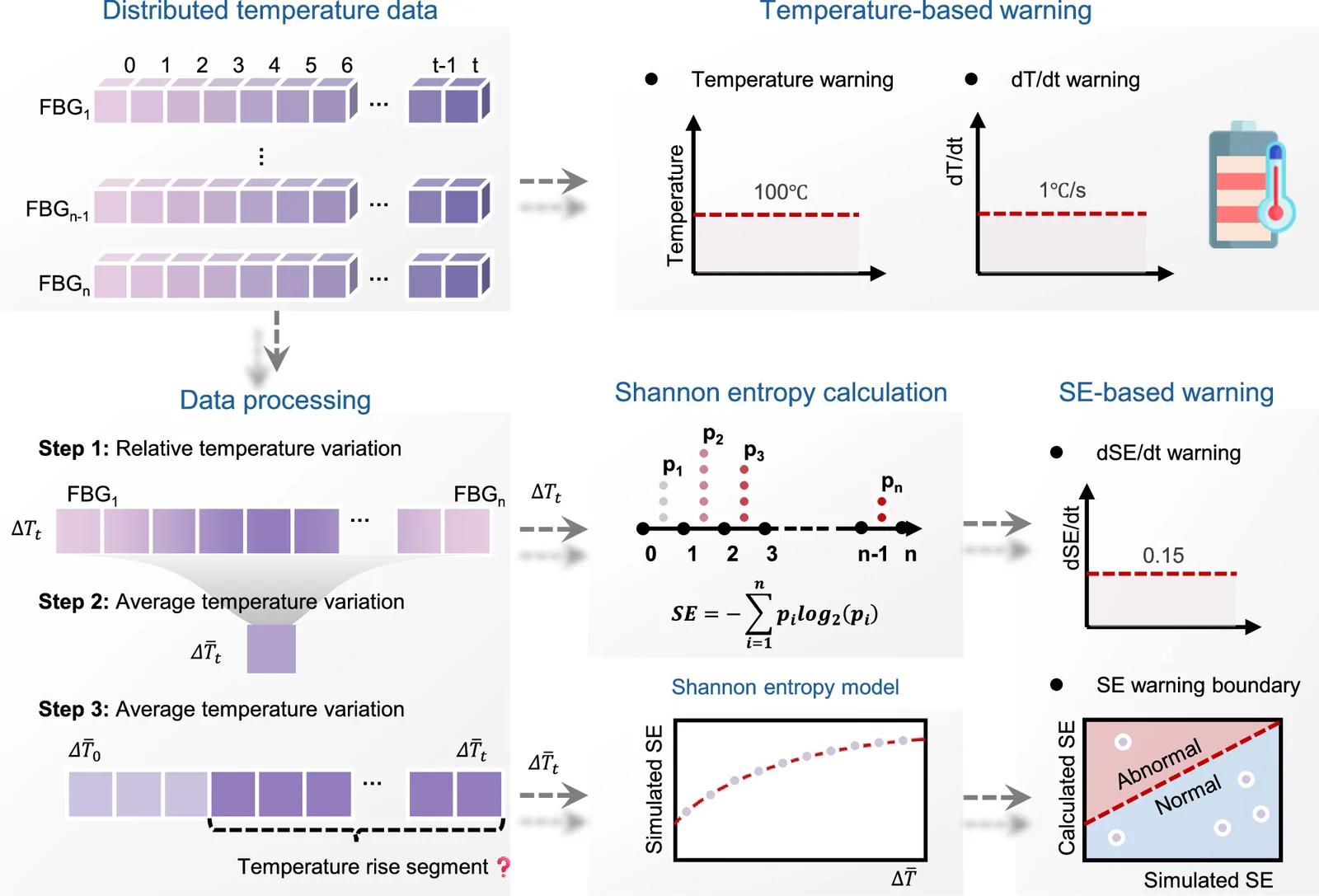
Multi-model early-warning framework for battery thermal runaway based on distributed temperature sensing. (Temperature icon in Fig. 4: designed by Magnific from Flaticon).

Figure 4 shows the multi-model collaborative thermal-runaway warning framework developed in this study using distributed temperature sensing from the battery system. The framework integrates two core strategies: a temperature-based warning strategy and a Shannon-entropy-based warning strategy. The temperature-based strategy provides a rapid assessment of thermal-runaway risk by comparing fiber optic sensor measurements with predefined thresholds for temperature and temperature change rate. In the thermal-runaway mechanisms of most LIBs, the onset of self-generated heat marks the upper thermal limit for safe operation and is closely linked to the decomposition of the solid electrolyte interphase (SEI). The initial SEI decomposition generally occurs in the temperature range of 80–120 °C, with a decomposition peak located at ~100 °C^44^. In addition, the Accelerating Rate Calorimetry (ARC) test results of fresh cylindrical cells in this study indicate that the onset temperature of self-generated heat falls within the range of ~110–155 °C, but this temperature is expected to decrease with battery aging^45^. Hence, the temperature threshold for thermal-runaway warning is set to 100 °C, following a comprehensive consideration of the above factors. Meanwhile, considering the maximum temperature variation rates obtained from a series of normal and abuse condition tests conducted in this work, together with widely reported criteria for identifying battery thermal runaway in the literature^46,47^, a temperature change rate of 1 °C/s is adopted as the warning threshold for battery thermal runaway. For the Shannon-entropy-based warning strategy, temperature-rise segments are first identified based on the rate of change of the average temperature of the battery system. The instantaneous temperature SE and its temporal rate of change are then calculated for each identified segment, and the entropy warning boundary is determined based on the corresponding average temperature variation of the battery system (see Methods). Based on this, a thermal-runaway risk is identified when the real-time temperature SE exceeds this theoretical boundary. Furthermore, based on the statistical distribution of the SE change rate under various operating conditions, a threshold of 0.15 for the SE change rate is adopted as an identification criterion for battery thermal runaway. Building upon this, the four warning criteria described above operate independently and in parallel. A thermal-runaway warning signal is issued immediately upon triggering any one criterion, without requiring the remaining criteria to be satisfied simultaneously.

### Thermal runaway warning performance

**Fig. 5:**
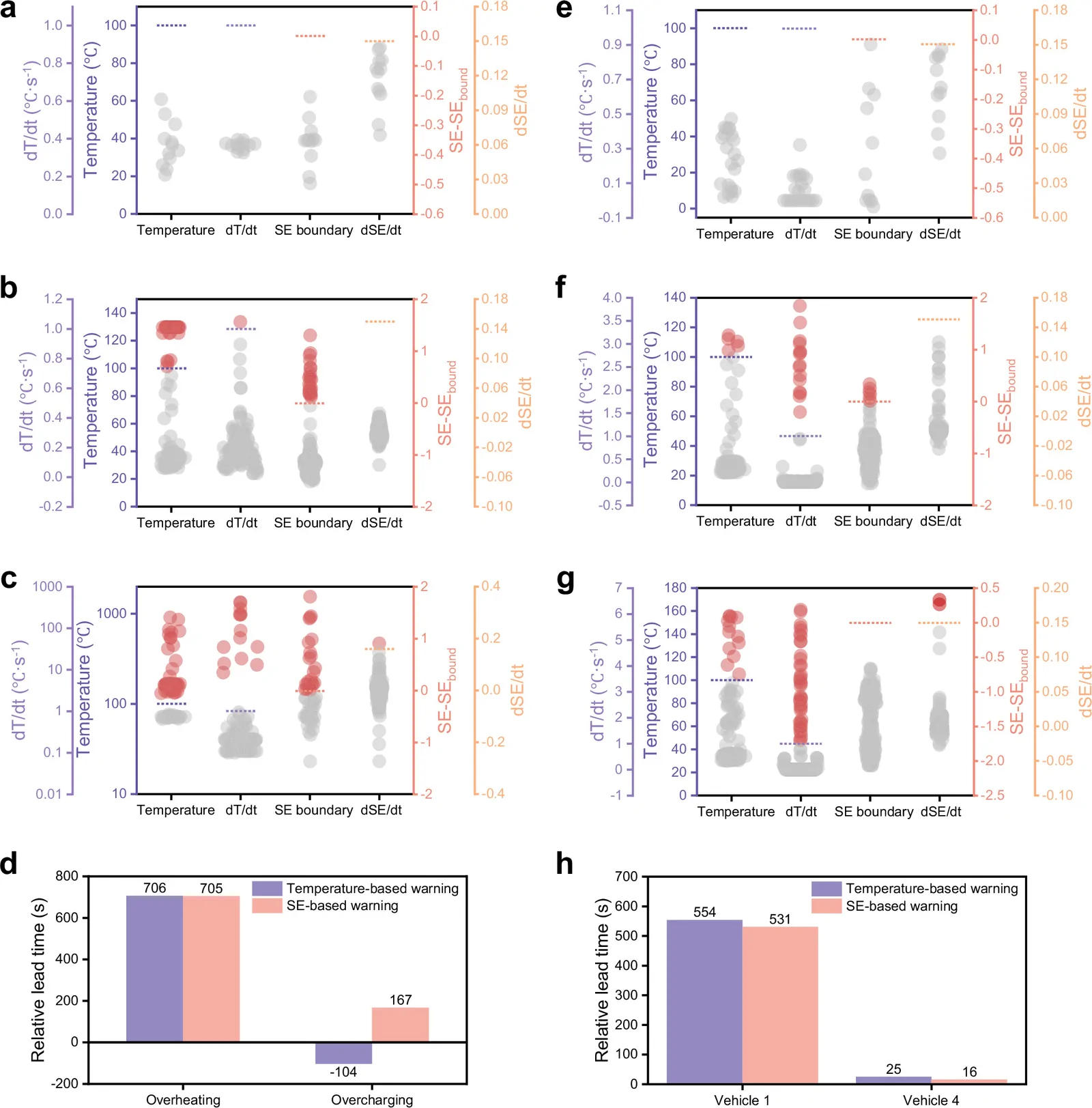
Performance evaluation of the multi-model collaborative thermal-runaway warning framework. **a**–**c** Warning performance for battery modules under normal operation (**a**), overcharging-induced thermal runaway (**b**), and overheating-induced thermal runaway (**c**) in laboratory scenarios. **d** Relative warning lead times achieved by the two warning strategies in the proposed framework under different thermal-runaway triggering modes in laboratory tests. **e**–**g** Warning performance for non-thermal-runaway vehicles (**e**), “Vehicle 1” (**f**), and “Vehicle 4” (**g**) under real-world operating conditions. **h** Relative warning lead times achieved by the two warning strategies in the proposed framework for different faulty vehicles under real-world scenarios.

Figure 5a–c, e–g illustrate the warning results of the proposed warning framework on module-level testing datasets from laboratory scenarios and pack-level operating datasets from real-world vehicle scenarios, respectively. For battery systems without thermal runaway, all warning models in the framework remained inactive, demonstrating a lower false-alarm propensity. For battery systems undergoing thermal runaway, although some individual warning models in the framework fail to identify fault signatures in a timely manner, the overall warning framework consistently generates abnormal warning signals. Supplementary Fig. 2 illustrates the parallel-cell voltage evolution of battery modules and packs that experienced thermal runaway. Using a voltage variation rate of 1 V/s for the parallel-cell voltage as the reference criterion for fault warning in the conventional BMS, the relative warning lead times achieved by the proposed framework are shown in Fig. 5d, h. For module-level thermal runaway triggered by localized overheating, both core strategies of the framework provided warning signals >700 s in advance. In contrast, for the module-level thermal runaway triggered by overcharging, although both core warning strategies generate warning signals and the Shannon-entropy-based strategy achieves a warning lead time of ~167 s, the temperature evolution during overcharging is relatively moderate compared with the pronounced temperature variations observed during the overheating test. Consequently, the warning time of the temperature-based strategy is even later than that of the reference voltage-based method, further highlighting the importance of a multi-model collaborative warning framework. Furthermore, in real-world vehicle scenarios, the proposed framework achieves a relative warning lead time of 554 s for “Vehicle 1”, substantially longer than the 25 s obtained for “Vehicle 4”. This difference is mainly attributed to differences in the failure modes and fault propagation severity of various battery systems.

To further evaluate the impact of the spatial density of temperature measurements on thermal runaway warning performance, we compare sparse temperature measurements available from a conventional BMS with the high-density temperature measurements obtained in this study, while applying the same warning framework to both datasets, as summarized in Table 1. Compared with sparse temperature information available from a conventional BMS, high-density temperature sensing generally enables earlier detection of abnormal thermal behavior within the battery system and reduces the risk of missed warnings in several failure scenarios. This advantage is particularly pronounced for localized thermal anomalies, because a limited number of temperature sensing points may fail to capture abnormal behavior confined to a small spatial region, whereas high-density temperature measurements substantially enhance the observability of both local thermal anomalies and spatial temperature heterogeneity. In addition, the fiber optic sensors employed in this work exhibit a faster thermal response than the thermocouples (see Supplementary Fig. 3), which further enhances the capability to capture transient thermal anomalies. These results demonstrate that increasing the spatial density of temperature measurements can substantially improve the timeliness and reliability of thermal-runaway warning. The parameter settings of the warning framework when using the sparse temperature measurements available from a conventional BMS are provided in Supplementary Table 3.

**Table 1:** Performance of a given thermal-runaway warning strategy with different temperature inputs

Warning strategy		Warning time			
		Overheating	Overcharging	Vehicle 1	Vehicle 4
Sparse temperature data acquired by the BMS	Temperature-based warning	14:05:37	11:18:22	None	19:11:54
	SE-based warning	14:07:18	None	17:04:30	None
High-density temperature data used in this work	Temperature-based warning	13:53:42	11:15:21	17:02:32	19:11:29
	SE-based warning	13:53:43	11:10:50	17:02:41	19:11:42

Furthermore, to further benchmark the proposed warning framework, distributed temperature data are used as inputs to compare its warning performance with three thermal-runaway warning strategies reported in the existing literature, as summarized in Table 2. Although the temperature-based composite threshold strategy^48^ successfully identifies all thermal runaway events, its warning times are consistently later than those of the proposed framework. Moreover, neither the temperature variance and local overheating index (LOI) dual-threshold strategy^49^ nor the modified standardized cumulative sum (CUSUM) method^50^ detected all thermal runaway events. Collectively, these results demonstrate that the proposed framework achieves a more favorable overall performance in terms of both warning timeliness and detection reliability than the benchmark strategies. This improvement is primarily attributable to the SE model, which reduces reliance on fixed thresholds for anomaly identification, and to the multi-model collaborative warning mechanism, which mitigates the risks of false alarms and missed detections associated with decisions based on a single model. Detailed descriptions and parameter settings of the three benchmark warning methods reported in existing literature are provided in Supplementary Note 4.

**Table 2:** Performance comparison of existing warning methods using distributed temperature sensing as input

Warning strategy	Warning time			
	Overheating	Overcharging	Vehicle 1	Vehicle 4
Temperature-based composite threshold strategy^48^	13:53:45	11:15:21	17:03:00	19:11:52
Temperature variance and LOI dual-threshold warning^49^	14:05:31	None	None	None
Modified standardized CUSUM method^50^	13:53:43	11:18:24	None	None
The proposed warning framework	13:53:42	11:10:50	17:02:32	19:11:29

Collectively, these results disentangle the respective contributions of high-density temperature sensing and the multi-model collaborative warning framework to thermal runaway warning performance. High-density temperature sensing enhances the observability of localized and spatially heterogeneous thermal anomalies, whereas the proposed warning framework further translates this sensing advantage into earlier and more reliable warning performance across different failure modes. The integration of high-density sensing with the multi-model collaborative warning framework, therefore, provides a more reliable basis for early warning of thermal runaway than conventional sparse temperature monitoring combined with a single warning strategy.

### Onboard implementation feasibility

The onboard resource requirements of the proposed warning framework are further evaluated in terms of memory usage and computational demand. Specifically, the pack-level sensing scheme developed in this study comprises 540 temperature-sensing points, and the longest sliding window used in the warning framework comprises 500 samples. With 32-bit floating-point representation, the corresponding temperature data buffer occupies 1.08 MB. After further accounting for reference temperatures, intermediate variables, communication buffers, and the software stack, an on-chip static random-access memory (SRAM) capacity of 2 MB represents a reasonable hardware target for real-time implementation. In terms of computational demand, the proposed framework primarily involves linear least-squares fitting, threshold comparisons, and SE calculation. Depending on the number of temperature-sensing points and their corresponding window lengths, each 1 Hz update requires approximately basic arithmetic and comparison operations. Under a conservative upper-bound assumption, the SE calculation additionally involves at most 540 logarithmic evaluations, while evaluation of the dynamic entropy boundary requires only one exponential operation. Using conservative processor-cycle assumptions, the computational demand of a complete warning update is estimated at on the order of 10^6^ processor clock cycles. After introducing a tenfold engineering margin for communication, diagnostics, operating-system tasks, and other BMS functions, the resulting computational budget remains ~10^7^ processor clock cycles per 1 Hz update. Accordingly, an automotive-grade real-time processor with at least 2 MB of on-chip SRAM and a clock frequency on the order of 10 MHz or higher provides substantial hardware margin for onboard implementation of the proposed warning framework. Such requirements are within the capabilities of commercially available automotive-grade processors^51,52^. In future work, the proposed warning algorithm will be implemented on automotive-grade hardware and its real-time execution performance evaluated under real-world vehicle operating conditions.

## Discussion

We have demonstrated the efficacy of a multi-model collaborative thermal-runaway early-warning framework based on distributed temperature sensing in assessing the safety of battery systems. The success of this method can be attributed to two synergistic factors. On the one hand, the multi-point sensing capability of fiber optic sensors along a single fiber enables spatially distributed temperature sensing throughout the battery system with minimal wiring complexity. On the other hand, the multi-model collaborative early-warning framework, built on the characteristic thermal evolution patterns of battery surfaces under distinct failure conditions, enhances the reliability of early thermal-runaway identification through complementary decision-making. Diverging from the warning strategies of conventional BMS, which rely on the sparse temperature information and the monolithic diagnostic frameworks, the approach proposed herein places explicit emphasis on the synergistic coupling of high-density distributed temperature sensing and multi-model collaborative diagnostics. The designed scheme for integrating fiber optic sensors into battery systems is validated in real-world vehicle operation for the first time, with seven instrumented EVs operating over periods of up to two years, totaling 1284 vehicle-days, confirming their robustness and engineering readiness for practical battery system deployment.

In summary, the multi-model collaborative thermal-runaway early-warning framework based on distributed temperature sensing proposed in this study demonstrates clear potential to accurately, reliably, and engineering-feasibly enhance the safety of battery systems. A distributed temperature sensing system based on fiber optic sensors is constructed, expanding the number of temperature-sensing points in the battery systems employed in this study from 32 to 540, thereby providing high-density temperature information that substantially surpasses the limited temperature-sensing capability of current BMS for assessing battery system safety. Leveraging distributed temperature data collected from battery systems, we establish a functional relationship between the temperature SE and the average temperature variation, on which a dynamic SE warning boundary is further developed by introducing an error-tolerance mechanism. Combined with the temperature threshold, the temperature change rate threshold, and the SE change rate threshold, this yields a multi-model collaborative thermal-runaway early-warning framework that operates independently of prior battery knowledge and complex physical models. Using a 1 V/s voltage variation rate for the parallel-cell voltage as the reference criterion for fault warning in the conventional BMS, the proposed framework achieves a maximum thermal-runaway early-warning lead time of 706 s at the lab-scale module level and up to 554 s under real-world vehicle operational data. Taken together, the methodology presented in this work mitigates the dual limitations of insufficient thermal sensing density and constrained diagnostic reliability of single models inherent to current BMS, providing a practically viable pathway toward enhanced operational safety and reliability of onboard battery systems.

Although the results of this study are promising, certain limitations warrant further investigation. The number of failure samples and the diversity of failure modes covered in the validation remain limited, and the applicability of the proposed framework to internal short circuits, mechanical damage and other complex failure modes requires further validation. Furthermore, the effects of battery aging, different material chemistries, and system architectures on the stability of the developed SE model have not yet been systematically evaluated, warranting further investigation into its transferability across battery systems and operating conditions. Hence, future work will expand the calibration and validation datasets across the aforementioned factors to establish a model parameter library, enabling parameter updates through over-the-air (OTA) deployment and thereby improving the generalizability of the warning framework across different battery systems and operating conditions.

## Methods

### Normal cycling and abuse condition testing

At the cell level, this study systematically investigates the thermal behavior of LIBs under various conditions, including overheating tests, external short circuit (ESC) tests, ARC tests, mechanical crush tests, overcharging tests, overdischarging tests and constant-current charging-discharging tests. In the overheating tests, two different triggering methods (heating rod and heating wire) are employed to heat four battery cells with different initial SOCs of 0%, 50%, 100%, and 115%. Supplementary Fig. 4a and Supplementary Fig. 4b present schematics of experimental setups and sensor layouts for the heating-rod-triggered and heating-wire-triggered configurations, respectively. Furthermore, the diameter and height of the heating rod are 18 mm and 65 mm, respectively. The diameter and length of the heating wire are 0.4 mm and 550 mm, respectively. The heating power is fixed at 80 W for both triggering ways. Supplementary Fig. 4c illustrates the experimental setup for the ESC tests; three LIBs with initial SOCs of 50%, 100%, and 100% are connected to short-circuit resistances of 20 mΩ, 20 mΩ, and 50 mΩ, respectively. ARC tests are performed on four LIBs with initial SOCs of 0%, 50%, 100%, and 115% using a BAC-90A system manufactured by Young Instruments, as shown in Supplementary Fig. 4a. The cell is first heated to 50 °C and maintained at this temperature for 60 minutes. Subsequently, the temperature is increased in increments of 5 °C and then maintained for 30 mins to ensure the cell reaches thermal equilibrium, and then check whether the temperature rise rate exceeds the detection threshold of 0.02 °C/min. If not, the “heat-wait-seek” protocol will be repeated until exothermic reaction detection^53^. Supplementary Fig. 4e shows the experimental setup for the mechanical crush tests; the cell with an initial SOC of 0% is fixed to the test platform. A hemispherical indenter with a diameter of 3 mm is then used to crush the center of the cell at a loading rate of 6 mm/min, and the test is terminated once a voltage drop exceeding 100 mV is detected. Supplementary Fig. 4f illustrates the experimental platform for battery overcharging, overdischarging and constant-current charging/discharging tests. A Neware CE-6002n-60V100A-H battery tester is used for charging and discharging processes. For the overcharging-induced thermal runaway tests, three battery cells with an initial SOC of 0% are charged at constant currents of 0.5C, 1C, and 2C, respectively, until a significant abnormal voltage drop is observed. For the overdischarging-induced thermal runaway tests, three battery cells with an initial SOC of 100% are discharged at constant currents of 0.5C, 1C, and 2C, respectively, until an abnormal voltage drop occurs. Finally, a 4C current is applied to one cell to conduct constant-current charging–discharging tests. To ensure comparability across all experiments, the capacities of the cells used in the mentioned tests are closely matched.

At the module level, overheating- and overcharging-induced thermal-runaway tests are conducted in this study. Before conducting the module-level overheating-induced thermal-runaway tests, the battery module is preformed to a constant-current charging-discharging test at a current of 0.5C and 1C under various ambient temperatures (0 °C, 5 °C, 10 °C, 15 °C, 25 °C, 40 °C), the ambient temperature is controlled by the thermal chamber), and a rest period of 2 hours is applied after both the charging and discharging steps. To simulate the battery operating window in real-world vehicle applications, the upper and lower cut-off voltages during normal charging–discharging are set to 48 V and 34 V, respectively. Subsequently, the overheating test is triggered by heating a single cell using the heating wire; the heated cell is red-marked in Fig. 2b, with the heating power of 160 W. The diameter and length of the heating wire are 0.4 mm and 550 mm, respectively. Furthermore, the overcharging test is performed by applying a current of 28 A to the module with an initial SOC of 0%, until fire or explosion is observed.

### Calibration of the fiber optic sensors

The fiber optic sensors assembled with femtosecond-laser-inscribed FBGs used in this study are manufactured by SensChain Co., Ltd. To eliminate the influence of mechanical strain on the temperature measurements of the fiber optic sensors, all sensors are enclosed within a Poly-Tetra-Fluoro-Ethylene (PTFE) tube with an outer diameter of 0.9 mm and an inner diameter of 0.4 mm. Subsequently, the temperature calibration of the fiber optic sensors is conducted in a high/low-temperature environmental chamber from 40 °C to 180 °C, with a step of 10 °C and a rest time of 30 minutes at each step. The calibration results for several fiber optic sensors employed in this study are shown in Supplementary Fig. 5. Within the calibrated temperature range, the reflected wavelength of the FBG varies linearly with temperature, and the slope corresponds to the temperature sensitivity coefficient. It can be observed that the temperature sensitivity coefficients of different FBGs exhibit good consistency, at ~11 pm/°C.

### Integration of fiber optic sensors into the battery system

In accordance with the designed integration scheme for the fiber optic sensors with the battery system, the PTFE-tube-encapsulated fiber optic sensors are first fixed at the predefined measurement locations of the battery system using adhesive tape. Subsequently, 3 M™ Scotch-Weld™ epoxy adhesive DP100 is applied at the grating region for final fixation. This adhesive achieves initial curing within 5 minutes and exhibits good shear strength (~10.3 Mpa) after complete curing for 24 hours, enabling it to withstand the complex vibration conditions encountered in real-world vehicle scenarios. Subsequently, all fiber-optic sensors integrated into the battery pack are connected to an onboard miniaturized interrogator (developed by SensChain Co., Ltd.) for distributed temperature-field acquisition. The dimensions of the interrogator and its installation location within the battery pack are illustrated in Supplementary Fig. 6. To further evaluate the influence of the applied fixation scheme on the temperature sensing performance of the fiber optic sensors, a thermocouple and a fiber optic sensor are arranged in parallel on the sidewall of a cylindrical cell subjected to ARC testing. The fiber optic sensor is fixed using the aforementioned scheme, whereas the thermocouple is fixed with polyimide high-temperature adhesive tape. The temperature evolution obtained by the two types of sensors during the ARC testing is shown in Supplementary Fig. 7. It can be seen that the two temperature profiles are in good agreement, indicating that the encapsulation and fixation scheme designed in this study does not adversely affect the temperature measurement performance of the fiber optic sensor.

### Shannon-entropy-based warning strategy

#### SE calculation

SE is an indicator employed to quantify the uncertainty of a random variable and increases with increasing data uncertainty, it is defined as^54^:

1 $$SE=-{\sum}_{i=1}^{n}{p}_{i}{\log }_{2}({p}_{i})$$

where n denotes the number of data intervals, and ${p}_{i}$ is the probability that a sampling point falls within the i-th interval. To mitigate the influence of inconsistent initial temperatures across different sensing points on the SE calculation, the temperature variation ΔT relative to the initial time is utilized as the input, which is calculated as follows:

2 $$\varDelta T=\left[\begin{array}{ccc}0 & \cdots & 0\\ \vdots & & \vdots \\ \varDelta {T}_{t,1} & \cdots & \varDelta {T}_{t,m}\end{array}\right]=\left[\begin{array}{ccc}{T}_{1,1} & \cdots & {T}_{1,m}\\ \vdots & & \vdots \\ {T}_{t,1} & \cdots & {T}_{t,m}\end{array}\right]-\left[\begin{array}{ccc}{T}_{1,1} & \cdots & {T}_{1,m}\end{array}\right]$$

where the subscripts t and m denote the *t-*th time step and the m-th temperature sensing point, respectively. For the SE calculation, the instantaneous temperature range of the battery system is determined by rounding down the minimum value and rounding up the maximum value of the distributed temperature data at each time step. The resulting range is then divided into multiple equal intervals, each with a bin width of 1 °C, to obtain the temperature probability distribution.

#### Identification of the temperature rise segment

Given that prolonged rest allows the overall temperature field of the battery system under real-world vehicle operation to gradually approach a relatively stable state, when the time gap between two consecutive data records exceeds 30 minutes, the subsequent temperature data are treated as a new independent temperature segment in this work. For each segment, the temperatures measured at all sensing locations at the initial time point are taken as new references, such that the temperature variation at each location is reset to 0. Based on this reference, the average temperature variation across all valid temperature-sensing points is calculated, and its local temporal trend is characterized using linear least-squares fitting within a sliding window of 500 samples with a step size of 1 sample. For each independent temperature segment, the continuous interval starting from the reference time during which the fitted slope remained non-negative (k≥0) is defined as a temperature rise segment. The segment is considered to terminate when the fitted slope first became negative (k<0).

#### SE warning boundary

In accordance with the mapping relationship between temperature SE and the average temperature variation of the battery system shown in Fig. 3e, their nonlinear dependence is fitted using Eq. (3) in this study. Meanwhile, an error-tolerance mechanism is further introduced to establish the temperature SE warning boundary.

3 $$F(x)=a{e}^{-bx}+c$$

4 $$SEB=F(x)+{E}_{{{\mathrm{tol}}}}$$

where a, b and c are fitting parameters for the nonlinear relationship between temperature SE and the average temperature variation, ${E}_{{{\mathrm{tol}}}}$ denotes the error tolerance. x is the average temperature variation ($\varDelta \bar{T}$), whose expression is given as follows:

5 $$\varDelta \bar{T}={\left[\begin{array}{ccc}\frac{1}{M}{\sum }_{j=1}^{M}\varDelta {T}_{1,j} & \cdots & \frac{1}{M}{\sum }_{j=1}^{M}\varDelta {T}_{t,j}\end{array}\right]}^{{{{\bf{T}}}}}$$

where M is the total number of temperature sensing points in the battery system. Owing to the structural differences between the battery modules used in laboratory experiments and the battery packs equipped in real-world vehicles, the optimal fitting parameters for the battery module and the battery pack are determined utilizing data collected during 1C charging–discharging process at 0 °C for the module and during the operation of “Vehicle 2,” respectively. The fitting results are shown in Supplementary Fig. 8. The parameters of the SE-waring boundary are summarized in Supplementary Table 4.

## Supplementary information

Supplementary Information

Transparent Peer Review file

## Source data

Source Data

## Data Availability

The experimental data generated in this study have been deposited in the Zenodo repository under DOI (https://doi.org/10.5281/zenodo.22121425)^55^. The numerical data underlying the figures are provided in the Source Data file. Source data are provided with this paper.

## Code availability

Code for this work is available at the following link: https://github.com/yuchao210/Multi-model-collaborative-early-warning-for-battery-systems-with-integrated-fiber-optic-sensors/tree/main.
