# Supplementary material for: Multi-model collaborative early warning for battery systems with integrated fiber optic sensors
Source: Nat Commun. 2026 Oct 9;17:10700. doi: 10.1038/s41467-026-78461-5 (PMC13658769; doi:10.1038/s41467-026-78461-5)
Supplement: Supplementary file 1 [file 41467_2026_78461_MOESM1_ESM.pdf]

Supplementary Information

Multi-model collaborative early warning for battery systems with integrated fiber optic sensors

Jiangong Zhu[1, 2, *], Chao Yu[1, 2], Yi Jiang[3], Wolfgang Schade[3,*], Martin Angelmahr[3], Xuezhe Wei[1, 2,*], Haifeng Dai[1, 2, *]

1 College of Automotive and Energy Engineering, Tongji University, Shanghai, 201804, China

2 Clean Energy Automotive Engineering Center, Tongji University, Shanghai, 201804, China

3 Optical Sensor Systems Department, Fraunhofer Heinrich Hertz Institute, Am Stollen 19H, 38640, Goslar, Germany

Supplementary Note 1. Cost analysis of the proposed warning scheme

The per-vehicle hardware cost of the proposed warning scheme is estimated according to the actual sensing configuration deployed in one battery pack. The system comprises 540 temperature sensing gratings, one onboard miniaturized interrogator, cables and accessories, and the materials required for sensor installation and fixation. The reference costs are derived from a supplier (Shanghai SensChain Co., Ltd.) quotation corresponding to a production scale of 10^4 vehicles. At this scale, the unit price of a temperature sensing grating is 0.12 USD per sensing point. Therefore, the cost of the 540 temperature sensing points installed in one battery pack is calculated as:

$$C_{FBG,10^4} = 540 \times 0.12 = 64.80 \text{ (USD/vehicle)}$$

The per-vehicle costs of the onboard miniaturized interrogator, cables and accessories, and installation materials are 35.50, 2.15, and 0.72 USD, respectively. Accordingly, the total hardware cost at the reference production scale is:

$$C_{total,10^4} = 64.80 + 35.50 + 2.15 + 0.72 = 103.17 \text{ (USD/vehicle)}$$

Furthermore, this study adopts a component-level technology learning-curve model to estimate the cost evolution of the proposed warning scheme with increasing deployment scale. This model is appropriate because the system mainly consists of standardized hardware components whose unit costs can decrease through cumulative manufacturing experience, process automation, yield improvement, and economies of scale [1]. Technology learning curves, which describe unit cost as a power-law function of cumulative production or deployment, have been widely used for energy and battery technology cost forecasting [2]. For the i-th hardware component, the unit cost at a cumulative deployment scale Q is expressed as:

$$C_{i,Q} = C_{i,Q_0}(Q/Q_0)^{\ln(1-LR_i)/\ln 2}$$

where $C_{i,Q}$ and C_{i,Q_0} are the per-vehicle cost of component i at deployment scale Q and Q_0, respectively. LR_i is the learning rate of component i.

Since the historical cost data across multiple production scales are not currently available for the proposed warning scheme, the learning rates adopted in this analysis are treated as scenario-based assumptions rather than parameters empirically identified from historical cost data. In its component-level technology cost projections, the International Energy Agency applies a learning rate of 10% to automotive battery cells and fuel cell stacks and a learning rate of 5% to battery packaging and fuel cell balance-of-plant components [3]. Based on these analogous component categories, a learning rate of 10% is assigned to the temperature sensing gratings and onboard miniaturized interrogator, while a learning rate of 5% is assigned to cables and accessories, and installation materials in this study. Building upon this, the projected per-vehicle costs at deployment scales of 10^4, 10^5, 10^6, and 10^7 vehicles are summarized below.

Deployment scales	Temperature sensing gratings	Interrogator	Cables and accessories	Installation materials	Total cost
10^4 vehicles	64.80 USD	35.50 USD	2.15 USD	0.72 USD	103.17 USD
10^5 vehicles	45.66 USD	25.02 USD	1.81 USD	0.61 USD	73.10 USD
10^6 vehicles	32.18 USD	17.63 USD	1.53 USD	0.51 USD	51.85 USD
10^7 vehicles	22.68 USD	12.42 USD	1.29 USD	0.43 USD	36.82 USD

Supplementary Note 2. Inconsistency analysis of battery module

In this study, the capacities of all LIBs used for assembling the two battery modules were calibrated following the standard procedures provided by the battery manufacturer. Each cell was first charged at a current of 0.5 C-rate to the upper cut-off voltage under constant current, followed by constant-voltage charging at 4.2 V until the current tapered to 0.02 C-rate. After resting for 1 h, the cells were discharged at a current of 0.2 C-rate to the lower cut-off voltage, and the measured discharge capacity was taken as the actual battery capacity. Based on these calibrated battery capacities, the module inconsistency was quantified using the normalized coefficient of variation (CV), as defined by the equation below [4]. The larger CV is, the more inconsistent the module is. As shown in the following figure, the CV values for Module 1 (used for overheating tests) and Module 2 (used for overcharge tests) are 0.28% and 0.23%, respectively, indicating minimal initial inconsistency across cells in both modules.

$$CV = \frac{1}{\bar{C}}\sqrt{\frac{1}{N}\sum_{i=1}^{N}(C_i - \bar{C})^2} \times 100\%$$

where $\bar{C}$ denotes the average battery capacity, N is the total number of batteries within a single module, and the subscript i denotes the battery index.

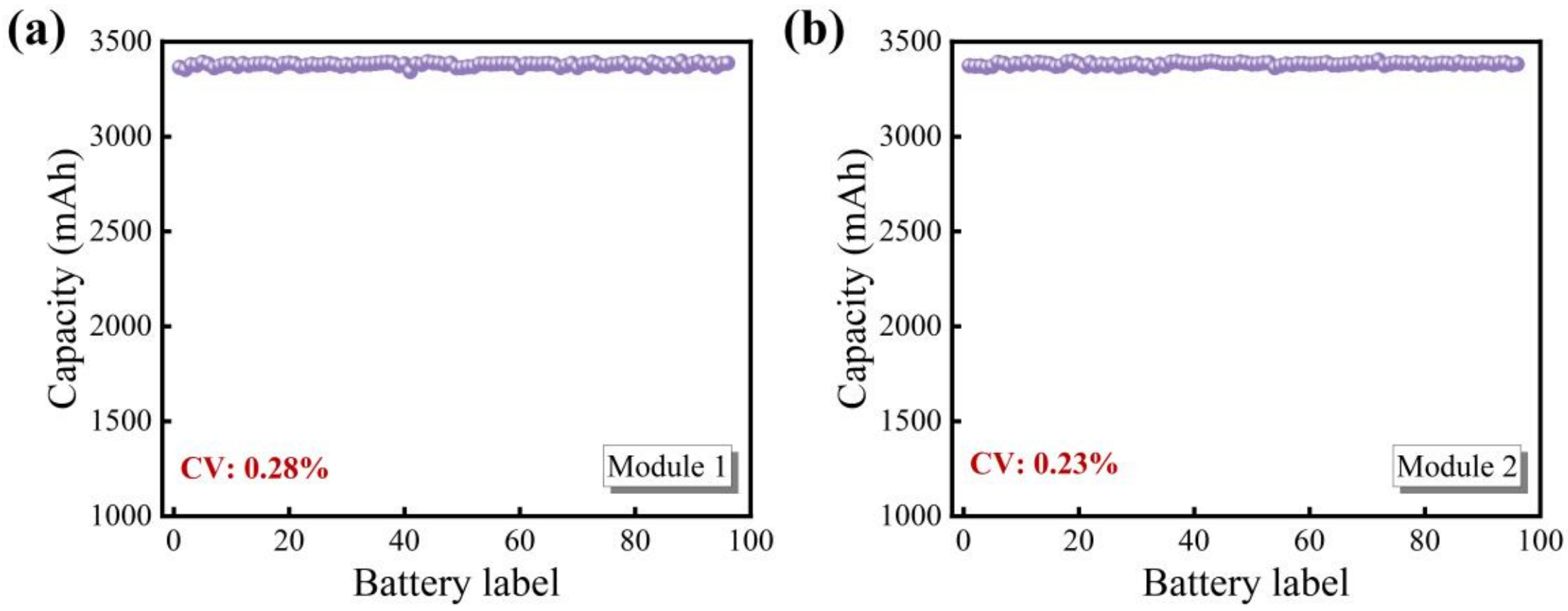

Supplementary Note 3. Temperature information of the EV battery pack integrated with fiber optic sensors

In real-vehicle scenarios, the structure of the distributed temperature data recorded by the fiber optic sensor interrogator is shown in panel (a) below. The first column corresponds to the timestamp recorded by the interrogator, with a non-uniform sampling frequency typically ranging from 0.5 to 1 Hz. The remaining columns correspond to temperature measurements at various sensing locations within the battery pack. For the column titles, the number between "T" and "_" denotes the index of the fiber optic sensor: sensors with odd indices are installed on the module sidewalls, whereas those with even indices are installed at the module tab regions. All fiber optic sensors are installed sequentially in ascending order of module index (as illustrated in panel (b) below). The number following "_" in each column title corresponds to the index of the fiber Bragg grating (FBG) point along the fiber. For the fiber optic sensors installed on Modules 1-14 and 16-17, the FBG indices increase sequentially from right to left, whereas for those installed on Modules 15 and 18, the indices increase from bottom to top, as illustrated in panel (b) below.

(a)

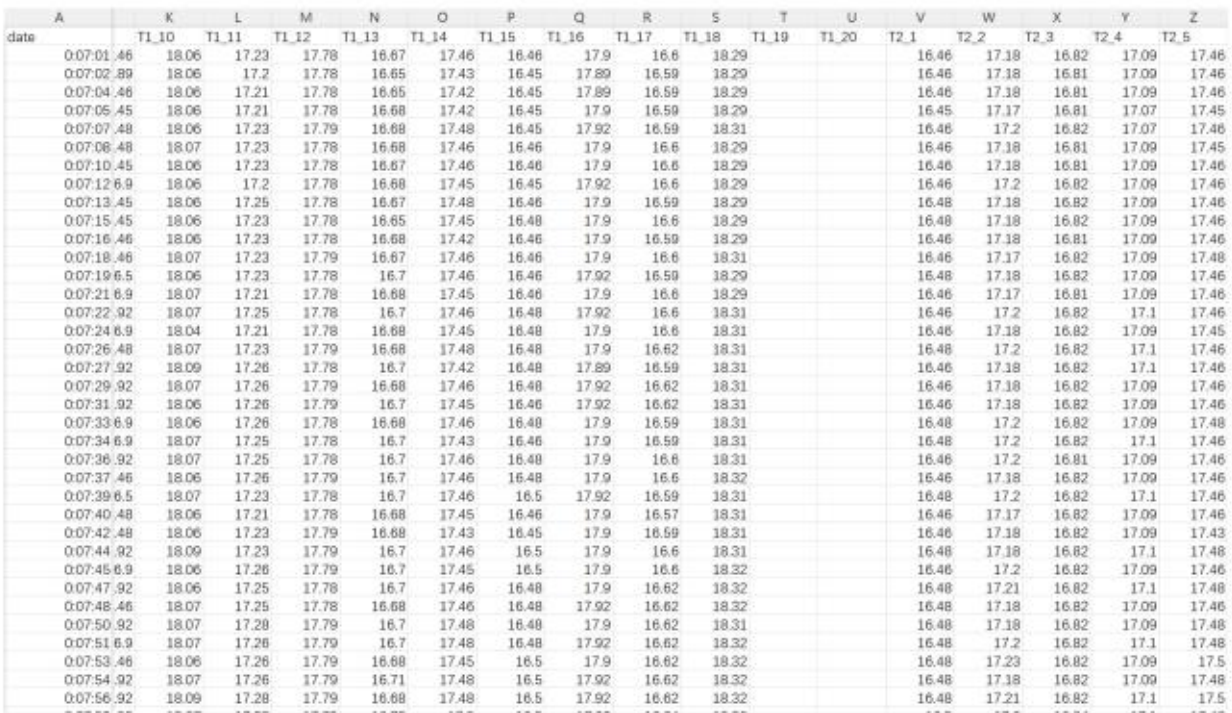

A	K	L	M	N	O	P	Q	R	S	T	U	V	W	X	Y	Z
date	T1_10	T1_11	T1_12	T1_13	T1_14	T1_15	T1_16	T1_17	T1_18	T1_19	T1_20	T2_1	T2_2	T2_3	T2_4	T2_5
0:07:01 .46	18.06	17.23	17.78	16.67	17.46	16.46	17.9	16.6	18.29			16.46	17.18	16.82	17.09	17.46
0:07:02 .89	18.06	17.2	17.78	16.65	17.43	16.45	17.89	16.59	18.29			16.46	17.18	16.81	17.09	17.46
0:07:04 .46	18.06	17.21	17.78	16.65	17.42	16.45	17.89	16.59	18.29			16.46	17.18	16.81	17.09	17.46
0:07:05 .45	18.06	17.21	17.78	16.68	17.42	16.45	17.9	16.59	18.29			16.45	17.17	16.81	17.07	17.45
0:07:07 .48	18.06	17.23	17.79	16.68	17.48	16.45	17.92	16.59	18.31			16.46	17.2	16.82	17.07	17.46
0:07:08 .48	18.07	17.23	17.78	16.68	17.46	16.46	17.9	16.6	18.29			16.46	17.18	16.81	17.09	17.45
0:07:10 .45	18.06	17.23	17.78	16.67	17.46	16.46	17.9	16.6	18.29			16.46	17.18	16.81	17.09	17.46
0:07:12 6.9	18.06	17.2	17.78	16.68	17.45	16.45	17.92	16.6	18.29			16.46	17.2	16.82	17.09	17.46
0:07:13 .45	18.06	17.25	17.78	16.67	17.48	16.46	17.9	16.59	18.29			16.48	17.18	16.82	17.09	17.46
0:07:15 .45	18.06	17.23	17.78	16.65	17.45	16.48	17.9	16.6	18.29			16.48	17.18	16.82	17.09	17.46
0:07:16 .46	18.06	17.23	17.78	16.68	17.42	16.46	17.9	16.59	18.29			16.46	17.18	16.81	17.09	17.46
0:07:18 .46	18.07	17.23	17.79	16.67	17.46	16.46	17.9	16.6	18.31			16.46	17.17	16.82	17.09	17.48
0:07:19 6.5	18.06	17.23	17.78	16.7	17.46	16.46	17.92	16.59	18.29			16.48	17.18	16.82	17.09	17.46
0:07:21 6.9	18.07	17.21	17.78	16.68	17.45	16.46	17.9	16.6	18.29			16.46	17.17	16.81	17.09	17.46
0:07:22 .92	18.07	17.25	17.78	16.7	17.46	16.48	17.92	16.6	18.31			16.46	17.2	16.82	17.1	17.46
0:07:24 6.9	18.04	17.21	17.78	16.68	17.45	16.48	17.9	16.6	18.31			16.46	17.18	16.82	17.09	17.45
0:07:26 .48	18.07	17.23	17.79	16.68	17.48	16.48	17.9	16.62	18.31			16.48	17.2	16.82	17.1	17.46
0:07:27 .92	18.09	17.26	17.78	16.7	17.42	16.48	17.89	16.59	18.31			16.46	17.18	16.82	17.1	17.46
0:07:29 .92	18.07	17.26	17.79	16.68	17.46	16.48	17.92	16.62	18.31			16.46	17.18	16.82	17.09	17.46
0:07:31 .92	18.06	17.26	17.79	16.7	17.45	16.46	17.92	16.62	18.31			16.46	17.18	16.82	17.09	17.46
0:07:33 6.9	18.06	17.26	17.78	16.68	17.46	16.48	17.9	16.59	18.31			16.48	17.2	16.82	17.09	17.48
0:07:34 6.9	18.07	17.25	17.78	16.7	17.43	16.46	17.9	16.59	18.31			16.48	17.2	16.82	17.1	17.46
0:07:36 .92	18.07	17.25	17.78	16.7	17.46	16.48	17.9	16.6	18.31			16.46	17.2	16.81	17.09	17.46
0:07:37 .46	18.06	17.26	17.79	16.7	17.46	16.48	17.9	16.6	18.32			16.46	17.18	16.82	17.09	17.46
0:07:39 6.5	18.07	17.23	17.78	16.7	17.46	16.5	17.92	16.59	18.31			16.48	17.2	16.82	17.1	17.46
0:07:40 .48	18.06	17.21	17.78	16.68	17.45	16.46	17.9	16.57	18.31			16.46	17.17	16.82	17.09	17.46
0:07:42 .48	18.06	17.23	17.79	16.68	17.43	16.45	17.9	16.59	18.31			16.46	17.18	16.82	17.09	17.43
0:07:44 .92	18.09	17.23	17.79	16.7	17.46	16.5	17.9	16.6	18.31			16.48	17.18	16.82	17.1	17.48
0:07:45 6.9	18.06	17.26	17.79	16.7	17.45	16.5	17.9	16.6	18.32			16.46	17.2	16.82	17.09	17.46
0:07:47 .92	18.06	17.25	17.78	16.7	17.46	16.48	17.9	16.62	18.32			16.48	17.21	16.82	17.1	17.48
0:07:48 .46	18.07	17.25	17.78	16.68	17.46	16.48	17.92	16.62	18.32			16.48	17.18	16.82	17.09	17.46
0:07:50 .92	18.07	17.28	17.79	16.7	17.48	16.48	17.9	16.62	18.31			16.48	17.18	16.82	17.09	17.48
0:07:51 6.9	18.07	17.26	17.79	16.7	17.48	16.48	17.92	16.62	18.32			16.48	17.2	16.82	17.1	17.48
0:07:53 .46	18.06	17.26	17.79	16.68	17.45	16.5	17.9	16.62	18.32			16.48	17.23	16.82	17.09	17.5
0:07:54 .92	18.07	17.26	17.79	16.71	17.48	16.5	17.92	16.62	18.32			16.48	17.18	16.82	17.09	17.48
0:07:56 .92	18.09	17.28	17.79	16.68	17.48	16.5	17.92	16.62	18.32			16.48	17.21	16.82	17.1	17.5

(b)

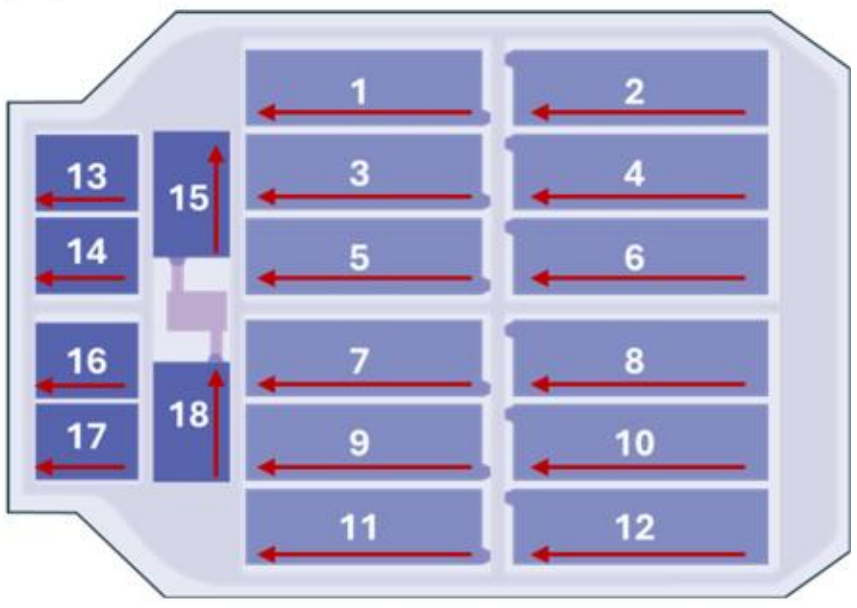

Supplementary Note 4. Details of the existing warning methods for performance comparison

Method 1: Temperature-based composite threshold strategy [5]

Let $T_{i,t}$ denote the temperature measured at the i-th sensing point at time t, and let Ω_t denote the set of temperature sensing points at that time. The maximum and minimum temperatures of the battery system are defined as

$$T_{max,t} = \max_{i\epsilon\Omega_t} T_{i,t} \tag{1}$$

$$T_{min,t} = \min_{i\epsilon\Omega_t} T_{i,t} \tag{2}$$

The instantaneous temperature range, used here to quantify temperature non-uniformity across the battery system, is calculated as

$$\Delta T_{range,t} = T_{max,t} - T_{min,t} \tag{3}$$

This metric captures the temperature contrast between the hottest and coolest sensing points. Unlike a spatial temperature gradient, it does not require information on the physical distances between sensing points and can therefore accommodate different sensor numbers and spatial arrangements at the module and pack levels. To characterize the temporal evolution of temperature, the temperature rise rate at the i-th sensing point is calculated from two consecutive measurements using the actual elapsed time:

$$\mathrm{R}_{i,t_k} = \frac{T_{i,t_k} - T_{i,t_{k-1}}}{t_k - t_{k-1}} \tag{4}$$

To suppress short-term measurement fluctuations, a moving average over W consecutive sampling points is applied:

$$\overline{\mathrm{R}}_{i,t_k} = \frac{1}{W} \sum_{j=k-W+1}^{k} \mathrm{R}_{i,t_j} \tag{5}$$

The maximum temperature rise rate of battery system is subsequently obtained from all valid sensing points:

$$\mathrm{R}_{max,t_k} = \max_{i\epsilon\Omega_{t_k}} \overline{\mathrm{R}}_{i,t_k} \tag{6}$$

Following the composite-threshold logic of the reference method and its adaptation to the present datasets, a thermal runaway warning is issued when the maximum temperature exceeds its prescribed threshold and either the maximum temperature rise rate or the temperature range exceeds the corresponding threshold:

$$T_{max,t} \geq T_{th} \wedge \left[\mathrm{R}_{max,t} \geq R_{th} \vee \Delta T_{range,t} \geq \Delta T_{th}\right] = 1 \tag{7}$$

where T_{th}, R_{th}, and ΔT_{th} denote the thresholds for the maximum temperature, maximum temperature rise rate, and temperature range, respectively. Based on the normal operating data collected at the lab-scale module level and at pack level under real-world vehicle operation, the parameters of this method are calibrated to suppress false warnings under normal operation. The final parameter settings adopted for the battery module and battery pack are summarized below.

**Parameters**	**Module-level**	**Pack-level**
Moving average window W	20	20
Maximum temperature threshold T_{th}	100 ℃	100 ℃
Maximum temperature rise rate threshold R_{th}	0.2 ℃/s	0.2 ℃/s
Temperature range threshold ΔT_{th}	20	20

Method 2: Temperature variance and LOI dual-threshold warning [6]

The temperature variance is employed to quantify the spatial non-uniformity of the distributed temperature field. At the t-th time step, the battery system contains M valid temperature sensing points, and the measured temperature at the j-th sensing point is denoted by $T_{t,j}$. The spatially averaged temperature is calculated as

$$\bar{T}_t = \frac{1}{M}\sum_{j=1}^{M} T_{t,j} \tag{8}$$

Accordingly, the temperature variance of the battery system is defined as

$$T_{var,t}^2 = \frac{1}{M}\sum_{j=1}^{M}\left(T_{t,j} - \bar{T}_t\right)^2 \tag{9}$$

where $T_{\mathrm{var},t}^2$ represents the dispersion of the distributed temperature measurements at the t-th time step. A larger temperature variance indicates a greater temperature difference among the sensing points and, consequently, a more non-uniform temperature field.

In addition to the numerical dispersion of the temperature field, the local overheating index (LOI) is introduced to characterize the spatial concentration of high-temperature sensing points. At each step, all valid temperature measurements are ranked in descending order, and the highest $\eta\%$ of the temperature sensing points are selected as the high-temperature subset. The sensing point with the maximum temperature is regarded as the hotspot center. Let $\left(x_{t,max}, y_{t,max}\right)$ denote the normalized spatial coordinates of the hotspot center and let $\left(x_{t,k}, y_{t,k}\right)$ denote the normalized coordinates of the k-th sensing point in the high-temperature subset. The LOi is calculated as

$$LOI_t = \frac{1}{N_h}\sum_{k=1}^{N_h}\sqrt{\left(x_{t,k} - x_{t,max}\right)^2 + \left(y_{t,k} - y_{t,max}\right)^2} \tag{10}$$

where N_h is the number of sensing points included in the high temperature subset. The spatial coordinates are normalized along each direction before calculation to eliminate the influence of the physical dimensions of the battery system. A smaller LOI value indicates that the high temperature sensing points are more strongly concentrated around the hotspot center, whereas a larger LOI value indicates a more dispersed high-temperature distribution.

Temperature variance and LOI characterize two complementary features of thermal anomalies. The temperature variance reflects the overall non-uniformity of the distributed temperature field, whereas the LOI describes the spatial concentration of the high temperature region. Therefore, a thermal anomaly is identified only when a significant increase in temperature non-uniformity is accompanied by pronounced local hotspot concentration. The instantaneous warning state at the t-th time step is defined as

$$A_t = \begin{cases} 1, & T_{var,t}^2 \geq T_{var,th}^2 \ and \ LOI_t \leq LOI_{th} \\ 0, & otherwise \end{cases} \tag{11}$$

where $T_{\mathrm{var,th}}^2$ is the upper warning threshold for temperature variance and $\mathrm{LOI_{th}}$ is the lower warning threshold for the local overheating index. Based on the normal operating data collected at the lab-scale module level and at pack level under real-world vehicle operation, the parameters of this method are calibrated to suppress false warnings under normal operation. The final parameter settings adopted for the battery module and battery pack are summarized below.

**Parameters**	**Module-level**	**Pack-level**

Upper threshold of temperature variance $T_{var,th}^2$	50	50
Lower threshold of local overheating index LOI_{th}	0.2	0.2
Proportion of high-temperature sensing points η	10%	10%

Method 3: Modified standardized CUSUM method [7]

For the temperature signal $T_{i,t}$ measured by the i-th sensing point at time t, an adaptive mean is first constructed using an exponentially weighted moving average:

$$\mu_{i,t} = (1-\beta)\mu_{i,t-1} + \beta T_{i,t} \tag{12}$$

where $\mu_{i,t}$ is the adaptive mean temperature of the i-th sensing point at time t, and β is the mean updating coefficient. Subsequently, the sample standard deviation of the temperature fluctuations is calculated using a sliding window with a length of W:

$$\sigma_{i,t} = \sqrt{\frac{1}{W-1}\sum_{j=t-W+1}^{t}\left(T_{i,j} - \bar{T}_{i,t}\right)^2} \tag{13}$$

where $\bar{T}_{i,t}$ denotes the average temperature of the i-th sensing point within the current sliding window. Under some operating conditions, the temperature fluctuation may be extremely small, and direct standardization may excessively amplify minor disturbances. Therefore, a lower bound $\sigma_{\min}$ is introduced for the standard deviation, and the standardized temperature residual is calculated as:

$$z_{i,t} = \frac{T_{i,t} - \mu_{i,t}}{\max\left(\sigma_{i,t}, \sigma_{min}\right)} \tag{14}$$

where $z_{i,t}$ represents the deviation of the current temperature from its recent normal operating state. Based on the standardized residual, the positive CUSUM statistics are recursively updated as:

$$C_{i,t}^{+} = \max\left[0, C_{i,t-1}^{+} + z_{i,t} - k\right] \tag{15}$$

where $C_{i,t}^{+}$ is the positive CUSUM statistic of the i-th temperature sensing point at time t, and k is the reference drift parameter used to suppress the accumulation of minor positive temperature deviations during normal operation. When persistent abnormal heating occurs at a sensing point, its corresponding $C_{i,t}^{+}$ continuously increases. In contrast, when the temperature fluctuation remains within the normal range, the CUSUM statistics remain at a low level or returns to zero.

For a battery module or battery pack containing M valid temperature sensing points, the system-level temperature warning statistic is defined as:

$$C_{sys,t} = \max_{i=1,2,\cdots,M} C_{i,t}^{+} \tag{16}$$

A thermal runaway warning is triggered when the positive CUSUM statistic of any valid temperature sensing point reaches the predefined threshold H:

$$C_{sys,t} \geq H \tag{17}$$

Based on the normal operating data collected at the lab-scale module level and at pack level under real-world vehicle operation, the parameters of the modified standardized CUSUM method are calibrated to suppress false warnings under normal operation. The final parameter settings adopted for the battery module and battery pack are summarized below.

**Parameters**	**Module-level**	**Pack-level**
Mean updating coefficient β	0.30	0.30
Length of sliding window W	20	20
Minimum standard deviation σ_{min}	0.05 ℃	0.05 ℃

Reference drift parameter k	0.50	0.50
Warning threshold H	7.00	30.00

Supplementary Tables

Supplementary Table 1. Specifications of 18650 cylindrical lithium-ion batteries.

**Items**	**Parameters**
Battery model	Samsung INR18650-35E
Cathode material	NCM
Anode material	Graphite
Nominal capacity	3.35 Ah
Nominal voltage	3.6 V
Cut-off voltage	2.65 V~4.2 V
Battery dimension	18.55 mm (diameter) × 65.25 mm (height)

Supplementary Table 2. Detailed experimental procedures for the battery cells presented in Fig. 3(b)

Cell label	Experimental items	Experimental details
#1	Overheating induced by a heating rod	The initial SOC of the battery is 0%, and the heating power is 80 W.
#2	Overheating induced by a heating rod	The initial SOC of the battery is 50%, and the heating power is 80 W.
#3	Overheating induced by a heating rod	The initial SOC of the battery is 100%, and the heating power is 80 W.
#4	Overheating induced by a heating rod	The initial SOC of the battery is 115%, and the heating power is 80 W.
#5	Overheating induced by a heating wire	The initial SOC of the battery is 0%, and the heating power is 80 W.
#6	Overheating induced by a heating wire	The initial SOC of the battery is 50%, and the heating power is 80 W.
#7	Overheating induced by a heating wire	The initial SOC of the battery is 100%, and the heating power is 80 W.
#8	Overheating induced by a heating wire	The initial SOC of the battery is 115%, and the heating power is 80 W.
#9	External short circuit	The initial SOC of the battery is 50%, and the short-circuit resistance is 20 mΩ.
#10	External short circuit	The initial SOC of the battery is 100%, and the short-circuit resistance is 20 mΩ.
#11	External short circuit	The initial SOC of the battery is 100%, and the short-circuit resistance is 50 mΩ.
#12	Overcharging	The initial SOC of the battery is 0%, and the charging current is 0.5 C.
#13	Overcharging	The initial SOC of the battery is 0%, and the charging current is 1.0 C.
#14	Overcharging	The initial SOC of the battery is 0%, and the charging current is 2.0 C.
#15	Overdischarging	The initial SOC of the battery is 100%, and the discharging current is 0.5 C.
#16	Overdischarging	The initial SOC of the battery is 100%, and the discharging current is 1.0 C.
#17	Overdischarging	The initial SOC of the battery is 100%, and the discharging current is 2.0 C.
#18	Constant-current charging-discharging	The battery is charged and discharged at currents of 0.5 C, 1 C, 2 C and 4 C.

Supplementary Table 3. Parameter settings of the warning framework using sparse temperature data as input

	Temperature warning threshold	dT/dt warning threshold	SE warning boundary				dSE/dt warning threshold
			a	b	c	E_{tol}	
Module-level	100	1	-1.91	0.09	3.96	0.3	0.2
Pack-level	100	1	-1.00	0.55	2.53		0.2

Supplementary Table 4. Fitting parameters for the nonlinear relationship between temperature Shannon entropy and average temperature variation.

	**a**	**b**	**c**	**E_{tol}**
Module-level	-3.352	0.08545	4.418	0.3
Pack-level	-1.419	0.397	2.693	

Supplementary Figures

Supplementary Fig. 1. Evolution of battery surface temperature under various C-rates and current directions. (a) CC discharge at a current of 0.5 C; (b) CC charge at a current of 0.3 C; (c) CC discharge at a current of 0.3 C.

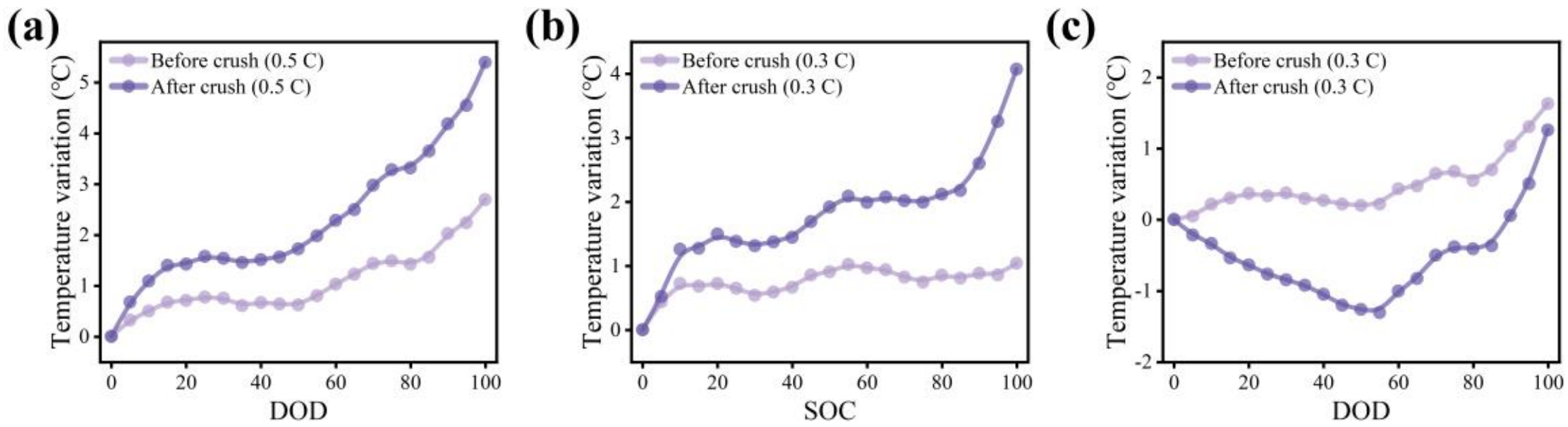

Supplementary Fig. 2. Parallel-cell voltage evolution of the battery modules and packs undergo thermal runaway. (a) During the module-level overcharging. (b) During the module-level overheating test. (c) On the day of thermal runaway in "Vehicle 1". (d) On the day of thermal runaway in "Vehicle 4"

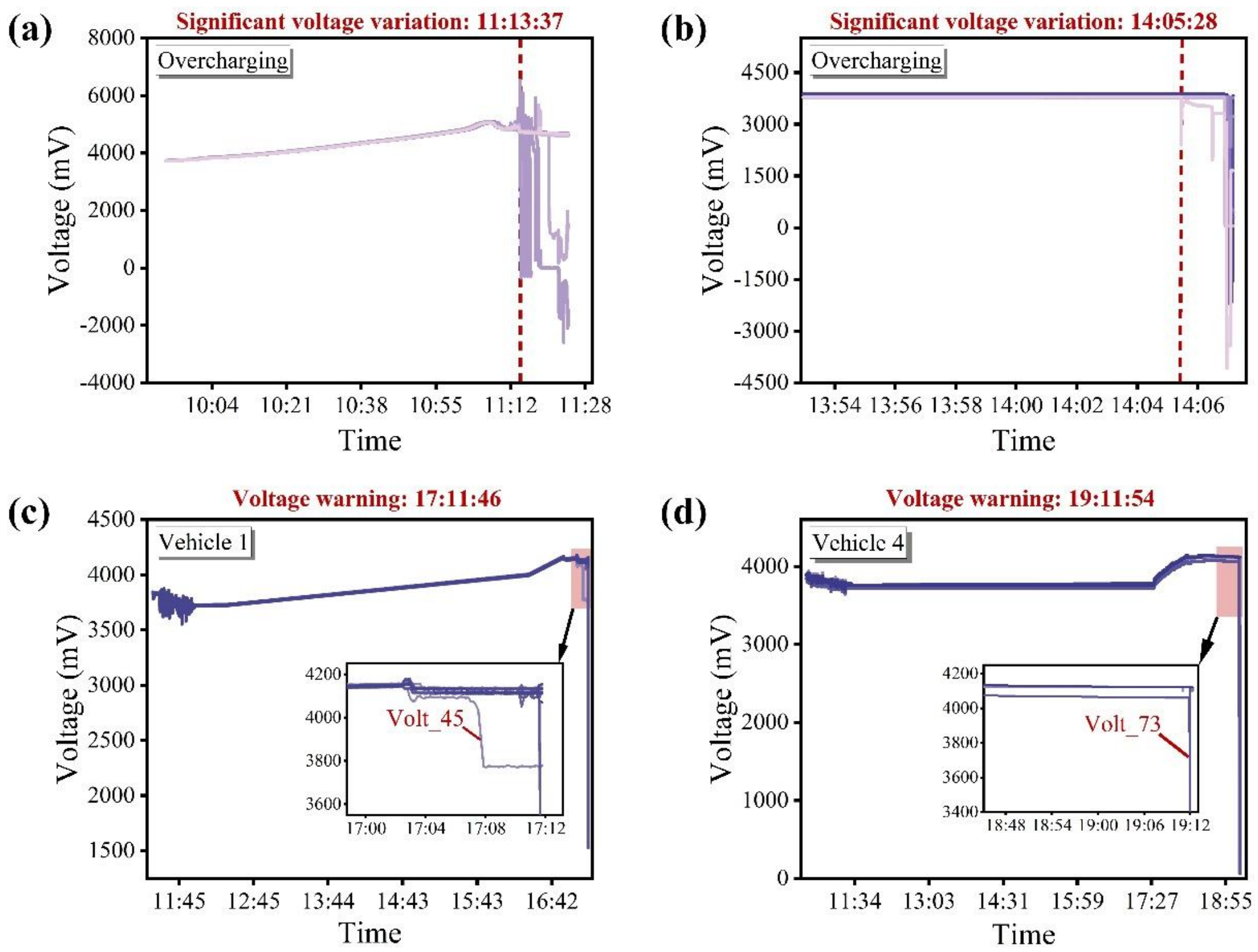

Supplementary Fig. 3. Comparison of the thermal response of the fiber optic sensor and thermocouple. The grating region of the fiber optic sensor and the thermocouple junction are secured together using high-temperature tape and rapidly transferred from an ambient environment at 25 °C into a thermal chamber maintained at 100 °C.

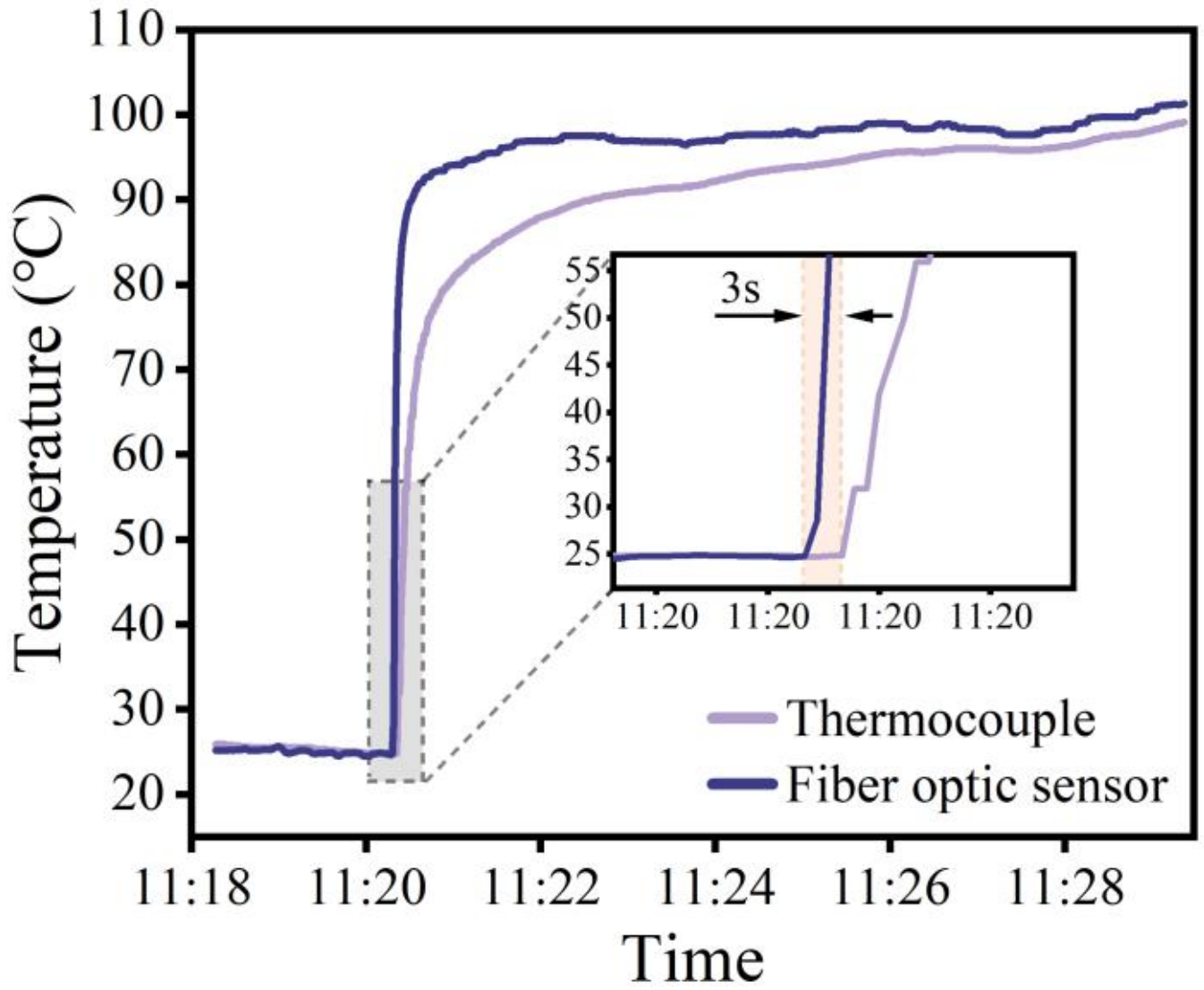

Supplementary Fig. 4. Experimental setups for cell-level testing. (a) Overheating-induced thermal runaway test setup using a heating rod. (b) Overheating-induced thermal runaway test setup using a heating wire. (c) ESC test setup. (d) ARC test setup. (e) Mechanical crush test setup. (f) Test setup for overcharging, overdischarging and constant-current charging-discharging tests. (Knife-switch and resistance icons in Supplementary Fig. 4c and waveform-monitor icon in Supplementary Fig. 4e: designed by Magnific from Flaticon.)

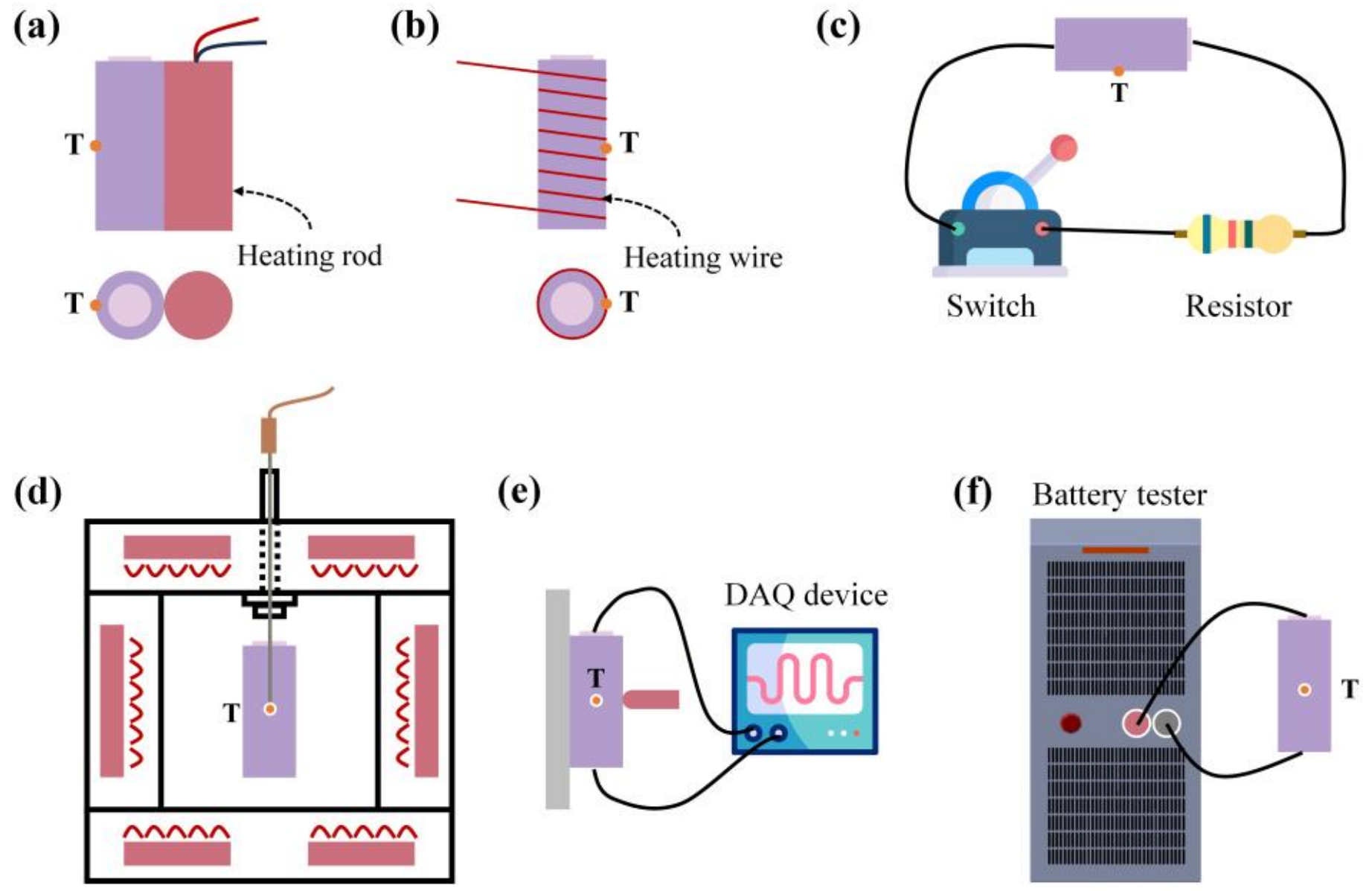

Supplementary Fig. 5. Calibration results of several fiber optic sensors employed in this study at various environmental temperatures.

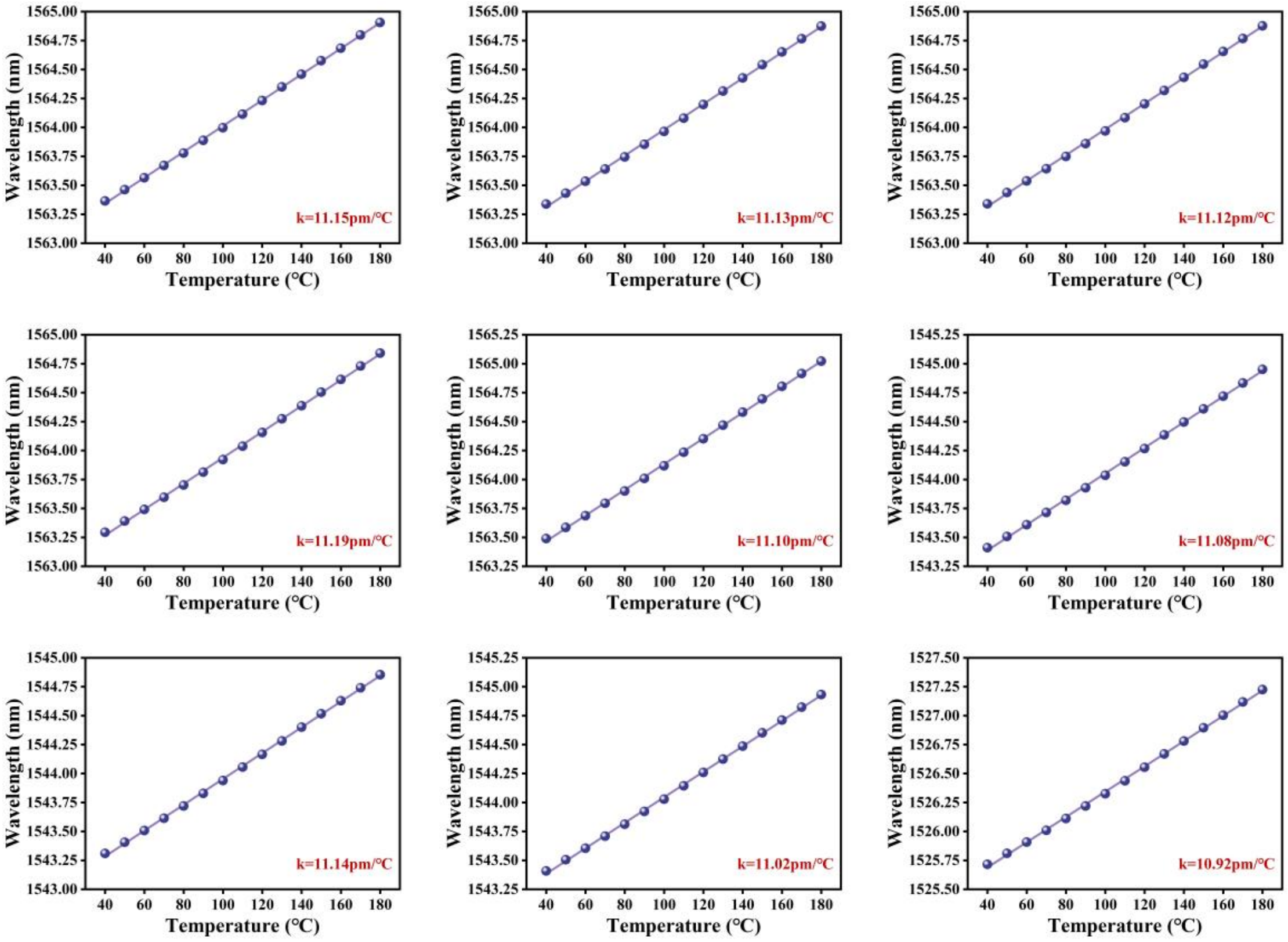

Supplementary Fig. 6. Dimensions of the miniaturized interrogator and schematic of its installation location within the battery pack.

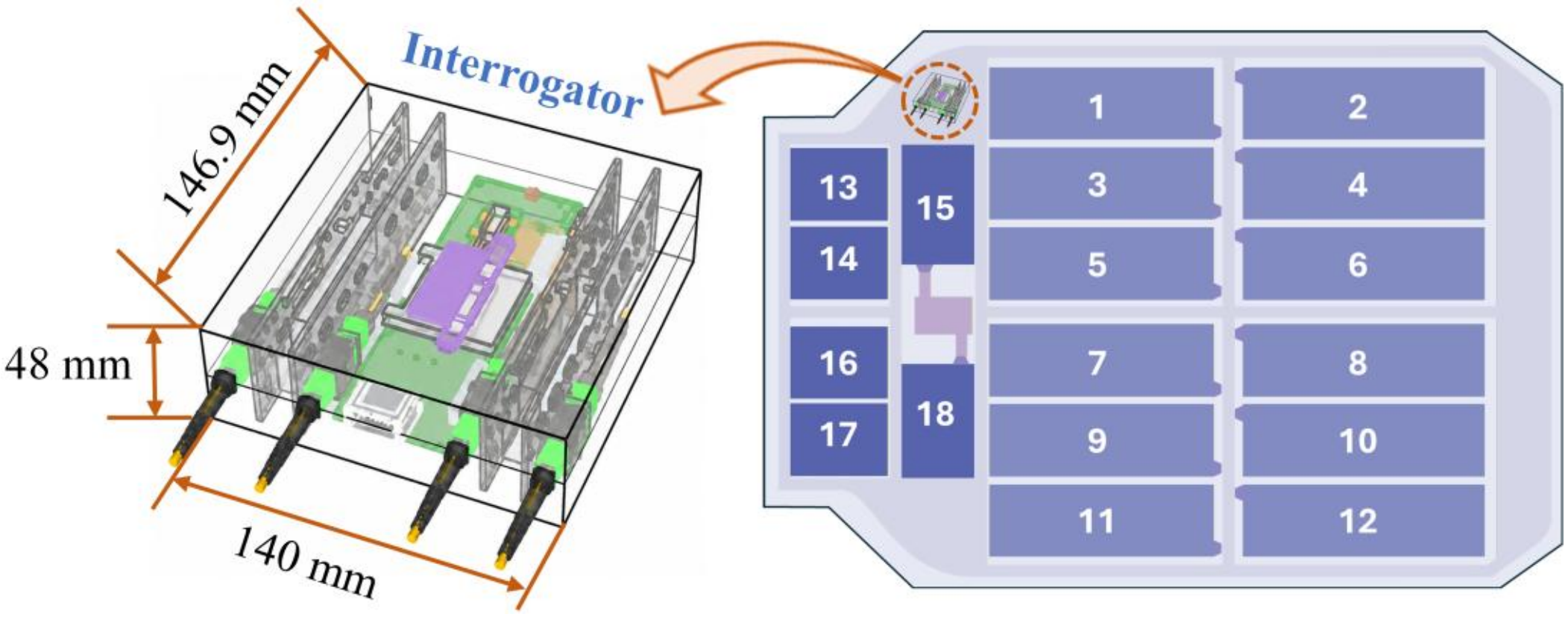

Supplementary Fig. 7. Comparison of temperature measurements obtained by fiber optic sensor and thermocouple during battery ARC testing.

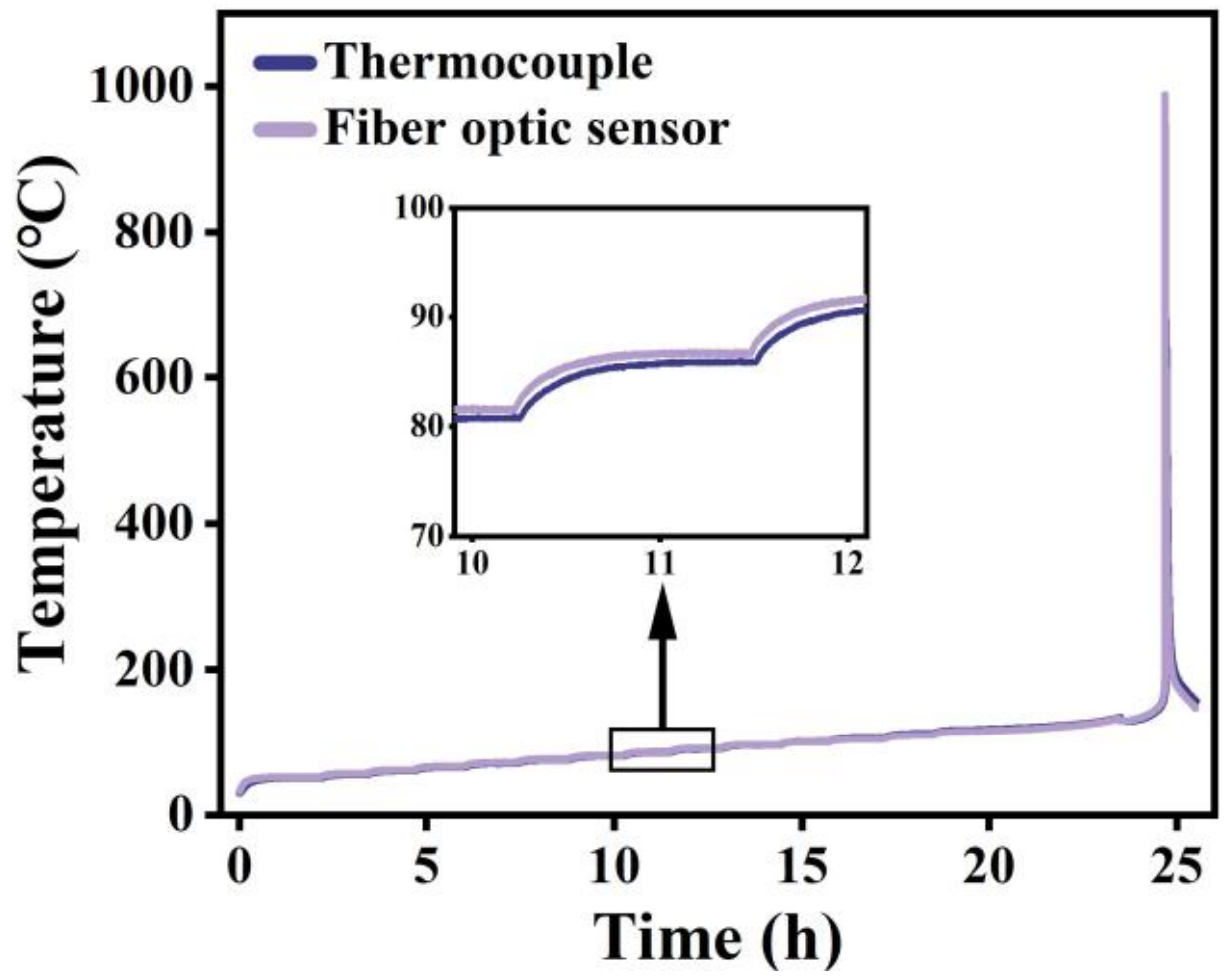

Supplementary Fig. 8. Fitting results for the nonlinear relationship between temperature Shannon entropy and the average temperature variation. The average temperature variation is binned at 0.5 °C intervals, and the maximum Shannon entropy within each interval, together with its corresponding average temperature variation, is selected for fitting data. (a) Module-level; (b) Pack-level.

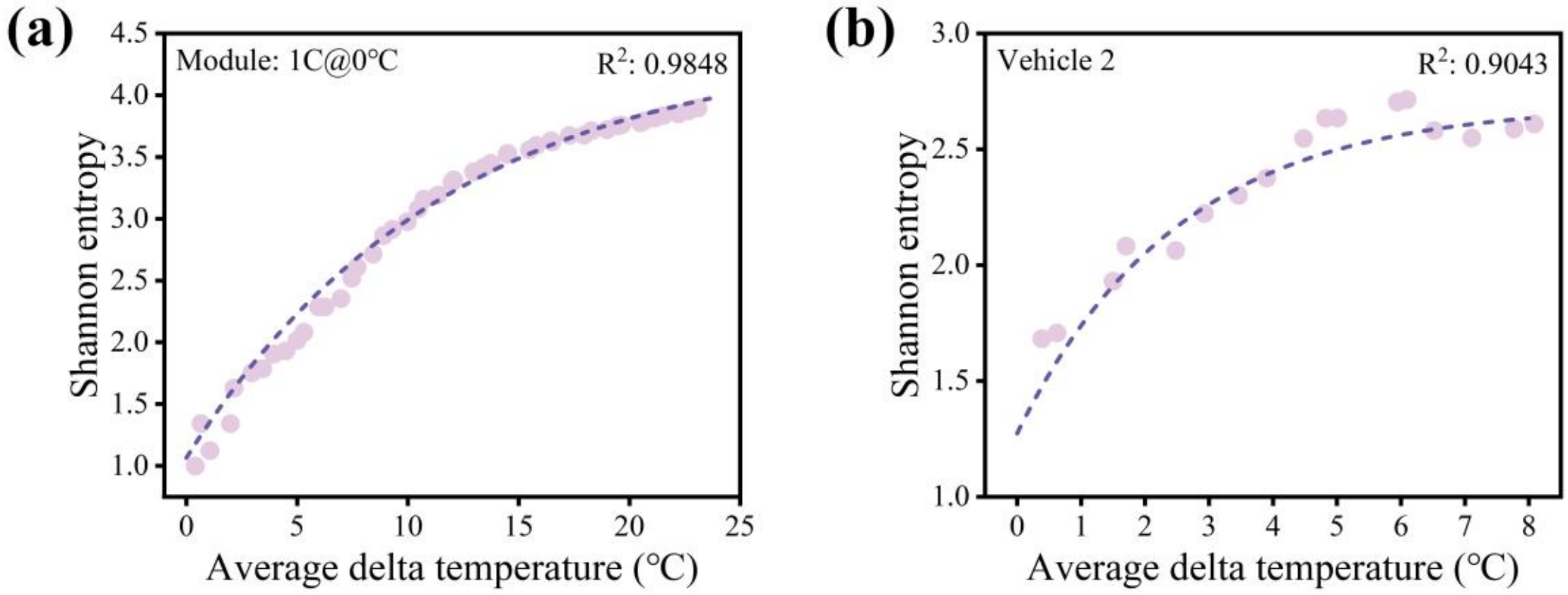

Supplementary References

1 Ziegler, M. S. & Trancik, J. E. Re-examining rates of lithium-ion battery technology improvement and cost decline. *Energy & Environmental Science* **14**, 1635-1651 (2021).

2 Wu, W., Zhai, H. & Holubnyak, E. Technological evolution of large-scale blue hydrogen production toward the U.S. Hydrogen Energy Earthshot. *Nat. Commun.* **15**, 5684 (2024).

3 *Unit cost reductions for selected technologies in the Sustainable Development Scenario, 2020–2070*, https://www.iea.org/data-and-statistics/charts/unit-cost-reductions-for-selected-technologies-in-the-sustainable-development-scenario-2020-2070

4 Wang, X., Fang, Q., Dai, H., Chen, Q. & Wei, X. Investigation on Cell Performance and Inconsistency Evolution of Series and Parallel Lithium-Ion Battery Modules. *Energy Technology* **9**, 2100072 (2021).

5 Yue, Y. et al. Early warning of thermal runaway in large-capacity LiFePO4 batteries based on distributed optical fiber. J. Energy Storage 141, 119280 (2026).

6 Wang, S. et al. Infrared imaging investigation of temperature fluctuation and spatial distribution for a large laminated lithium–ion power battery. Applied Thermal Engineering 152, 204-214 (2019).

7 Vu, L. A. et al. A real-time framework for early detection and severity prediction of thermal runaway in Li-ion batteries. J. Energy Storage 135, 118310 (2025).
