# Supplementary material for: Multi-model collaborative early warning for battery systems with integrated fiber optic sensors
Source: Nat Commun. 2026 Oct 9;17:10700. doi: 10.1038/s41467-026-78461-5 (PMC13658769; doi:10.1038/s41467-026-78461-5)
Supplement: Supplementary file 2 [file 41467_2026_78461_MOESM2_ESM.pdf]

nature portfolio

Peer Review File

Multi-model collaborative early warning for battery systems with integrated fiber optic sensors

Corresponding Author: Professor Jiangong Zhu

Parts of this Peer Review File have been redacted as indicated to remove third-party material.

This file contains all reviewer reports in order by version, followed by all author rebuttals in order by version.

Version 1:

Reviewer comments:

Reviewer #1

(Remarks to the Author)
This study puts forward an innovative methodology for proactive early warning of battery thermal runaway. By fusing high-fidelity, high-density internal temperature data of the battery system into a novel multi-model collaborative warning architecture, this work achieves remarkable, substantial breakthroughs in boosting the sensitivity, timeliness and reliability of thermal runaway pre-alarm capabilities. A standout, pioneering contribution of this research lies in the full-scale experimental verification of the integrated fiber-optic sensing layout under authentic dynamic vehicle operational scenarios, which enables precise capture of spatially dense temperature distribution across the entire battery pack. This rigorous real-vehicle validation solidly corroborates the excellent engineering practicability and scalable deployment value of the devised sensing integration strategy; more importantly, it establishes a uniquely valuable, practically measured dataset to underpin the optimization and iteration of next-generation high-performance battery management system (BMS) control strategies. Given the prominent novelty, solid experimental foundation and evident industrial translational significance of this work, the manuscript stands strong potential for acceptance in Nature Communications provided the authors thoroughly resolve the following remaining concerns.
Fig. 1(a) shows that the real-world vehicle operation lasted for “>1300 days”, whereas the main text describes the operation period as ranging from several months to two years. The authors should doublecheck that the figure and the text are consistent.
The paper doesn't give enough details about the fiber optic sensor interrogator. For example, they don't mention its size or where it's installed inside the battery pack. In my opinion, the size of the interrogator is a key factor in whether this technology can be used in real vehicles. Therefore, the authors should provide more information about the interrogator and how it's integrated into the battery system.
The authors claim that their warning scheme is suitable for realvehicle deployment. But the manuscript doesn't talk about the hardware needed to store all that highdensity temperature data inside the battery system and to run the warning algorithms.
The Shannon-entropy-based warning strategy is mainly used during the temperature rise phases of the battery system. However, the paper doesn't clearly explain how to determine that the battery system is in a temperature rise state. The authors should add a detailed description of how to identify that, which would make it easier for others to reproduce their method.
The paper proposes a "multi-model collaborative" framework that incorporates a temperature threshold, a temperature change rate threshold, a Shannon entropy change rate threshold. However, the description of the specific decision logic governing the "collaboration" among these models is vague. It is suggested that the final decision logic for the multi-model collaborative early warning be explicitly clarified, for instance, in the form of a flowchart or formalized conditional statements.
It is unclear whether the calibration of the parameters for this boundary model is a "one-time" procedure. In other words, for a battery system of a specific model/configuration, is calibration required only once in its fresh state? Alternatively, does this relationship change with battery aging, necessitating periodic re-calibration?
Using a voltage variation rate of 1 V/s for the parallel-cell voltage as the reference criterion for fault warning in the conventional BMS does not fully represent the existing, more sophisticated warning strategies in the industry. To more fairly and convincingly demonstrate the advancement of the proposed framework, it is recommended to add comparisons with more comprehensive conventional warning strategies.
The statement that "the per-vehicle application cost of the proposed warning scheme is only approximately 40 USD" would

benefit from a brief cost breakdown analysis in the Discussion or Supplementary Materials. Clarifying how this figure is derived and whether it includes the cost of the interrogator (in addition to the fiber optic sensors) would significantly strengthen the persuasiveness of the commercialization potential of the proposed scheme.

(Remarks on code availability)

Reviewer #2

(Remarks to the Author)
This paper proposes a multi-model collaborative thermal runaway early warning framework based on distributed temperature sensing, aiming to address two well-defined limitations inherent to conventional BMS: sparse temperature information and monolithic diagnostic frameworks. The authors exploit fiber optic sensors to achieve high-density temperature sensing throughout the battery system with minimal wiring complexity, and the long-term field validation under real-world vehicle scenarios demonstrates the potential engineering applicability of the proposed scheme. Furthermore, the established functional relationship between temperature Shannon entropy and mean temperature variation is novel and insightful. Overall, the manuscript exhibits a well-organized structure, maintains a clear application-oriented focus, and holds potential value for next-generation battery safety management. However, several concerns remain to be clarified before it can be accepted for publication.
1. In the final paragraph of the Introduction, the authors mention the cost of the proposed warning scheme. However, the cost structure of this scheme remains unclear. The authors are encouraged to provide a more detailed explanation of the main cost components so that they can better support the claimed cost-effectiveness of the proposed approach.
2. The authors fit the functional relationship between the temperature Shannon entropy and the mean temperature variation in the battery system using a customized equation. However, no goodness-of-fit metrics are reported in either the main text or the supplementary materials.
3. The authors only present the surface temperature differences during 0.5 C constant-current charging before and after mechanical crush in Fig. 3(c). Whether such differences persist under other lower current rates or during discharging?
4. The authors are advised to unify the terminology and address certain language-related details throughout the manuscript. For instance, both "fiber optic sensors" and "fiber-optic sensors" appear in the text. Although these issues do not significantly affect the content, consistent use of terminology would enhance the overall readability and professionalism of the paper.
5. The warning lead times reported in the manuscript are only evaluated relative to the BMS warning strategy. The authors are encouraged to briefly discuss how these lead times compare with those reported for other advanced early-warning methods in the existing literature.
6. The advancement of the fiber sensor against conventional thermal couple need to be further elaborated. Does the proposed method with Shannon Entropy also suitable for conventional temperature or electric signals? Please also discuss the underlying mechanistic meaning to enhance the physical significance of the feature, which will improve the scientific guidance in this field.
7. More comparative results against existing thermal runaway warning methods are required to demonstrate the advantages of the proposed method.
8. More explanations on Fig. 1d are needed, how does the authors obtain the decreasing curve with the numbers need to be clarified.

(Remarks on code availability)
N/A

Version 2:

Reviewer comments:

Reviewer #1

(Remarks to the Author)
accept

(Remarks on code availability)

Reviewer #2

(Remarks to the Author)
All comments have been well addressed.

(Remarks on code availability)

Open Access This Peer Review File is licensed under a Creative Commons Attribution 4.0 International License, which permits use, sharing, adaptation, distribution and reproduction in any medium or format, as long as you give appropriate credit to the original author(s) and the source, provide a link to the Creative Commons license, and indicate if changes were made.
In cases where reviewers are anonymous, credit should be given to 'Anonymous Referee' and the source.
The images or other third party material in this Peer Review File are included in the article's Creative Commons license, unless indicated otherwise in a credit line to the material. If material is not included in the article's Creative Commons license and your intended use is not permitted by statutory regulation or exceeds the permitted use, you will need to obtain permission directly from the copyright holder.
To view a copy of this license, visit https://creativecommons.org/licenses/by/4.0/

Author's Responses

Manuscript Number: NCOMMS-26-010836A-Z

Title: Multi-model collaborative early warning for battery systems with integrated fiber optic sensors

Dear Editor and Reviewers:

We would like to express our sincere gratitude and appreciation to the editor and reviewers for evaluating our manuscript and providing many valuable comments and suggestions, which have helped us greatly to improve the presentation and quality of our manuscript. We have made our best efforts to carefully address all the valuable comments in the revised manuscript. The detailed responses and revisions have been made, as listed below and highlighted by the yellow marker in the revised manuscript. We sincerely hope the revision will meet with your approval. Again, thank you very much for your valuable comments and assistance.

Yours sincerely,

Jiangong Zhu, Ph.D.
Professor
College of Automotive and Energy Engineering,
Clean Energy Automotive Engineering Center,
Tongji University

Responses to Reviewer #1:

This study puts forward an innovative methodology for proactive early warning of battery thermal runaway. By fusing high-fidelity, high-density internal temperature data of the battery system into a novel multi-model collaborative warning architecture, this work achieves remarkable, substantial breakthroughs in boosting the sensitivity, timeliness and reliability of thermal runaway pre-alarm capabilities. A standout, pioneering contribution of this research lies in the full-scale experimental verification of the integrated fiber-optic sensing layout under authentic dynamic vehicle operational scenarios, which enables precise capture of spatially dense temperature distribution across the entire battery pack. This rigorous real-vehicle validation solidly corroborates the excellent engineering practicability and scalable deployment value of the devised sensing integration strategy; more importantly, it establishes a uniquely valuable, practically measured dataset to underpin the optimization and iteration of next-generation high-performance battery management system (BMS) control strategies. Given the prominent novelty, solid experimental foundation and evident industrial translational significance of this work, the manuscript stands strong potential for acceptance in Nature Communications provided the authors thoroughly resolve the following remaining concerns.

Response: We greatly appreciate your recognition of the novelty of the proposed distributed temperature sensing and multi-model collaborative thermal runaway warning framework, the significance of the full-scale real-vehicle validation, and the potential engineering and practical value of this work for next-generation battery safety management. Following your comments, we have carefully revised the manuscript and provided additional analyses and clarifications. Specifically, we have further added comparisons with existing thermal runaway warning methods to further benchmark the performance of the proposed framework; supplemented the physical dimensions and installation arrangement of the miniaturized interrogator within the battery pack; quantitatively evaluated the memory and computational requirements for real-time onboard implementation of the proposed warning framework; and further discussed the current limitations and future directions regarding model transferability and practical deployment. These revisions provide additional evidence for the sensing and warning performance of the proposed approach and further strengthen the methodological completeness and engineering relevance of the manuscript. We sincerely appreciate your insightful comments, which have helped us substantially improve the quality and completeness of the manuscript. All corresponding revisions have been highlighted in yellow in the revised manuscript, and detailed responses to each comment and the corresponding modifications are provided below.

Comment 1:

Fig. 1(a) shows that the real-world vehicle operation lasted for “>1300 days”, whereas the main text describes the operation period as ranging from several months to two years. The authors should doublecheck that the figure and the text are consistent.

Response: Thank you for pointing out the inconsistency between Fig. 1(a) and the description of the real-world vehicle operation periods in the main text. Upon rechecking the vehicle-operation records, we find that “>1300 days” in Fig. 1(a) is intended to represent the cumulative operating duration of all seven vehicles, whereas the corresponding statements in the main text refer to the operating duration of individual vehicles. Because the figure does not explicitly specify that this value represents a cumulative duration, the original presentation may be misinterpreted as indicating an operating period of more than 1,300 days for an individual vehicle. We further recheck the data presented in Fig. 2(f). The individual monitoring periods of the seven vehicles range from 25 to 410 days, corresponding to a cumulative duration of 1,284 vehicle-days. Therefore, the original expressions “up to two years” and “several months to two years” are inaccurate and are corrected accordingly.
Following your suggestion, “>1300 days” in Fig. 1(a) is replaced with “1,284 vehicle-days.” The Abstract, Introduction, and Discussion are also revised to consistently state that the seven vehicles are individually monitored for 25–410 days, with a cumulative monitoring duration of 1284 vehicle-days. All corresponding modifications are highlighted in yellow in the revised manuscript, and representative changes are shown below. We again appreciate your careful examination, which eliminates the ambiguity between the individual and cumulative operating durations and improves the accuracy and consistency of the manuscript.

In Introduction:

More critically, the fiber optic sensors maintained stable operation across seven EVs over individual monitoring periods ranging from 25 to 410 days, corresponding to a cumulative duration of 1284 vehicle-days, thereby confirming the engineering feasibility of the integration scheme developed in this study.

In Discussion:

The designed scheme for integrating fiber optic sensors into battery systems is validated in real-world vehicle operation for the first time, with seven instrumented EVs operating over periods of up to two years, corresponding to a cumulative duration of 1284 vehicle-days, confirming their robustness and engineering readiness for practical battery system deployment.

In Fig. 1:

[REDACTED]

Comment 2:

The paper doesn't give enough details about the fiber optic sensor interrogator. For example, they don't mention its size or where it's installed inside the battery pack. In my opinion, the size of the interrogator is a key factor in whether this technology can be used in real vehicles. Therefore, the authors should provide more information about the interrogator and how it's integrated into the battery system.

Response: Thank you for this important comment. We agree that the physical dimensions and installation arrangement of the fiber optic interrogator are important considerations for evaluating the feasibility of integrating the proposed sensing system into a real vehicle battery pack. Following your suggestion, we add the dimensions of the miniaturized interrogator and a schematic of its installation location within the battery pack in Supplementary Fig. 6. The interrogator used in this study has overall dimensions of approximately 146.9 mm × 140 mm × 48 mm, and its integration position within the battery pack is explicitly illustrated in the supplementary figure. A corresponding cross-reference is also added to the Methods section. These additional details provide a clearer description of the hardware configuration and physical integration of the interrogator, and further clarify the practical feasibility of deploying the proposed fiber optic sensing scheme in a vehicle battery system. All corresponding modifications are highlighted in yellow in the revised manuscript and are shown below.

In Method:

The dimensions of the interrogator and its installation location within the battery pack are illustrated in **Supplementary Fig. 6**.

In Supplementary Information

Supplementary Fig. 6. Dimensions of the miniaturized interrogator and schematic of its installation location within the battery pack.

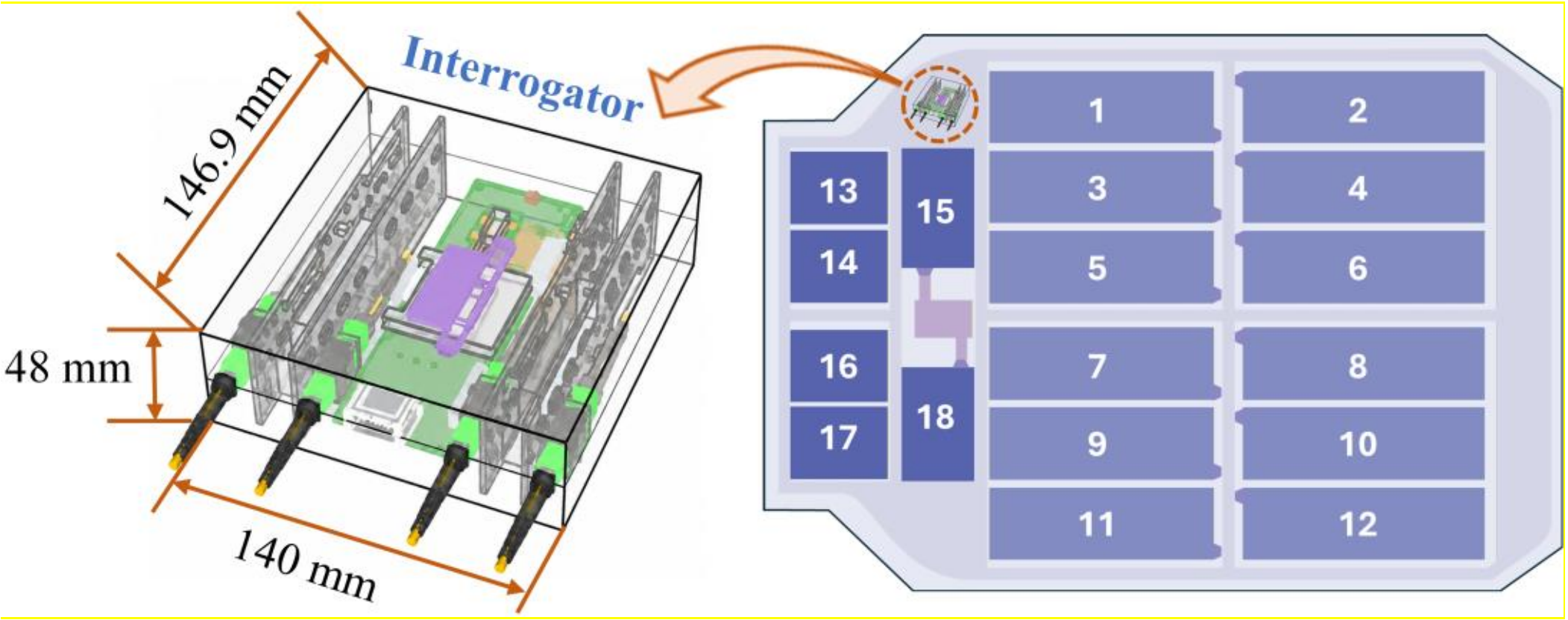

Comment 3:

The authors claim that their warning scheme is suitable for real vehicle deployment. But the manuscript doesn't talk about the hardware needed to store all that high density temperature data inside the battery system and to run the warning algorithms.

Response: Thank you for raising this important concern regarding the hardware requirements for real-vehicle deployment. We agree that the original manuscript does not sufficiently discuss the memory and computational resources required to process the high-density temperature measurements onboard. Following your suggestion, we add a new subsection entitled "Onboard implementation feasibility" in the Results section to quantitatively evaluate the hardware requirements of the proposed warning framework. For the pack-level configuration with 540 temperature sensing points and a maximum sliding window of 500 samples, approximately 1.08 MB of memory is required to buffer the temperature data using 32-bit floating-point representation. After further considering reference temperatures, intermediate variables, communication buffers, and the software stack, approximately 2 MB of on-chip SRAM is estimated to be sufficient for real-time implementation. We also quantify the computational demand of the warning framework. The main calculations include linear least-squares fitting, threshold comparisons, and Shannon entropy evaluation. Under conservative processor-cycle assumptions, one complete warning update requires approximately processor clock cycles. After introducing a tenfold engineering margin for communication, diagnostics, operating-system tasks, and other BMS functions, the computational budget remains approximately processor clock cycles per 1-Hz update, corresponding to a processing capability on the order of 10 MHz. These estimates indicate that the proposed framework can be implemented using currently available automotive-grade real-time processors without requiring a dedicated high-performance computing platform. The corresponding analysis has been added to the revised manuscript and highlighted in yellow, as shown below.

In Results:

Onboard implementation feasibility

The onboard resource requirements of the proposed warning framework are further evaluated in terms of memory usage and computational demand in this study. Specifically, the pack-level sensing scheme developed in this study contains 540 temperature sensing points, and the longest sliding window employed in the warning framework contains 500 samples. With 32-bit floating-point representation, the corresponding temperature data buffer occupies 1.08 MB. After further accounting for reference temperatures, intermediate variables, communication buffers, and the software stack, an on-chip static random-access memory (SRAM) capacity of 2 MB represents a reasonable hardware target for real-time implementation. In terms of computational demand, the proposed framework primarily involves linear least-squares fitting, threshold comparisons, and Shannon entropy calculation. In accordance with the number of temperature sensing points and the corresponding window lengths, each 1 Hz update requires approximately basic arithmetic and comparison operations. Under a conservative upper-bound assumption, the Shannon entropy calculation additionally involves at most 540 logarithmic evaluations, while evaluation of the dynamic entropy boundary requires only one exponential operation. Using conservative processor-cycle assumptions, the computational demand of one complete warning update is estimated to be on the order of 10^6 processor clock cycles. After introducing a tenfold engineering margin for communication, diagnostics, operating-system tasks, and other BMS functions, the resulting computational budget remains approximately 10^7 processor clock cycles per 1 Hz update. Accordingly, an automotive-grade real-time processor with at least 2 MB of on-chip SRAM and a clock frequency on the order of 10 MHz or higher provides substantial hardware margin for onboard implementation of the proposed warning framework. Such requirements are within the capabilities of commercially available automotive-grade processors [53,54]. In future work, the proposed warning algorithm will be further implemented on automotive-grade hardware, and its real-time execution performance will be further evaluated under real-world vehicle operating conditions.

In Reference:

53 Texas Instruments. AM2634-Q1 automotive 400-MHz quad-core Arm Cortex-R5F MCU with real-time control and security, https://www.ti.com/product/AM2634-Q1

54 Renesas Electronics Corporation. RH850/U2A Zone/Domain Microcontroller Series, https://www.renesas.com/en/products/rh850-u2a

Comment 4:

The Shannon-entropy-based warning strategy is mainly used during the temperature rise phases of the battery system. However, the paper doesn't clearly explain how to determine that the battery system is in a temperature rise state. The authors should add a detailed description of how to identify that, which would make it easier for others to reproduce their method.

Response: Thank you very much for pointing out this important issue regarding the reproducibility of the Shannon-entropy-based warning strategy. We agree that the original manuscript does not provide sufficient details on how the temperature-rise segments are identified. Following your suggestion, we add a dedicated description entitled "Identification of the temperature rise segment" in the Methods section. Specifically, temperature data following a time gap longer than 30 min are treated as a new independent temperature segment, and the temperatures at the initial time of each segment are used as new references for calculating the relative temperature variations. The average temperature variation across all valid sensing points is then evaluated using linear least-squares fitting within a sliding window of 500 samples with a step size of one sample. For each independent temperature segment, the continuous interval starting from the reference time and during which the fitted slope remains non-negative ($k^{3}\ 0$) is defined as a temperature-rise segment, and the segment terminates when the fitted slope first becomes negative ($k < 0$). This revision explicitly defines the reference-reset criterion, fitting procedure, and start/termination conditions of the temperature-rise segment, thereby improving the clarity and reproducibility of the proposed Shannon-entropy-based warning strategy. All corresponding modifications are highlighted in yellow in the revised manuscript and are shown below.

In Method:

Identification of the temperature rise segment. Considering that prolonged rest allows the overall temperature field of the battery system under real-world vehicle operation to gradually approach a relatively stable state, when the time gap between two consecutive data records exceeds 30 minutes, the subsequent temperature data are treated as a new independent temperature segment in this work. For each segment, the temperatures measured at all sensing locations at the initial time point are taken as new references, such that the temperature variation at each location is reset to 0. Based on this reference, the average temperature variation across all valid temperature sensing points is then calculated, and its local temporal trend is characterized by using linear least squares fitting within a sliding window of 500 samples with a step size of one sample. For each independent temperature segment, the continuous interval starting from the reference time during which the fitted slope remained non-negative ($k \geq 0$) is defined as a temperature rise segment. The segment is considered to terminate when the fitted slope first became negative ($k < 0$).

Comment 5:

The paper proposes a "multi-model collaborative" framework that incorporates a temperature threshold, a temperature change rate threshold, a Shannon entropy change rate threshold. However, the description of the specific decision logic governing the "collaboration" among these models is vague. It is suggested that the final decision logic for the multi-model collaborative early warning be explicitly clarified, for instance, in the form of a flowchart or formalized conditional statements.

Response: Thank you for pointing out that the decision logic of the multi-model collaborative warning framework was not sufficiently explicit in the original manuscript. We agree that clearly defining how the individual warning criteria contribute to the final warning decision is important for understanding and reproducing the proposed framework. Following your suggestion, we explicitly clarify the final decision logic in the Warning framework overview section. Specifically, the four warning criteria, including the temperature threshold, temperature change rate threshold, Shannon entropy boundary, and Shannon entropy change rate threshold, operate independently and in parallel. A thermal runaway warning is issued immediately once any one of these criteria is triggered, without requiring the remaining criteria to be satisfied simultaneously. This additional description directly clarifies the collaborative operation mechanism of the proposed framework and improves its reproducibility. The corresponding statement has been added to the revised manuscript and highlighted in yellow, as shown below.

In Warning framework overview:

Building upon this, the four warning criteria described above operate independently and in parallel. A thermal runaway warning signal is issued immediately once any one of the criteria is triggered, without requiring the remaining criteria to be satisfied simultaneously.

Comment 6:

It is unclear whether the calibration of the parameters for this boundary model is a "one-time" procedure. In other words, for a battery system of a specific model/configuration, is calibration required only once in its fresh state? Alternatively, does this relationship change with battery aging, necessitating periodic re-calibration?

Response: Thank you for raising this important point. We agree that the calibration procedure and its potential dependence on battery aging should be clarified. In the present study, the parameters of the Shannon entropy boundary model are calibrated using representative normal-operation data for a given battery-system configuration, and the calibrated parameters are subsequently kept fixed during the warning process rather than being periodically recalibrated. However, we acknowledge that the stability of this calibrated relationship over the entire battery lifetime has not yet been systematically investigated. Battery aging, different material chemistries, and variations in system architecture may alter the thermal characteristics and spatial temperature distribution of the battery system, which could potentially affect the Shannon entropy relationship and the corresponding warning boundary. Therefore, based on the current experimental evidence, we cannot conclude that the same calibration remains universally valid throughout the entire battery lifetime or across different battery systems. To explicitly clarify this limitation, we have added the corresponding discussion to the Conclusion of the revised manuscript. We state that the effects of battery aging, different material chemistries, and system architecture on the stability of the developed Shannon

entropy model have not yet been systematically evaluated, and that its transferability across battery systems and operating conditions warrants further investigation. This clarification also highlights that revalidation or recalibration under substantial aging or different system configurations may be necessary in future studies. The corresponding statement has been added to the revised manuscript and highlighted in yellow, as shown below.

In Conclusion:

Despite the results of this study are promising, there are certain limitations that warrant further investigation. The number of failure samples and the diversity of failure modes covered in the validation remain limited, and the applicability of the proposed framework to internal short circuits, mechanical damage and other complex failure modes requires further validation. Furthermore, the effects of battery aging, different material chemistries, and system architectures on the stability of the developed Shannon entropy model have not yet been systematically evaluated, warranting further investigation into its transferability across battery systems and operating conditions. Hence, future work will further expand the calibration and validation datasets across mentioned factors to establish a model parameter library, enabling appropriate parameters to be updated through over-the-air (OTA) deployment and thereby improving the generalizability of the warning framework across different battery systems and operating conditions.

Comment 7:

Using a voltage variation rate of 1 V/s for the parallel-cell voltage as the reference criterion for fault warning in the conventional BMS does not fully represent the existing, more sophisticated warning strategies in the industry. To more fairly and convincingly demonstrate the advancement of the proposed framework, it is recommended to add comparisons with more comprehensive conventional warning strategies.

Response: Thank you very much for this important comment. We agree that using only a parallel-cell voltage variation rate of 1 V/s as the reference criterion does not fully represent the more comprehensive thermal runaway warning strategies currently available. Following your suggestion, we therefore add a broader benchmark comparison with three representative thermal runaway warning strategies reported in the literature, including a temperature-based composite threshold strategy, a temperature variance–local overheating index (LOI) dual-threshold strategy, and a modified standardized cumulative sum (CUSUM) method. To ensure a fair comparison, the same high-density distributed temperature data are used as the input for all benchmark strategies and the proposed framework. The comparison results are summarized in Table 2. The temperature-based composite threshold strategy identifies all thermal runaway events, but its warning times are consistently later than those of the proposed framework. In contrast, neither the temperature variance–LOI dual-threshold strategy nor the modified standardized CUSUM method successfully identifies all thermal runaway scenarios. These comparisons demonstrate that the proposed framework provides a more favorable overall performance in terms of both warning timeliness and detection capability across different failure scenarios. Accordingly, the 1 V/s parallel-cell voltage variation rate is retained only as a reference representing the warning response available from the conventional BMS, whereas the advancement of the proposed framework is now further evaluated against multiple existing warning strategies. Detailed descriptions and parameter settings of the three benchmark methods are provided in Supplementary Note 4. All corresponding revisions are highlighted in yellow in the revised manuscript and are shown below.

In “Thermal runaway warning performance”

Collectively, these results disentangle the respective contributions of high-density temperature sensing and the multi-model collaborative warning framework to thermal runaway warning performance. High-density temperature sensing enhances the observability of localized and spatially heterogeneous thermal anomalies, whereas the proposed warning framework further translates this sensing advantage into earlier and more reliable warning performance across different failure modes. The integration of high-density sensing with multi-model collaborative warning framework therefore provides a more reliable basis for thermal runaway early warning than conventional sparse temperature monitoring combined with a single warning strategy.

Table 2 Performance comparison of existing warning methods using distributed temperature sensing as input.

Warning strategy	Warning time			
	Overheating	Overcharging	Vehicle 1	Vehicle 4
Temperature-based composite threshold strategy [50]	13:53:45	11:15:21	17:03:00	19:11:52
Temperature variance and LOI dual-threshold warning [51]	14:05:31	None	None	None
Modified standardized CUSUM method [52]	13:53:43	11:18:24	None	None
**The proposed warning framework**	13:53:42	11:10:50	17:02:32	19:11:29

In Reference:

50 Yue, Y. et al. Early warning of thermal runaway in large-capacity LiFePO4 batteries based on distributed optical fiber. J. Energy Storage 141, 119280 (2026).

51 Wang, S. et al. Infrared imaging investigation of temperature fluctuation and spatial distribution for a large laminated lithium－ion power battery. Applied Thermal Engineering 152, 204-214 (2019).

52 Vu, L. A. et al. A real-time framework for early detection and severity prediction of thermal runaway in Li-ion batteries. J. Energy Storage 135, 118310 (2025).

In Supplementary Information:

Supplementary Note 4. Details of the existing warning methods for performance comparison

Method 1: Temperature-based composite threshold strategy

Let $T_{i,t}$ denote the temperature measured at the i-th sensing point at time t, and let Ω_t denote the set of temperature sensing points at that time. The maximum and minimum temperatures of the battery system are defined as

$$T_{max,t} = \max_{i\epsilon\Omega_t} T_{i,t} \tag{1}$$

$$T_{min,t} = \min_{i\epsilon\Omega_t} T_{i,t} \tag{2}$$

The instantaneous temperature range, used here to quantify temperature non-uniformity across the battery system, is calculated as

$$\Delta T_{range,t} = T_{max,t} - T_{min,t} \tag{3}$$

This metric captures the temperature contrast between the hottest and coolest sensing points. Unlike a spatial temperature gradient, it does not require information on the physical distances between sensing points and can therefore accommodate different sensor numbers and spatial arrangements at the module and pack levels. To characterize the temporal evolution of temperature, the temperature rise rate at the i-th sensing point is calculated from two consecutive measurements using the actual elapsed time:

$$\mathrm{R}_{i,t_k} = \frac{T_{i,t_k} - T_{i,t_{k-1}}}{t_k - t_{k-1}} \tag{4}$$

To suppress short-term measurement fluctuations, a moving average over W consecutive sampling points is applied:

$$\overline{\mathrm{R}}_{i,t_k} = \frac{1}{W} \sum_{j=k-W+1}^{k} \mathrm{R}_{i,t_j} \tag{5}$$

The maximum temperature rise rate of battery system is subsequently obtained from all valid sensing points:

$$\mathrm{R}_{max,t_k} = \max_{i\epsilon\Omega_{t_k}} \overline{\mathrm{R}}_{i,t_k} \tag{6}$$

Following the composite-threshold logic of the reference method and its adaptation to the present datasets, a thermal runaway warning is issued when the maximum temperature exceeds its prescribed threshold and either the maximum temperature rise rate or the temperature range exceeds the corresponding threshold:

$$T_{max,t} \geq T_{th} \ \wedge \ \left[\mathrm{R}_{max,t} \geq R_{th} \ \vee \ \Delta T_{range,t} \geq \Delta T_{th}\right] \ = 1 \tag{7}$$

where T_{th}, R_{th}, and ΔT_{th} denote the thresholds for the maximum temperature, maximum temperature rise rate, and temperature range, respectively. Based on the normal operating data collected at the lab-scale module level and at pack level

under real-world vehicle operation, the parameters of this method are calibrated to suppress false warnings under normal operation. The final parameter settings adopted for the battery module and battery pack are summarized below.

**Parameters**	**Module-level**	**Pack-level**
Moving average window $\boldsymbol{W}$	20	20
Maximum temperature threshold $\boldsymbol{T_{th}}$	100 ℃	100 ℃
Maximum temperature rise rate threshold $\boldsymbol{R_{th}}$	0.2 ℃/s	0.2 ℃/s
Temperature range threshold $\boldsymbol{\Delta T_{th}}$	20	20

Method 2: temperature variance and LOI dual-threshold warning

The temperature variance is employed to quantify the spatial non-uniformity of the distributed temperature field. At the t-th time step, the battery system contains M valid temperature sensing points, and the measured temperature at the j-th sensing point is denoted by $T_{t,j}$. The spatially averaged temperature is calculated as

$$\bar{T}_t = \frac{1}{M}\sum_{j=1}^{M} T_{t,j} \tag{8}$$

Accordingly, the temperature variance of the battery system is defined as

$$T_{var,t}^2 = \frac{1}{M}\sum_{j=1}^{M}\left(T_{t,j} - \bar{T}_t\right)^2 \tag{9}$$

where $T_{\mathrm{var},t}^2$ represents the dispersion of the distributed temperature measurements at the t-th time step. A larger temperature variance indicates a greater temperature difference among the sensing points and, consequently, a more non-uniform temperature field.

In addition to the numerical dispersion of the temperature field, the local overheating index (LOI) is introduced to characterize the spatial concentration of high-temperature sensing points. At each step, all valid temperature measurements are ranked in descending order, and the highest $\eta\%$ of the temperature sensing points are selected as the high-temperature subset. The sensing point with the maximum temperature is regarded as the hotspot center. Let $\left(x_{t,max}, y_{t,max}\right)$ denote the normalized spatial coordinates of the hotspot center and let $\left(x_{t,k}, y_{t,k}\right)$ denote the normalized coordinates of the k-th sensing point in the high-temperature subset. The LOi is calculated as

$$LOI_t = \frac{1}{N_h}\sum_{k=1}^{N_h}\sqrt{\left(x_{t,k} - x_{t,max}\right)^2 + \left(y_{t,k} - y_{t,max}\right)^2} \tag{10}$$

where N_h is the number of sensing points included in the high temperature subset. The spatial coordinates are normalized along each direction before calculation to eliminate the influence of the physical dimensions of the battery system. A smaller LOI value indicates that the high temperature sensing points are more strongly concentrated around the hotspot center, whereas a larger LOI value indicates a more dispersed high-temperature distribution.

Temperature variance and LOI characterize two complementary features of thermal anomalies. The temperature variance reflects the overall non-uniformity of the distributed temperature field, whereas the LOI describes the spatial concentration of the high temperature region. Therefore, a thermal anomaly is identified only when a significant increase in temperature non-uniformity is accompanied by pronounced local hotspot concentration. The instantaneous warning state at the t-th time step is defined as

$$A_t = \begin{cases} 1, & T_{var,t}^2 \geq T_{var,th}^2 \ and \ LOI_t \leq LOI_{th} \\ 0, & otherwise \end{cases} \tag{11}$$

where $T_{\mathrm{var,th}}^2$ is the upper warning threshold for temperature variance and $\mathrm{LOI_{th}}$ is the lower warning threshold for the local overheating index. Based on the normal operating data collected at the lab-scale module level and at pack level under real-world vehicle operation, the parameters of this method are calibrated to suppress false warnings under normal operation. The final parameter settings adopted for the battery module and battery pack are summarized below.

**Parameters**	**Module-level**	**Pack-level**
Upper threshold of temperature variance $\boldsymbol{T_{var,th}^2}$	50	50
Lower threshold of local overheating index $\boldsymbol{LOI_{th}}$	0.2	0.2

Proportion of high-temperature sensing points $\boldsymbol{\eta}$	10%	10%

Method 3: Modified standardized CUSUM method

For the temperature signal $T_{i,t}$ measured by the i-th sensing point at time t, an adaptive mean is first constructed using an exponentially weighted moving average:

$$\mu_{i,t} = (1-\beta)\mu_{i,t-1} + \beta T_{i,t} \tag{12}$$

where $\mu_{i,t}$ is the adaptive mean temperature of the i-th sensing point at time t, and β is the mean updating coefficient. Subsequently, the sample standard deviation of the temperature fluctuations is calculated using a sliding window with a length of W:

$$\sigma_{i,t} = \sqrt{\frac{1}{W-1}\sum_{j=t-W+1}^{t}\left(T_{i,j} - \bar{T}_{i,t}\right)^2} \tag{13}$$

where $\bar{T}_{i,t}$ denotes the average temperature of the i-th sensing point within the current sliding window. Under some operating conditions, the temperature fluctuation may be extremely small, and direct standardization may excessively amplify minor disturbances. Therefore, a lower bound $\sigma_{\min}$ is introduced for the standard deviation, and the standardized temperature residual is calculated as:

$$z_{i,t} = \frac{T_{i,t} - \mu_{i,t}}{\max\left(\sigma_{i,t}, \sigma_{min}\right)} \tag{14}$$

where $z_{i,t}$ represents the deviation of the current temperature from its recent normal operating state.
Based on the standardized residual, the positive CUSUM statistics are recursively updated as:

$$C_{i,t}^{+} = \max\left[0, C_{i,t-1}^{+} + z_{i,t} - k\right] \tag{15}$$

where $C_{i,t}^{+}$ is the positive CUSUM statistic of the i-th temperature sensing point at time t, and k is the reference drift parameter used to suppress the accumulation of minor positive temperature deviations during normal operation. When persistent abnormal heating occurs at a sensing point, its corresponding $C_{i,t}^{+}$ continuously increases. In contrast, when the temperature fluctuation remains within the normal range, the CUSUM statistics remain at a low level or returns to zero.

For a battery module or battery pack containing M valid temperature sensing points, the system-level temperature warning statistic is defined as:

$$C_{sys,t} = \max_{i=1,2,\cdots,M} C_{i,t}^{+} \tag{16}$$

A thermal runaway warning is triggered when the positive CUSUM statistic of any valid temperature sensing point reaches the predefined threshold H:

$$C_{sys,t} \geq H \tag{17}$$

Based on the normal operating data collected at the lab-scale module level and at pack level under real-world vehicle operation, the parameters of the modified standardized CUSUM method are calibrated to suppress false warnings under normal operation. The final parameter settings adopted for the battery module and battery pack are summarized below.

**Parameters**	**Module-level**	**Pack-level**
Mean updating coefficient $\boldsymbol{\beta}$	0.30	0.30
Length of sliding window $\boldsymbol{W}$	20	20
Minimum standard deviation $\boldsymbol{\sigma_{min}}$	0.05 °C	0.05 °C
Reference drift parameter $\boldsymbol{k}$	0.50	0.50
Warning threshold $\boldsymbol{H}$	7.00	30.00

Comment 8:

The statement that "the per-vehicle application cost of the proposed warning scheme is only approximately 40 USD" would benefit from a brief cost breakdown analysis in the Discussion or Supplementary Materials. Clarifying how this figure is derived and whether it includes the cost of the interrogator (in addition to the fiber optic sensors) would significantly strengthen the persuasiveness of the commercialization potential of the proposed scheme.

Response: Thank you very much for this constructive suggestion. We agree that the original manuscript does not sufficiently

explain the derivation and scope of the estimated per-vehicle cost. Following your suggestion, we add a detailed cost analysis in Supplementary Note 1 and provide a corresponding cross-reference in the caption of Fig. 1. The analysis is based on the actual sensing configuration used in this study, including 540 temperature sensing gratings, one onboard miniaturized interrogator, cables and accessories, and installation materials. At the supplier-quotation-based production scale of 10^4 vehicles, the corresponding per-vehicle costs are 64.80, 35.50, 2.15, and 0.72 USD, respectively, resulting in a total hardware cost of 103.17 USD per vehicle. Therefore, the cost estimate explicitly includes the onboard interrogator. To further clarify the cost evolution with increasing deployment scale, we introduce a component-level technology learning-curve model supported by previous studies on battery and energy-technology cost forecasting. Because historical cost data across multiple production scales are unavailable for the proposed scheme, the adopted learning rates are treated as scenario-based assumptions rather than empirically fitted parameters. Specifically, a learning rate of 10% is assigned to the temperature-sensing gratings and interrogator, whereas a learning rate of 5% is assigned to the cables, accessories, and installation materials. The projected total hardware costs at deployment scales of 10^4, 10^5, 10^6, and 10^7 vehicles are 103.17, 73.10, 51.85, and 36.82 USD per vehicle, respectively. Accordingly, the "approximately 40 USD per vehicle" stated in the manuscript represents the projected hardware cost under a large-scale deployment scenario of approximately 10^7 vehicles rather than the current quotation-based cost. All corresponding modifications are highlighted in yellow in the revised manuscript and are shown below. We again appreciate your suggestion, which substantially improves the transparency and persuasiveness of the commercialization analysis.

In the caption of Fig. 1:

(d) The cost of the proposed warning scheme versus the EV application market, and the detailed cost analysis is provided in **Supplementary Note 1**.

In Supplementary Information:

Supplementary Note 1. Cost analysis of the proposed warning scheme

The per-vehicle hardware cost of the proposed warning scheme is estimated according to the actual sensing configuration deployed in one battery pack. The system comprises 540 temperature sensing gratings, one onboard miniaturized interrogator, cables and accessories, and the materials required for sensor installation and fixation. The reference costs are derived from a supplier (Shanghai SensChain Co., Ltd.) quotation corresponding to a production scale of 104 vehicles. At this scale, the unit price of a temperature sensing grating is 0.12 USD per sensing point. Therefore, the cost of the 540 temperature sensing points installed in one battery pack is calculated as:

$$C_{FBG,10^4} = 540 \times 0.12 = 64.80 \ \ (USD/vehicle)$$

The per-vehicle costs of the onboard miniaturized interrogator, cables and accessories, and installation materials are 35.50, 2.15, and 0.72 USD, respectively. Accordingly, the total hardware cost at the reference production scale is:

$$C_{total,10^4} = 64.80 + 35.50 + 2.15 + 0.72 = 103.17 \ \ (USD/vehicle)$$

Furthermore, this study adopts a component-level technology learning-curve model to estimate the cost evolution of the proposed warning scheme with increasing deployment scale. This model is appropriate because the system mainly consists of standardized hardware components whose unit costs can decrease through cumulative manufacturing experience, process automation, yield improvement, and economies of scale [1]. Technology learning curves, which describe unit cost as a power-law function of cumulative production or deployment, have been widely used for energy and battery technology cost forecasting [2]. For the i-th hardware component, the unit cost at a cumulative deployment scale Q is expressed as:

$$C_{i,Q} = C_{i,Q_0}(Q/Q_0)^{\ln(1-LR_i)/ln2}$$

where $C_{i,Q}$ and C_{i,Q_0} are the per-vehicle cost of component i at deployment scale Q and Q_0, respectively. LR_i is the learning rate of component i.

Since the historical cost data across multiple production scales are not currently available for the proposed warning scheme, the learning rates adopted in this analysis are treated as scenario-based assumptions rather than parameters empirically identified from historical cost data. In its component-level technology cost projections, the International Energy Agency applies a learning rate of 10% to automotive battery cells and fuel cell stacks and a learning rate of 5% to battery packaging and fuel cell balance-of-plant components [3]. Based on these analogous component categories, a learning rate of 10% is assigned to the temperature sensing gratings and onboard miniaturized interrogator, while a learning rate of 5% is

assigned to cables and accessories, and installation materials in this study. Building upon this, the projected per-vehicle costs at deployment scales of 10^4, 10^5, 10^6, and 10^7 vehicles are summarized below.

**Deployment scales**	**Temperature sensing gratings**	**Interrogator**	**Cables and accessories**	**Installation materials**	**Total cost**
10^4 vehicles	64.80 USD	35.50 USD	2.15 USD	0.72 USD	103.17 USD
10^5 vehicles	45.66 USD	25.02 USD	1.81 USD	0.61 USD	73.10 USD
10^6 vehicles	32.18 USD	17.63 USD	1.53 USD	0.51 USD	51.85 USD
10^7 vehicles	22.68 USD	12.42 USD	1.29 USD	0.43 USD	36.82 USD

1 Ziegler, M. S. & Trancik, J. E. Re-examining rates of lithium-ion battery technology improvement and cost decline. Energy & Environmental Science 14, 1635-1651 (2021).

2 Wu, W., Zhai, H. & Holubnyak, E. Technological evolution of large-scale blue hydrogen production toward the U.S. Hydrogen Energy Earthshot. Nat. Commun. 15, 5684 (2024).

3 Unit cost reductions for selected technologies in the Sustainable Development Scenario, 2020 – 2070, https://www.iea.org/data-and-statistics/charts/unit-cost-reductions-for-selected-technologies-in-the-sustainable-development-scenario-2020-2070

Responses to Reviewer #2:

This paper proposes a multi-model collaborative thermal runaway early warning framework based on distributed temperature sensing, aiming to address two well-defined limitations inherent to conventional BMS: sparse temperature information and monolithic diagnostic frameworks. The authors exploit fiber optic sensors to achieve high-density temperature sensing throughout the battery system with minimal wiring complexity, and the long-term field validation under real-world vehicle scenarios demonstrates the potential engineering applicability of the proposed scheme. Furthermore, the established functional relationship between temperature Shannon entropy and mean temperature variation is novel and insightful. Overall, the manuscript exhibits a well-organized structure, maintains a clear application-oriented focus, and holds potential value for next-generation battery safety management. However, several concerns remain to be clarified before it can be accepted for publication.

Response: We greatly appreciate your recognition of the distributed fiber optic temperature sensing scheme, the multi-model collaborative thermal runaway warning framework, the Shannon-entropy-based warning strategy, and the potential engineering applicability of this work. Following your comments, we have carefully revised the manuscript and provided additional analyses and clarifications. Specifically, we have further clarified the cost composition of the proposed warning scheme and the methodology used to project its cost evolution with increasing deployment scale; supplemented the temperature responses before and after mechanical crush under different operating conditions; provided the fitting results and goodness-of-fit metrics for the Shannon entropy model; clarified the identification procedure for temperature-rise segments; and added systematic comparisons between the proposed warning framework and existing thermal runaway warning methods reported in the literature. These revisions further strengthen the methodological transparency, reproducibility, and persuasiveness of the manuscript. We sincerely appreciate the reviewer's constructive suggestions, which have helped us substantially improve the quality and completeness of the manuscript. All corresponding revisions have been highlighted in yellow in the revised manuscript, and detailed responses to each comment and the corresponding modifications are provided below.

Comment 1:

In the final paragraph of the Introduction, the authors mention the cost of the proposed warning scheme. However, the cost structure of this scheme remains unclear. The authors are encouraged to provide a more detailed explanation of the main cost components so that they can better support the claimed cost-effectiveness of the proposed approach.

Response: Thank you for this valuable suggestion. We agree that the original manuscript only reports the approximate per-vehicle cost of the proposed warning scheme without sufficiently explaining its cost structure, which limits the transparency of the cost-effectiveness analysis. Following your suggestion, we add a detailed cost analysis in Supplementary Note 1 and provide a corresponding cross-reference in the caption of Fig. 1. The analysis is based on the actual sensing configuration deployed in one battery pack, including 540 temperature-sensing gratings, one onboard miniaturized interrogator, cables and accessories, and installation materials. At the supplier-quotation-based production scale of 10^4 vehicles, the corresponding per-vehicle costs are 64.80, 35.50, 2.15, and 0.72 USD, respectively, resulting in a total hardware cost of 103.17 USD per vehicle. Therefore, the cost assessment explicitly includes the onboard miniaturized interrogator. To further characterize the cost variation with increasing deployment scale, we adopt a component-level technology learning-curve model supported by previous studies on battery and energy-technology cost forecasting. Because historical cost data across multiple production scales are not available for the proposed scheme, the learning rates are treated as scenario-based assumptions rather than empirically fitted parameters. Based on this analysis, the projected per-vehicle hardware costs at deployment scales of 10^4, 10^5, 10^6, and 10^7 vehicles are 103.17, 73.10, 51.85, and 36.82 USD, respectively. Thus, the approximately 40 USD per-vehicle cost stated in the Introduction corresponds to the projected hardware cost under a large-scale deployment scenario of approximately 10^7 vehicles. All corresponding modifications are highlighted in yellow in the revised manuscript and are given below. We again appreciate your suggestion, which improves the transparency of the cost structure and provides stronger support for the claimed cost-effectiveness and commercialization potential of the proposed scheme.

In the caption of Fig. 1:

(d) The cost of the proposed warning scheme versus the EV application market, and the detailed cost analysis is provided in **Supplementary Note 1**.

In Supplementary Information:

Supplementary Note 1. Cost analysis of the proposed warning scheme

The per-vehicle hardware cost of the proposed warning scheme is estimated according to the actual sensing configuration deployed in one battery pack. The system comprises 540 temperature sensing gratings, one onboard miniaturized interrogator, cables and accessories, and the materials required for sensor installation and fixation. The reference costs are derived from a supplier (Shanghai SensChain Co., Ltd.) quotation corresponding to a production scale of 104 vehicles. At this scale, the unit price of a temperature sensing grating is 0.12 USD per sensing point. Therefore, the cost of the 540 temperature sensing points installed in one battery pack is calculated as:

$$C_{FBG,10^4} = 540 \times 0.12 = 64.80 \ \ (USD/vehicle)$$

The per-vehicle costs of the onboard miniaturized interrogator, cables and accessories, and installation materials are 35.50, 2.15, and 0.72 USD, respectively. Accordingly, the total hardware cost at the reference production scale is:

$$C_{total,10^4} = 64.80 + 35.50 + 2.15 + 0.72 = 103.17 \ \ (USD/vehicle)$$

Furthermore, this study adopts a component-level technology learning-curve model to estimate the cost evolution of the proposed warning scheme with increasing deployment scale. This model is appropriate because the system mainly consists of standardized hardware components whose unit costs can decrease through cumulative manufacturing experience, process automation, yield improvement, and economies of scale [1]. Technology learning curves, which describe unit cost as a power-law function of cumulative production or deployment, have been widely used for energy and battery technology cost forecasting [2]. For the i-th hardware component, the unit cost at a cumulative deployment scale Q is expressed as:

$$C_{i,Q} = C_{i,Q_0}(Q/Q_0)^{\ln(1-LR_i)/ln2}$$

where $C_{i,Q}$ and C_{i,Q_0} are the per-vehicle cost of component i at deployment scale Q and Q_0, respectively. LR_i is the learning rate of component i.

Since the historical cost data across multiple production scales are not currently available for the proposed warning scheme, the learning rates adopted in this analysis are treated as scenario-based assumptions rather than parameters empirically identified from historical cost data. In its component-level technology cost projections, the International Energy Agency applies a learning rate of 10% to automotive battery cells and fuel cell stacks and a learning rate of 5% to battery packaging and fuel cell balance-of-plant components [3]. Based on these analogous component categories, a learning rate of 10% is assigned to the temperature sensing gratings and onboard miniaturized interrogator, while a learning rate of 5% is assigned to cables and accessories, and installation materials in this study. Building upon this, the projected per-vehicle costs at deployment scales of 10^4, 10^5, 10^6, and 10^7 vehicles are summarized below.

**Deployment scales**	**Temperature sensing gratings**	**Interrogator**	**Cables and accessories**	**Installation materials**	**Total cost**
10^4 vehicles	64.80 USD	35.50 USD	2.15 USD	0.72 USD	103.17 USD
10^5 vehicles	45.66 USD	25.02 USD	1.81 USD	0.61 USD	73.10 USD
10^6 vehicles	32.18 USD	17.63 USD	1.53 USD	0.51 USD	51.85 USD
10^7 vehicles	22.68 USD	12.42 USD	1.29 USD	0.43 USD	36.82 USD

1 Ziegler, M. S. & Trancik, J. E. Re-examining rates of lithium-ion battery technology improvement and cost decline. Energy & Environmental Science 14, 1635-1651 (2021).

2 Wu, W., Zhai, H. & Holubnyak, E. Technological evolution of large-scale blue hydrogen production toward the U.S. Hydrogen Energy Earthshot. Nat. Commun. 15, 5684 (2024).

3 Unit cost reductions for selected technologies in the Sustainable Development Scenario, 2020 – 2070, https://www.iea.org/data-and-statistics/charts/unit-cost-reductions-for-selected-technologies-in-the-sustainable-development-scenario-2020-2070

Comment 2:

The authors fit the functional relationship between the temperature Shannon entropy and the mean temperature variation in the battery system using a customized equation. However, no goodness-of-fit metrics are reported in either the main text or the supplementary materials.

Response: Thank you for pointing out this omission. We agree that quantitative goodness-of-fit metrics are necessary to evaluate the reliability of the nonlinear relationship established between temperature Shannon entropy and average temperature variation. Following your suggestion, we further evaluate the fitting performance using the coefficient of determination (R^2) and add the corresponding fitting results in Supplementary Fig. 7. As shown in Supplementary Fig. 7, the R^2 values of the nonlinear fitting are 0.9848 at the module level and 0.9043 at the pack level, indicating that the adopted nonlinear function provides a good representation of the relationship between temperature Shannon entropy and average temperature variation at both scales. The fitting curves and R^2 values are now explicitly presented in Supplementary Fig. 7, while the corresponding parameters of the Shannon entropy warning boundary are summarized in Supplementary Table 4. All corresponding modifications are highlighted in yellow in the revised manuscript and are given below.

In Method:

The fitting results are shown in **Supplementary Fig. 7.** The parameters of Shannon entropy waring boundary are summarized in **Supplementary Table 4**.

In Supplementary Information:

Supplementary Fig. 7. Fitting results for the nonlinear relationship between temperature Shannon entropy and the average temperature variation. The average temperature variation is binned at 0.5 °C intervals, and the maximum Shannon entropy within each interval, together with its corresponding average temperature variation, is selected for fitting data. (a) Module-level; (b) Pack-level.

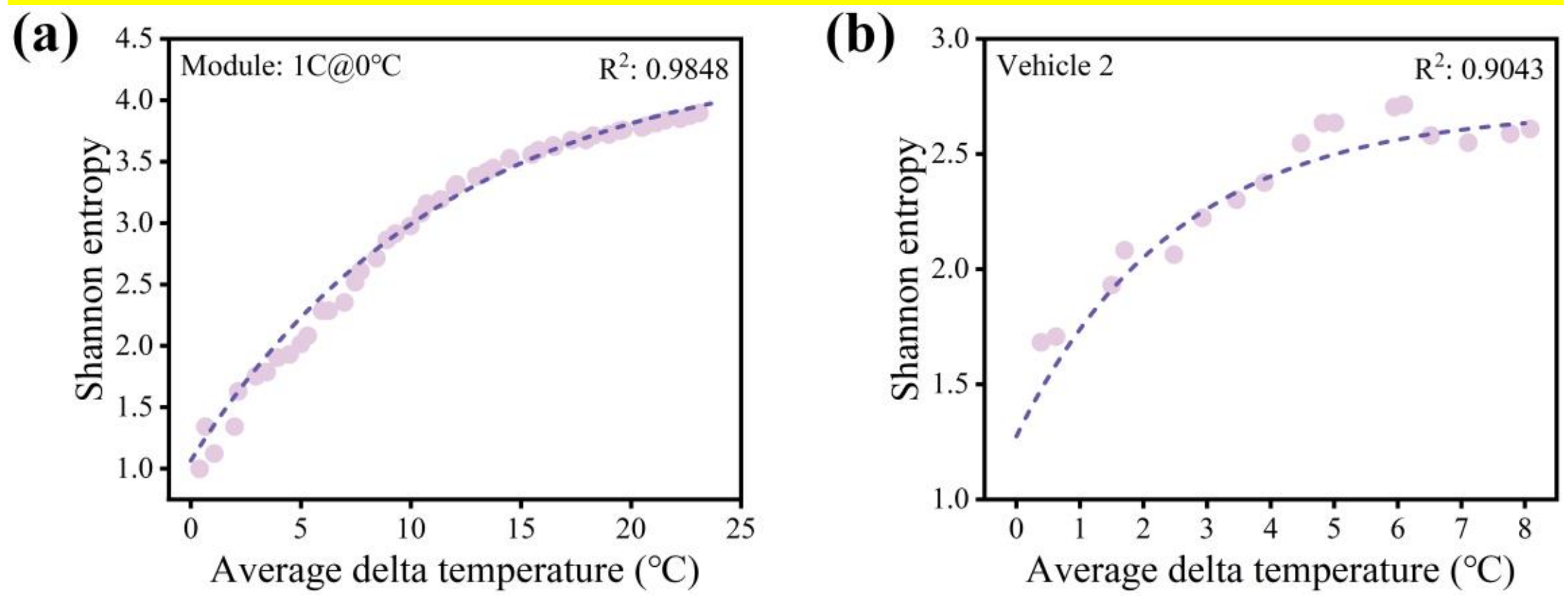

Comment 3:

The authors only present the surface temperature differences during 0.5 C constant-current charging before and after mechanical crush in Fig. 3(c). Whether such differences persist under other lower current rates or during discharging?

Response: Thank you for raising this important point. Following your suggestion, we further examine the surface temperature evolution of the mechanically crushed cell under a lower current rate and during discharging. The corresponding results are added as Supplementary Fig. 1, including 0.5 C constant-current discharge, 0.3 C constant-current charge, and 0.3 C constant-current discharge. The results show that the difference in surface temperature evolution before and after mechanical crushing persists under both the lower current rate and the discharging conditions. During 0.5 C discharge and 0.3 C charge, the mechanically crushed cell exhibits a greater surface temperature increase than before crushing. During 0.3 C discharge, the temperature evolution after crushing also differs markedly from the pre-crush response, although its variation pattern is distinct from that observed during charging. These results demonstrate that the abnormal thermal response induced by mechanical crushing is not limited to the 0.5 C charging condition but remains observable under different current rates and current directions. A corresponding statement and cross-reference are added to the Temperature-Dependent Characteristics of

the Battery section, and the additional results are provided in Supplementary Fig. 1. All corresponding modifications are highlighted in yellow in the revised manuscript and are shown below.

In Temperature-Dependent Characteristics of the Battery:

Moreover, the corresponding surface temperature evolution under other C-rates and current directions is provided in **Supplementary Fig. 1**.

In Supplementary Information:

Evolution of battery surface temperature under various C-rates and current directions. (a) CC discharge at a current of 0.5 C; (b) CC charge at a current of 0.3 C; (c) CC discharge at a current of 0.3 C.

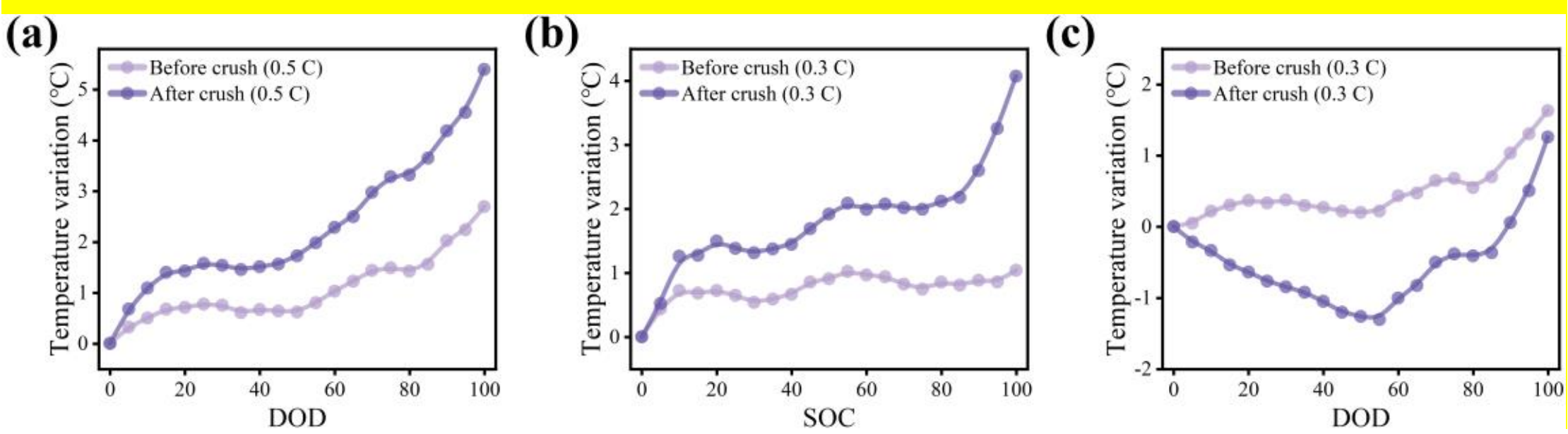

Comment 4:

The authors are advised to unify the terminology and address certain language-related details throughout the manuscript. For instance, both "fiber optic sensors" and "fiber-optic sensors" appear in the text. Although these issues do not significantly affect the content, consistent use of terminology would enhance the overall readability and professionalism of the paper.

Response: Thank you very much for carefully pointing out the inconsistencies in terminology and language usage throughout the manuscript. Following your suggestion, we conducted a comprehensive language and terminology check covering the main text, Highlights, figure captions, and Supplementary Information. Specifically, because “fiber optic sensor(s)” is the predominant form used in the title and main text, all occurrences of “fiber-optic sensor(s)” have been standardized as “fiber optic sensor(s).” Lengthy constructions containing multiple hyphens, such as “fiber-optic-sensor-based integrated sensing scheme,” have also been rephrased as “integrated sensing scheme based on fiber optic sensors” to improve clarity and readability. In addition, we have also corrected several grammatical, hyphenation, capitalization, and unit-formatting issues. All these modifications have been highlighted in yellow in the revised manuscript and are shown below. We again appreciate your careful examination, which has effectively improved the linguistic consistency, readability, and professionalism of the manuscript.

Representative changes:

“fiber-optic sensor(s)” → “fiber optic sensor(s)”

“mean temperature variation” → “average temperature variation”

“early-warning framework” → “earl warning framework”

Comment 5:

The warning lead times reported in the manuscript are only evaluated relative to the BMS warning strategy. The authors are encouraged to briefly discuss how these lead times compare with those reported for other advanced early-warning methods in the existing literature.

Response: Thank you very much for this valuable suggestion. We agree that evaluating the warning lead time only relative to the conventional BMS reference provides a limited basis for assessing the advancement of the proposed framework. Following your suggestion, we further compare the proposed framework with three representative thermal runaway warning strategies reported in the literature, including a temperature-based composite threshold strategy, a temperature variance–local

overheating index (LOI) dual-threshold strategy, and a modified standardized cumulative sum (CUSUM) method. Rather than directly comparing lead times reported in different studies, which may be affected by differences in battery type, failure mode, experimental conditions, sensing configuration, and the definition of the warning reference point, we reproduce these benchmark strategies using the same high-density distributed temperature data employed in this study. The comparison results are summarized in Table 2. The temperature-based composite threshold strategy successfully identifies all thermal runaway events, but its warning times are consistently later than those of the proposed framework, whereas the other two benchmark strategies fail to identify all failure scenarios. These results provide a more consistent basis for evaluating the warning lead time and overall detection performance of the proposed framework relative to existing advanced methods. Accordingly, the voltage variation rate of 1 V/s is retained as a conventional BMS reference, while the newly added benchmark comparison provides a broader evaluation against advanced thermal runaway warning strategies reported in the literature. Detailed descriptions and parameter settings of the three benchmark methods are provided in Supplementary Note 4. All corresponding modifications are highlighted in yellow in the revised manuscript and are shown below.

In "Thermal runaway warning performance"

Collectively, these results disentangle the respective contributions of high-density temperature sensing and the multi-model collaborative warning framework to thermal runaway warning performance. High-density temperature sensing enhances the observability of localized and spatially heterogeneous thermal anomalies, whereas the proposed warning framework further translates this sensing advantage into earlier and more reliable warning performance across different failure modes. The integration of high-density sensing with multi-model collaborative warning framework therefore provides a more reliable basis for thermal runaway early warning than conventional sparse temperature monitoring combined with a single warning strategy.

Table 2 Performance comparison of existing warning methods using distributed temperature sensing as input.

**Warning strategy**	**Warning time**			
	Overheating	Overcharging	Vehicle 1	Vehicle 4
Temperature-based composite threshold strategy [50]	13:53:45	11:15:21	17:03:00	19:11:52
Temperature variance and LOI dual-threshold warning [51]	14:05:31	None	None	None
Modified standardized CUSUM method [52]	13:53:43	11:18:24	None	None
**The proposed warning framework**	13:53:42	11:10:50	17:02:32	19:11:29

In Reference:

50 Yue, Y. et al. Early warning of thermal runaway in large-capacity LiFePO4 batteries based on distributed optical fiber. J. Energy Storage 141, 119280 (2026).
51 Wang, S. et al. Infrared imaging investigation of temperature fluctuation and spatial distribution for a large laminated lithium‐ion power battery. Applied Thermal Engineering 152, 204-214 (2019).
52 Vu, L. A. et al. A real-time framework for early detection and severity prediction of thermal runaway in Li-ion batteries. J. Energy Storage 135, 118310 (2025).

In Supplementary Information:

Supplementary Note 4. Details of the existing warning methods for performance comparison

Method 1: Temperature-based composite threshold strategy

Let $T_{i,t}$ denote the temperature measured at the i-th sensing point at time t, and let Ω_t denote the set of temperature sensing points at that time. The maximum and minimum temperatures of the battery system are defined as

$$T_{max,t} = \max_{i \epsilon \Omega_t} T_{i,t} \tag{1}$$

$$T_{min,t} = \min_{i \epsilon \Omega_t} T_{i,t} \tag{2}$$

The instantaneous temperature range, used here to quantify temperature non-uniformity across the battery system, is calculated as

$$\Delta T_{range,t} = T_{max,t} - T_{min,t} \quad (3)$$

This metric captures the temperature contrast between the hottest and coolest sensing points. Unlike a spatial temperature gradient, it does not require information on the physical distances between sensing points and can therefore accommodate different sensor numbers and spatial arrangements at the module and pack levels. To characterize the temporal evolution of temperature, the temperature rise rate at the i-th sensing point is calculated from two consecutive measurements using the actual elapsed time:

$$\mathrm{R}_{i,t_k} = \frac{T_{i,t_k} - T_{i,t_{k-1}}}{t_k - t_{k-1}} \quad (4)$$

To suppress short-term measurement fluctuations, a moving average over W consecutive sampling points is applied:

$$\overline{\mathrm{R}}_{i,t_k} = \frac{1}{W} \sum_{j=k-W+1}^{k} \mathrm{R}_{i,t_j} \quad (5)$$

The maximum temperature rise rate of battery system is subsequently obtained from all valid sensing points:

$$\mathrm{R}_{max,t_k} = \max_{i\epsilon\Omega_{t_k}} \overline{\mathrm{R}}_{i,t_k} \quad (6)$$

Following the composite-threshold logic of the reference method and its adaptation to the present datasets, a thermal runaway warning is issued when the maximum temperature exceeds its prescribed threshold and either the maximum temperature rise rate or the temperature range exceeds the corresponding threshold:

$$T_{max,t} \geq T_{th} \ \wedge \ [\mathrm{R}_{max,t} \geq R_{th} \ \vee \ \Delta T_{range,t} \geq \Delta T_{th}] \ = 1 \quad (7)$$

where T_{th}, R_{th}, and ΔT_{th} denote the thresholds for the maximum temperature, maximum temperature rise rate, and temperature range, respectively. Based on the normal operating data collected at the lab-scale module level and at pack level under real-world vehicle operation, the parameters of this method are calibrated to suppress false warnings under normal operation. The final parameter settings adopted for the battery module and battery pack are summarized below.

**Parameters**	**Module-level**	**Pack-level**
Moving average window $\boldsymbol{W}$	20	20
Maximum temperature threshold $\boldsymbol{T_{th}}$	100 ℃	100 ℃
Maximum temperature rise rate threshold $\boldsymbol{R_{th}}$	0.2 ℃/s	0.2 ℃/s
Temperature range threshold $\boldsymbol{\Delta T_{th}}$	20	20

Method 2: temperature variance and LOI dual-threshold warning

The temperature variance is employed to quantify the spatial non-uniformity of the distributed temperature field. At the t-th time step, the battery system contains M valid temperature sensing points, and the measured temperature at the j-th sensing point is denoted by $T_{t,j}$. The spatially averaged temperature is calculated as

$$\bar{T}_t = \frac{1}{M} \sum_{j=1}^{M} T_{t,j} \quad (8)$$

Accordingly, the temperature variance of the battery system is defined as

$$T_{var,t}^2 = \frac{1}{M} \sum_{j=1}^{M} \left(T_{t,j} - \bar{T}_t\right)^2 \quad (9)$$

where $T_{\mathrm{var},t}^2$ represents the dispersion of the distributed temperature measurements at the t-th time step. A larger temperature variance indicates a greater temperature difference among the sensing points and, consequently, a more non-uniform temperature field.

In addition to the numerical dispersion of the temperature field, the local overheating index (LOI) is introduced to characterize the spatial concentration of high-temperature sensing points. At each step, all valid temperature measurements are ranked in descending order, and the highest $\eta\%$ of the temperature sensing points are selected as the high-temperature subset. The sensing point with the maximum temperature is regarded as the hotspot center. Let $(x_{t,max}, y_{t,max})$ denote the

normalized spatial coordinates of the hotspot center and let $(x_{t,k}, y_{t,k})$ denote the normalized coordinates of the k-th sensing point in the high-temperature subset. The LOi is calculated as

$$LOI_t = \frac{1}{N_h}\sum_{k=1}^{N_h}\sqrt{\left(x_{t,k} - x_{t,max}\right)^2 + \left(y_{t,k} - y_{t,max}\right)^2} \tag{10}$$

where N_h is the number of sensing points included in the high temperature subset. The spatial coordinates are normalized along each direction before calculation to eliminate the influence of the physical dimensions of the battery system. A smaller LOI value indicates that the high temperature sensing points are more strongly concentrated around the hotspot center, whereas a larger LOI value indicates a more dispersed high-temperature distribution.

Temperature variance and LOI characterize two complementary features of thermal anomalies. The temperature variance reflects the overall non-uniformity of the distributed temperature field, whereas the LOI describes the spatial concentration of the high temperature region. Therefore, a thermal anomaly is identified only when a significant increase in temperature non-uniformity is accompanied by pronounced local hotspot concentration. The instantaneous warning state at the t-th time step is defined as

$$A_t = \begin{cases} 1, & T_{var,t}^2 \geq T_{var,th}^2 \ \ and \ \ LOI_t \leq LOI_{th} \\ 0, & otherwise \end{cases} \tag{11}$$

where $T_{\mathrm{var,th}}^2$ is the upper warning threshold for temperature variance and $\mathrm{LOI_{th}}$ is the lower warning threshold for the local overheating index. Based on the normal operating data collected at the lab-scale module level and at pack level under real-world vehicle operation, the parameters of this method are calibrated to suppress false warnings under normal operation. The final parameter settings adopted for the battery module and battery pack are summarized below.

**Parameters**	**Module-level**	**Pack-level**
Upper threshold of temperature variance $\boldsymbol{T_{var,th}^2}$	50	50
Lower threshold of local overheating index $\boldsymbol{LOI_{th}}$	0.2	0.2
Proportion of high-temperature sensing points $\boldsymbol{\eta}$	10%	10%

Method 3: Modified standardized CUSUM method

For the temperature signal $T_{i,t}$ measured by the i-th sensing point at time t, an adaptive mean is first constructed using an exponentially weighted moving average:

$$\mu_{i,t} = (1-\beta)\mu_{i,t-1} + \beta T_{i,t} \tag{12}$$

where $\mu_{i,t}$ is the adaptive mean temperature of the i-th sensing point at time t, and β is the mean updating coefficient. Subsequently, the sample standard deviation of the temperature fluctuations is calculated using a sliding window with a length of W:

$$\sigma_{i,t} = \sqrt{\frac{1}{W-1}\sum_{j=t-W+1}^{t}\left(T_{i,j} - \bar{T}_{i,t}\right)^2} \tag{13}$$

where $\bar{T}_{i,t}$ denotes the average temperature of the i-th sensing point within the current sliding window. Under some operating conditions, the temperature fluctuation may be extremely small, and direct standardization may excessively amplify minor disturbances. Therefore, a lower bound $\sigma_{\min}$ is introduced for the standard deviation, and the standardized temperature residual is calculated as:

$$z_{i,t} = \frac{T_{i,t} - \mu_{i,t}}{\max\left(\sigma_{i,t}, \sigma_{min}\right)} \tag{14}$$

where $z_{i,t}$ represents the deviation of the current temperature from its recent normal operating state.
Based on the standardized residual, the positive CUSUM statistics are recursively updated as:

$$C_{i,t}^{+} = \max\left[0, C_{i,t-1}^{+} + z_{i,t} - k\right] \tag{15}$$

where $C_{i,t}^{+}$ is the positive CUSUM statistic of the i-th temperature sensing point at time t, and k is the reference drift parameter used to suppress the accumulation of minor positive temperature deviations during normal operation. When persistent abnormal heating occurs at a sensing point, its corresponding $C_{i,t}^{+}$ continuously increases. In contrast, when the temperature fluctuation remains within the normal range, the CUSUM statistics remain at a low level or returns to zero.

For a battery module or battery pack containing M valid temperature sensing points, the system-level temperature warning statistic is defined as:

$$C_{sys,t} = \max_{i=1,2,\cdots,M} C_{i,t}^{+} \quad (16)$$

A thermal runaway warning is triggered when the positive CUSUM statistic of any valid temperature sensing point reaches the predefined threshold H:

$$C_{sys,t} \geq H \quad (17)$$

Based on the normal operating data collected at the lab-scale module level and at pack level under real-world vehicle operation, the parameters of the modified standardized CUSUM method are calibrated to suppress false warnings under normal operation. The final parameter settings adopted for the battery module and battery pack are summarized below.

**Parameters**	**Module-level**	**Pack-level**
Mean updating coefficient $\boldsymbol{\beta}$	0.30	0.30
Length of sliding window $\boldsymbol{W}$	20	20
Minimum standard deviation $\boldsymbol{\sigma_{min}}$	0.05 ℃	0.05 ℃
Reference drift parameter $\boldsymbol{k}$	0.50	0.50
Warning threshold $\boldsymbol{H}$	7.00	30.00

Comment 6:

The advancement of the fiber sensor against conventional thermal couple need to be further elaborated. Does the proposed method with Shannon Entropy also suitable for conventional temperature or electric signals? Please also discuss the underlying mechanistic meaning to enhance the physical significance of the feature, which will improve the scientific guidance in this field.

Response: Thank you for this valuable comment. Following your suggestion, we further clarify the advantages of the fiber optic sensing scheme over conventional temperature sensing and evaluate the applicability of the developed Shannon-entropy-based method to conventional temperature measurements. First, compared with conventional thermocouples, fiber optic sensors enable high-density temperature measurements with substantially reduced wiring complexity through multi-point multiplexing along a single fiber. In addition, we further conduct a temperature-step experiment to compare the thermal responses of the fiber optic sensor and thermocouple. The results show that the fiber optic sensor responds more rapidly to abrupt temperature changes than the thermocouple under the present test configuration, further demonstrating its capability to capture transient thermal anomalies (Supplementary Fig. 3). Second, to examine whether the developed Shannon-entropy-based method is applicable to conventional temperature measurements, we apply the same warning framework using either the sparse temperature measurements available from a conventional BMS or the high-density temperature measurements employed in this study. The results are summarized in Table 1. The Shannon entropy method remains applicable when conventional sparse temperature measurements are used as input, demonstrating that the method itself is not restricted to distributed temperature data acquired by the fiber optic sensors. However, the comparison also shows that increasing the spatial density of temperature measurements enables earlier identification of abnormal thermal behavior and reduces the risk of missed warnings in several failure scenarios. This is particularly important for localized thermal anomalies, which may not be adequately captured by sparsely distributed temperature sensors. These additional results further demonstrate that the advantage of the proposed sensing scheme arises not only from the applicability of the warning method itself, but also from the enhanced spatial and temporal observability of abnormal thermal behavior provided by high-density fiber optic temperature sensing. All corresponding modifications are highlighted in yellow in the revised manuscript and are shown below.

In "Thermal runaway warning performance"

To further evaluate the impact of the spatial density of temperature measurements on thermal runaway warning performance, we compare sparse temperature measurements available from a conventional BMS with the high-density temperature measurements obtained in this study, while applying the same warning framework to both datasets, as summarized in **Table 1**. Compared with sparse temperature information available from a conventional BMS, high-density temperature sensing generally enables earlier detection of abnormal thermal behavior within the battery system and reduces

the risk of missed warnings in several failure scenarios. This advantage is particularly pronounced for localized thermal anomalies, because a limited number of temperature sensing points may fail to capture abnormal behavior confined to a small spatial region, whereas high-density temperature measurements substantially enhance the observability of both local thermal anomalies and spatial temperature heterogeneity. In addition, the fiber optic sensors employed in this work exhibit a faster thermal response than the thermocouples (see **Supplementary Fig. 3**), which further enhances the capability to capture transient thermal anomalies. These results demonstrate that increasing the spatial density of temperature measurements can substantially improve the timeliness and reliability of thermal runaway warning. The parameter settings of the warning framework when using the sparse temperature measurements available from a conventional BMS are provided in **Supplementary Table 3**.

Table 1 Performance of a given thermal-runaway warning strategy with different temperature inputs

Warning strategy		Warning time			
		Overheating	Overcharging	Vehicle 1	Vehicle 4
Sparse temperature data acquired by the BMS	Temperature-based warning	14:05:37	11:18:22	None	19:11:54
	SE-based warning	14:07:18	None	17:04:30	None
**High-density temperature data used in this work**	Temperature-based warning	13:53:42	11:15:21	17:02:32	19:11:29
	SE-based warning	13:53:43	11:10:50	17:02:41	19:11:42

In Supplementary Information:

Supplementary Table 3. Parameter settings of the warning framework using sparse temperature data as input

	Temperature warning threshold	dT/dt warning threshold	SE warning boundary				dSE/dt warning threshold
			a	b	c	E_{tol}	
Module-level	100	1	-1.91	0.09	3.96	0.3	0.2
Pack-level	100	1	-1.00	0.55	2.53		0.2

Comment 7:

More comparative results against existing thermal runaway warning methods are required to demonstrate the advantages of the proposed method.

Response: Thank you very much for this important suggestion. We agree that additional comparisons with existing thermal runaway warning methods are necessary to more comprehensively demonstrate the advantages of the proposed framework. Following your suggestion, we add a benchmark comparison with three representative thermal runaway warning strategies reported in the literature, including a temperature-based composite threshold strategy, a temperature variance–local overheating index (LOI) dual-threshold strategy, and a modified standardized cumulative sum (CUSUM) method. To ensure a fair comparison, all benchmark methods and the proposed framework are evaluated using the same high-density distributed temperature data. The comparison results are summarized in Table 2. The temperature-based composite threshold strategy successfully identifies all thermal runaway events, but its warning times are consistently later than those of the proposed framework. In contrast, the temperature variance–LOI dual-threshold strategy and the modified standardized CUSUM method fail to identify all thermal runaway scenarios. These results demonstrate that the proposed framework provides more favorable overall performance in terms of warning timeliness and detection capability across different failure scenarios. Detailed descriptions and parameter settings of the three benchmark methods are provided in Supplementary Note 4. All corresponding modifications are highlighted in yellow in the revised manuscript and are shown below.

In "Thermal runaway warning performance"

Collectively, these results disentangle the respective contributions of high-density temperature sensing and the multi-model collaborative warning framework to thermal runaway warning performance. High-density temperature sensing enhances the observability of localized and spatially heterogeneous thermal anomalies, whereas the proposed warning

framework further translates this sensing advantage into earlier and more reliable warning performance across different failure modes. The integration of high-density sensing with multi-model collaborative warning framework therefore provides a more reliable basis for thermal runaway early warning than conventional sparse temperature monitoring combined with a single warning strategy.

Table 2 Performance comparison of existing warning methods using distributed temperature sensing as input.

**Warning strategy**	**Warning time**			
	Overheating	Overcharging	Vehicle 1	Vehicle 4
Temperature-based composite threshold strategy [50]	13:53:45	11:15:21	17:03:00	19:11:52
Temperature variance and LOI dual-threshold warning [51]	14:05:31	None	None	None
Modified standardized CUSUM method [52]	13:53:43	11:18:24	None	None
**The proposed warning framework**	13:53:42	11:10:50	17:02:32	19:11:29

In Reference:

50 Yue, Y. et al. Early warning of thermal runaway in large-capacity LiFePO4 batteries based on distributed optical fiber. J. Energy Storage 141, 119280 (2026).

51 Wang, S. et al. Infrared imaging investigation of temperature fluctuation and spatial distribution for a large laminated lithium‑ion power battery. Applied Thermal Engineering 152, 204-214 (2019).

52 Vu, L. A. et al. A real-time framework for early detection and severity prediction of thermal runaway in Li-ion batteries. J. Energy Storage 135, 118310 (2025).

In Supplementary Information:

Supplementary Note 4. Details of the existing warning methods for performance comparison

Method 1: Temperature-based composite threshold strategy

Let $T_{i,t}$ denote the temperature measured at the i-th sensing point at time t, and let Ω_t denote the set of temperature sensing points at that time. The maximum and minimum temperatures of the battery system are defined as

$$T_{max,t} = \max_{i\epsilon\Omega_t} T_{i,t} \tag{1}$$

$$T_{min,t} = \min_{i\epsilon\Omega_t} T_{i,t} \tag{2}$$

The instantaneous temperature range, used here to quantify temperature non-uniformity across the battery system, is calculated as

$$\Delta T_{range,t} = T_{max,t} - T_{min,t} \tag{3}$$

This metric captures the temperature contrast between the hottest and coolest sensing points. Unlike a spatial temperature gradient, it does not require information on the physical distances between sensing points and can therefore accommodate different sensor numbers and spatial arrangements at the module and pack levels. To characterize the temporal evolution of temperature, the temperature rise rate at the i-th sensing point is calculated from two consecutive measurements using the actual elapsed time:

$$\mathrm{R}_{i,t_k} = \frac{T_{i,t_k} - T_{i,t_{k-1}}}{t_k - t_{k-1}} \tag{4}$$

To suppress short-term measurement fluctuations, a moving average over W consecutive sampling points is applied:

$$\overline{\mathrm{R}}_{i,t_k} = \frac{1}{W}\sum_{j=k-W+1}^{k} \mathrm{R}_{i,t_j} \tag{5}$$

The maximum temperature rise rate of battery system is subsequently obtained from all valid sensing points:

$$\mathrm{R}_{max,t_k} = \max_{i\epsilon\Omega_{t_k}} \overline{\mathrm{R}}_{i,t_k} \tag{6}$$

Following the composite-threshold logic of the reference method and its adaptation to the present datasets, a thermal runaway warning is issued when the maximum temperature exceeds its prescribed threshold and either the maximum temperature rise rate or the temperature range exceeds the corresponding threshold:

$$T_{max,t} \geq T_{th} \;\wedge\; \left[\mathrm{R}_{max,t} \geq R_{th} \;\vee\; \Delta T_{range,t} \geq \Delta T_{th}\right] \; = 1 \tag{7}$$

where T_{th}, R_{th}, and ΔT_{th} denote the thresholds for the maximum temperature, maximum temperature rise rate, and temperature range, respectively. Based on the normal operating data collected at the lab-scale module level and at pack level under real-world vehicle operation, the parameters of this method are calibrated to suppress false warnings under normal operation. The final parameter settings adopted for the battery module and battery pack are summarized below.

**Parameters**	**Module-level**	**Pack-level**
Moving average window $\boldsymbol{W}$	20	20
Maximum temperature threshold $\boldsymbol{T_{th}}$	100 ℃	100 ℃
Maximum temperature rise rate threshold $\boldsymbol{R_{th}}$	0.2 ℃/s	0.2 ℃/s
Temperature range threshold $\boldsymbol{\Delta T_{th}}$	20	20

Method 2: temperature variance and LOI dual-threshold warning

The temperature variance is employed to quantify the spatial non-uniformity of the distributed temperature field. At the t-th time step, the battery system contains M valid temperature sensing points, and the measured temperature at the j-th sensing point is denoted by $T_{t,j}$. The spatially averaged temperature is calculated as

$$\bar{T}_t = \frac{1}{M}\sum_{j=1}^{M} T_{t,j} \tag{8}$$

Accordingly, the temperature variance of the battery system is defined as

$$T_{var,t}^2 = \frac{1}{M}\sum_{j=1}^{M}\left(T_{t,j} - \bar{T}_t\right)^2 \tag{9}$$

where $T_{\mathrm{var},t}^2$ represents the dispersion of the distributed temperature measurements at the t-th time step. A larger temperature variance indicates a greater temperature difference among the sensing points and, consequently, a more non-uniform temperature field.

In addition to the numerical dispersion of the temperature field, the local overheating index (LOI) is introduced to characterize the spatial concentration of high-temperature sensing points. At each step, all valid temperature measurements are ranked in descending order, and the highest $\eta\%$ of the temperature sensing points are selected as the high-temperature subset. The sensing point with the maximum temperature is regarded as the hotspot center. Let $\left(x_{t,max}, y_{t,max}\right)$ denote the normalized spatial coordinates of the hotspot center and let $\left(x_{t,k}, y_{t,k}\right)$ denote the normalized coordinates of the k-th sensing point in the high-temperature subset. The LOi is calculated as

$$LOI_t = \frac{1}{N_h}\sum_{k=1}^{N_h}\sqrt{\left(x_{t,k} - x_{t,max}\right)^2 + \left(y_{t,k} - y_{t,max}\right)^2} \tag{10}$$

where N_h is the number of sensing points included in the high temperature subset. The spatial coordinates are normalized along each direction before calculation to eliminate the influence of the physical dimensions of the battery system. A smaller LOI value indicates that the high temperature sensing points are more strongly concentrated around the hotspot center, whereas a larger LOI value indicates a more dispersed high-temperature distribution.

Temperature variance and LOI characterize two complementary features of thermal anomalies. The temperature variance reflects the overall non-uniformity of the distributed temperature field, whereas the LOI describes the spatial concentration of the high temperature region. Therefore, a thermal anomaly is identified only when a significant increase in temperature non-uniformity is accompanied by pronounced local hotspot concentration. The instantaneous warning state at the t-th time step is defined as

$$A_t = \begin{cases} 1, & T_{var,t}^2 \geq T_{var,th}^2 \;\; and \;\; LOI_t \leq LOI_{th} \\ 0, & otherwise \end{cases} \tag{11}$$

where $T_{\mathrm{var,th}}^2$ is the upper warning threshold for temperature variance and $\mathrm{LOI_{th}}$ is the lower warning threshold for the local

overheating index. Based on the normal operating data collected at the lab-scale module level and at pack level under real-world vehicle operation, the parameters of this method are calibrated to suppress false warnings under normal operation. The final parameter settings adopted for the battery module and battery pack are summarized below.

**Parameters**	**Module-level**	**Pack-level**
Upper threshold of temperature variance $\boldsymbol{T^2_{var,th}}$	50	50
Lower threshold of local overheating index $\boldsymbol{LOI_{th}}$	0.2	0.2
Proportion of high-temperature sensing points $\boldsymbol{\eta}$	10%	10%

Method 3: Modified standardized CUSUM method

For the temperature signal $T_{i,t}$ measured by the i-th sensing point at time t, an adaptive mean is first constructed using an exponentially weighted moving average:

$$\mu_{i,t} = (1-\beta)\mu_{i,t-1} + \beta T_{i,t} \tag{12}$$

where $\mu_{i,t}$ is the adaptive mean temperature of the i-th sensing point at time t, and β is the mean updating coefficient. Subsequently, the sample standard deviation of the temperature fluctuations is calculated using a sliding window with a length of W:

$$\sigma_{i,t} = \sqrt{\frac{1}{W-1}\sum_{j=t-W+1}^{t}\left(T_{i,j} - \bar{T}_{i,t}\right)^2} \tag{13}$$

where $\bar{T}_{i,t}$ denotes the average temperature of the i-th sensing point within the current sliding window. Under some operating conditions, the temperature fluctuation may be extremely small, and direct standardization may excessively amplify minor disturbances. Therefore, a lower bound $\sigma_{\min}$ is introduced for the standard deviation, and the standardized temperature residual is calculated as:

$$z_{i,t} = \frac{T_{i,t} - \mu_{i,t}}{\max(\sigma_{i,t}, \sigma_{min})} \tag{14}$$

where $z_{i,t}$ represents the deviation of the current temperature from its recent normal operating state.
Based on the standardized residual, the positive CUSUM statistics are recursively updated as:

$$C^+_{i,t} = \max\left[0, C^+_{i,t-1} + z_{i,t} - k\right] \tag{15}$$

where $C^+_{i,t}$ is the positive CUSUM statistic of the i-th temperature sensing point at time t, and k is the reference drift parameter used to suppress the accumulation of minor positive temperature deviations during normal operation. When persistent abnormal heating occurs at a sensing point, its corresponding $C^+_{i,t}$ continuously increases. In contrast, when the temperature fluctuation remains within the normal range, the CUSUM statistics remain at a low level or returns to zero.

For a battery module or battery pack containing M valid temperature sensing points, the system-level temperature warning statistic is defined as:

$$C_{sys,t} = \max_{i=1,2,\cdots,M} C^+_{i,t} \tag{16}$$

A thermal runaway warning is triggered when the positive CUSUM statistic of any valid temperature sensing point reaches the predefined threshold H:

$$C_{sys,t} \geq H \tag{17}$$

Based on the normal operating data collected at the lab-scale module level and at pack level under real-world vehicle operation, the parameters of the modified standardized CUSUM method are calibrated to suppress false warnings under normal operation. The final parameter settings adopted for the battery module and battery pack are summarized below.

**Parameters**	**Module-level**	**Pack-level**
Mean updating coefficient $\boldsymbol{\beta}$	0.30	0.30
Length of sliding window $\boldsymbol{W}$	20	20
Minimum standard deviation $\boldsymbol{\sigma_{min}}$	0.05 ℃	0.05 ℃
Reference drift parameter $\boldsymbol{k}$	0.50	0.50
Warning threshold $\boldsymbol{H}$	7.00	30.00

Comment 8:

More explanations on Fig. 1d are needed, how does the authors obtain the decreasing curve with the numbers need to be clarified.

Response: Thank you for pointing out that the derivation of the decreasing cost trend in Fig. 1(d) is not sufficiently explained. Following your suggestion, we clarify that the cost at the reference scale of 10^4 vehicles is obtained from a supplier quotation based on the actual hardware configuration used in this study. The costs at larger deployment scales are then projected using a component-level technology learning-curve model, in which the unit cost decreases as a power-law function of cumulative deployment. Learning rates of 10% and 5% are assigned to the core sensing hardware and auxiliary components, respectively. Based on this model, the per-vehicle hardware costs at deployment scales of 10^4, 10^5, 10^6, and 10^7 vehicles are calculated as 103.17, 73.10, 51.85, and 36.82 USD, respectively, which generates the decreasing trend shown in Fig. 1(d). The detailed calculation equation, assumptions, cost breakdown, and references are added in Supplementary Note 1. All corresponding modifications are highlighted in yellow in the revised manuscript and are given below.

In Supplementary Information:

Furthermore, this study adopts a component-level technology learning-curve model to estimate the cost evolution of the proposed warning scheme with increasing deployment scale. This model is appropriate because the system mainly consists of standardized hardware components whose unit costs can decrease through cumulative manufacturing experience, process automation, yield improvement, and economies of scale [1]. Technology learning curves, which describe unit cost as a power-law function of cumulative production or deployment, have been widely used for energy and battery technology cost forecasting [2]. For the i-th hardware component, the unit cost at a cumulative deployment scale Q is expressed as:

$$C_{i,Q} = C_{i,Q_0}(Q/Q_0)^{\ln(1-LR_i)/ln2}$$

where $C_{i,Q}$ and C_{i,Q_0} are the per-vehicle cost of component i at deployment scale Q and Q_0, respectively. LR_i is the learning rate of component i.

Since the historical cost data across multiple production scales are not currently available for the proposed warning scheme, the learning rates adopted in this analysis are treated as scenario-based assumptions rather than parameters empirically identified from historical cost data. In its component-level technology cost projections, the International Energy Agency applies a learning rate of 10% to automotive battery cells and fuel cell stacks and a learning rate of 5% to battery packaging and fuel cell balance-of-plant components [3]. Based on these analogous component categories, a learning rate of 10% is assigned to the temperature sensing gratings and onboard miniaturized interrogator, while a learning rate of 5% is assigned to cables and accessories, and installation materials in this study. Building upon this, the projected per-vehicle costs at deployment scales of 10^4, 10^5, 10^6, and 10^7 vehicles are summarized below.

**Deployment scales**	**Temperature sensing gratings**	**Interrogator**	**Cables and accessories**	**Installation materials**	**Total cost**
10^4 vehicles	64.80 USD	35.50 USD	2.15 USD	0.72 USD	103.17 USD
10^5 vehicles	45.66 USD	25.02 USD	1.81 USD	0.61 USD	73.10 USD
10^6 vehicles	32.18 USD	17.63 USD	1.53 USD	0.51 USD	51.85 USD
10^7 vehicles	22.68 USD	12.42 USD	1.29 USD	0.43 USD	36.82 USD

3 Unit cost reductions for selected technologies in the Sustainable Development Scenario, 2020 – 2070, https://www.iea.org/data-and-statistics/charts/unit-cost-reductions-for-selected-technologies-in-the-sustainable-development-scenario-2020-2070

Authors' Responses

Manuscript Number: NCOMMS-26-010836B

Title: Multi-model collaborative early warning for battery systems with integrated fiber optic sensors

Dear Editor and Reviewers:

We thank the editor and reviewers for their positive assessment of our manuscript. We have revised the manuscript and accompanying files to address the editorial requirements. Our point-by-point responses to the comments are provided below.

Yours sincerely,
On behalf of all authors,

Jiangong Zhu, Ph.D
Professor
College of Automotive and Energy Engineering,
Clean Energy Automotive Engineering Center,
Tongji University

Responses to Reviewer #1:

accept

Response: Thank you for your valuable comments and positive feedback on our manuscript. We greatly appreciate it.

Responses to Reviewer #2:

All comments have been well addressed.

Response: Thank you very much for your positive assessment and constructive feedback, which have helped improve our manuscript.
